# Déjà vu: a reappraisal of the taphonomy of quarry VM4 of the Early Pleistocene site of Venta Micena (Baza Basin, SE Spain)

**DOI:** 10.1038/s41598-021-04725-3

**Published:** 2022-01-13

**Authors:** Paul Palmqvist, M. Patrocinio Espigares, Juan A. Pérez-Claros, Borja Figueirido, Antonio Guerra-Merchán, Sergio Ros-Montoya, Guillermo Rodríguez-Gómez, José Manuel García-Aguilar, Alejandro Granados, Bienvenido Martínez-Navarro

**Affiliations:** 1 Departamento de Ecología y Geología, Universidad de Málaga, Campus de Teatinos, 29071 Málaga, Spain; 2 Departamento de Geodinámica, Estratigrafía y Paleontología, Universidad Complutense de Madrid, C/ José Antonio Novais 12, 28040 Madrid, Spain; 3 Centro UCM-ISCIII de Evolución y Comportamiento Humanos, Avd/ Monforte de Lemos, 5, Pabellón 14, 28029 Madrid, Spain; 4 IPHES-CERCA, Institut Català de Paleoecologia Humana I Evolució Social, C/ Marcel.Lí Domingo S/N, Campus Sescelades, Edifici W3, 43007 Tarragona, Spain; 5 Area de Prehistoria, Universitat Rovira I Virgili (URV), Avda. Catalunya 35, 43002 Tarragona, Spain; 6 ICREA, Pg. Lluís Companys 23, 08010 Barcelona, Spain

**Keywords:** Palaeoecology, Palaeoecology

## Abstract

Venta Micena, an Early Pleistocene site of the Baza Basin (SE Spain), preserves a rich and diverse assemblage of large mammals. VM3, the main excavation quarry of the site, has been interpreted as a den of the giant hyaena *Pachycrocuta brevirostris* in the plain that surrounded the Baza palaeolake. Taphonomic analysis of VM3 has shown that the hyaenas scavenged the prey previously hunted by the hypercarnivores, transported their remains to the communal den, and consumed the skeletal parts according to their marrow contents and mineral density. In a recent paper (Luzón et al. in Sci Rep 11:13977, 10.1038/s41598-021-93261-1, 2021), a small sample of remains unearthed from VM4, an excavation quarry ~ 350 m distant from VM3, is analysed. The authors indicate several differences in the taphonomic features of this assemblage with VM3, and even suggest that a different carnivore could have been the agent involved in the bone accumulation process. Here, we make a comparative analysis of both quarries and analyse more skeletal remains from VM4. Our results indicate that the assemblages are broadly similar in composition, except for slight differences in the frequency of megaherbivores, carnivores and equids according to NISP values (but not to MNI counts), the degree of bone weathering, and the intensity of bone processing by the hyaenas. Given that VM4 and VM3 were not coeval denning areas of *P. brevirostris*, these differences suggest that during the years when the skeletal remains were accumulated by the hyaenas at VM3, the rise of the water table of the Baza palaeolake that capped with limestone the bones was delayed compared to VM4, which resulted in their more in-depth consumption by the hyaenas.

## Introduction

**Figure 1:**
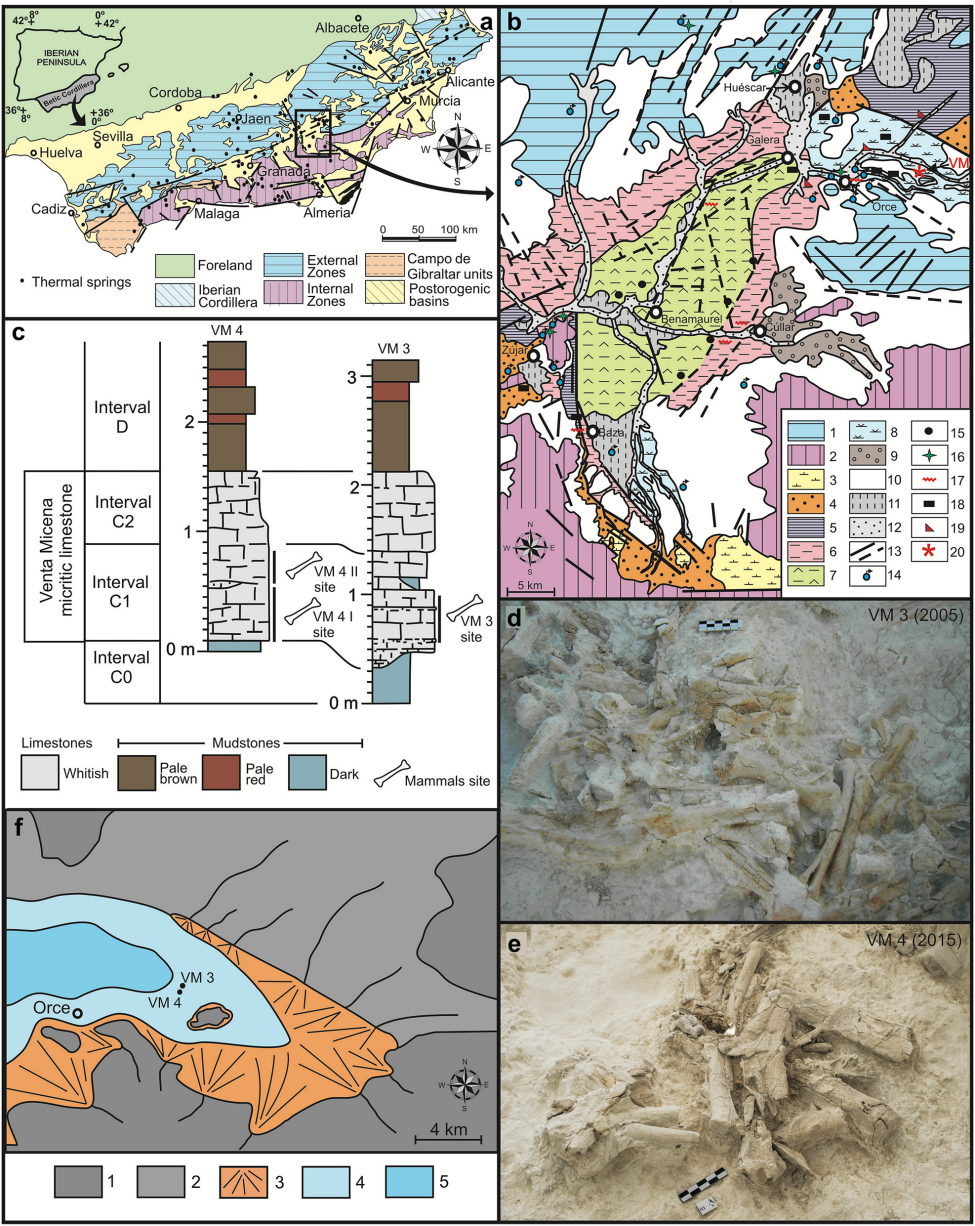
(**a**) Geological context of the Guadix-Baza Depression in the Betic Cordillera, SE Spain. The box encloses the Baza subbasin. The points indicate the thermal springs (N = 122) that are nowadays active in the Betic Cordillera, with a range of water output temperatures of 18–60 °C. (**b**) Tectono-sedimentary map of the Baza subbasin with indication of those points that preserve geochemical, mineralogical, or lithological evidence of thermal activity during the Neogene-Quaternary. 1, External Zones; 2, Internal Zones; 3, Tortonian marine deposits; 4, Plio-Pleistocene alluvial and fluvial deposits; 5, Late Turolian–Ruscinian lacustrine deposits; 6, Middle Villafranchian fluvio-lacustrine deposits; 7, Middle Villafranchian lacustrine marls and evaporites; 8, Late Villafranchian lacustrine deposits; 9, Middle Pleistocene alluvial and lacustrine deposits; 10, Late Pleistocene glacis surface; 11, Holocene fluvial terrace; 12, Modern fluvial sediments; 13, Faults (solid lines; striped lines indicate those faults covered by sediments that have been inferred from aerial photographs); 14, Thermal springs nowadays active; 15, Sulphur deposits; 16, Travertine buildings; 17, Palaeoseismites; 18, Black levels; 19, Silexites; 20, Location of the Venta Micena site. (**c**) Stratigraphic series of VM4 and VM3 quarries. (**d**) View of one grid of quarry VM3 during the summer excavation of 2005, which was codirected by Martínez-Navarro and P. Palmqvist. (**e**) View of one grid of quarry VM4 during the summer excavation of 2015. (**f**) Palaeogeographical context of the Orce-Venta Micena sector of the Baza Basin during the Late Villafranchian [1, External Zones; 2, Pliocene sediments (emerged relief); 3, Alluvial and fluvial sediments; 4, Extension of the lake during a highstand stage; 5. Extension of the lake in a lowstand stage]. The location of excavation quarries VM4 and VM3 of Venta Micena is shown. The maps depicted in figures (**a,b**) were generated using the software CorelDRAW Suite X4 (https://www.coreldraw.com).

VM4^1^ is an excavation quarry of the Early Pleistocene (Calabrian, Late Villafranchian) site of Venta Micena (VM), which lies in the NE sector of the Baza Basin (Guadix-Baza Depression, Province of Grenade, SE Spain; Fig. 1). This inland basin preserves a thick (> 400 m) and relatively continuous record of continental sediments of Plio-Pleistocene age composed of lacustrine and fluvial deposits, as well as dark clays and silexites associated to hot springs. Hydrothermal activity (Fig. 1a,b) provided a mild and productive environment for the terrestrial fauna, which remains were preserved in many fossil localities across the basin^2–4^. VM preserves a worldwide unique fossil record (Fig. 1d–f): for example, > 24,000 skeletal remains of large mammals have been unearthed from a surface of ~ 400 m^2^ during the last decades in several excavation quarries, including VM2, VM3, and VM4, which represents a mean density of fossils of > 60/m^2^^5–11^. Although this density is not homogeneously recorded across the 80–120 cm thick VM stratum, which outcrops along ~ 2.5 km^12^, it suggests that tens of millions of fossils were preserved in the micritic limestones of the lithological unit^7^.

During the last decades, taphonomic studies of VM3, the main quarry excavated at the site^4–10,13^, have shown that: (i) the excavated surface (~ 220 m^2^) has provided 6331 identifiable skeletal remains from 339 individuals distributed among 24 mammalian taxa, 1819 anatomically identifiable bones that could not be determined taxonomically (e.g., diaphyseal fragments and small cranial fragments) and several thousands of bone shafts (Table 1); (ii) the fossils range in size from isolated teeth and phalanges of small carnivores to mandibles of elephants; (iii) girdle/limb bones and, to a lesser extent, cranial elements predominate over ribs and vertebrae; (iv) herbivore taxa dominate the assemblage, both in number of identifiable specimens (NISP) and minimal number of individuals (MNI); (v) the age estimated for the individuals includes calves and juveniles with deciduous teeth as well as prime and past prime adults with fully erupted permanent dentition; (vi) more common herbivores, such as horse *Equus altidens* and megacerine deer *Praemegaceros* cf. *verticornis,* show high frequencies of non-adults, > 45% in both cases; and (vii) among carnivores, only adult individuals have been recovered, with the exception of the giant (~ 110 kg)^10^, short-faced hyaena *Pachycrocuta brevirostris,* which is represented by 50% of non-adults, and a few juveniles of the wild dog *Lycaon lycaonoides*, the wolf *Canis orcensis* and the bear *Ursus etruscus* (Table 1).

**Table 1:** Number of identified specimens (NISP) and minimal number of individuals (MNI) of herbivore and carnivore species in Venta Micena (data for the excavation quarry of VM4 published by Luzón et al.^1^, which include the fossils unearthed during the years 2005 and 2019–2020; data for VM3 updated from Ref.^9^).

Species	Venta Micena-4				Venta Micena-3			
	NISP	MNI (cj/as)	% Non-adults	CI (p = 0.05)	NISP	MNI (cj/as)	% Non-adults	CI (p = 0.05)
*Mammuthus meridionalis*	4	2 (1/1)	50.0	1.3– 98.7	58	5 (4/1)	80.0	28.4–99.5
*Stephanorhinus* aff. *hundsheimensis*	14	5 (4/1)	80.0	28.4– 99.5	103	7 (4/3)	57.1	18.4–90.1
*Equus altidens*	124	12 (7/5)	58.3	27.7– 84.8	2937	91 (51/40)	56.0	45.3–66.4
*Hippopotamus antiquus*	17	1 (0/1)	0.0	0.0– 97.5	63	5 (3/2)	60.0	23.1–88.2
*Bison* sp.	43	3 (1/2)	33.3	0.8–90.6	831	51 (23/28)	45.1	31.1–59.7
*Hemibos* aff. *gracilis*	4	1 (0/1)	0.0	0.0–97.5	1	1 (0/1)	0.0	0.0–97.5
*Soergelia minor*	13	1 (0/1)	0.0	0.0–97.5	302	20 (4/16)	20.0	5.7–43.7
*Hemitragus albus*	19	3 (1/2)	33.3	0.8–90.6	285	16 (2/14)	12.5	1.6–38.6
*Praeovibos* sp.	–	–	–	–	1	1 (0/1)	0.0	0.0–97.5
Bovidae indet., small size (cf. *Rupicapra*)	–	–	–	–	1	1 (0/1)	0.0	0.0–97.5
*Praemegaceros* cf. *verticornis*	61	7 (3/4)	42.9	9.9–81.6	881	56 (26/30)	46.3	33.0–60.3
*Metacervocerus rhenan*us	35	6 (2/4)	33.3	4.3–77.7	460	33 (9/24)	27.3	13.3–45.5
Cervidae indet., small size (cf. *Capreolus*)	–	–	–	–	1	1 (0/1)	0.0	0.0–97.5
Herbivore indet.	1158	–	–	–	646	–	–	–
Total herbivores	1492	41 (19/22)	47.4	31.0–64.2	6570	287 (125/162)	43.6	37.7–49.5
*Homotherium latidens*	1	1 (0/1)	0.0	0.0–97.5	15	2 (0/2)	0.0	0.0–84.2
*Megantereon whitei*	1	1 (0/1)	0.0	0.0–97.5	52	3 (0/3)	0.0	0.0–70.8
*Panthera* cf. *gombaszoegensis*	1	1 (0/1)	0.0	0.0–97.5	1	1 (0/1)	0.0	0.0–97.5
*Lynx* cf. *pardinus*	3	1 (0/1)	0.0	0.0–97.5	12	2 (0/2)	0.0	0.0–84.2
*Pachycrocuta brevirostris*	15	2 (0/2)	0.0	0.0–84.2	122	18 (9/9)	50.0	26.0–74.0
Viverridae indet	–	–	–	–	1	1 (0/1)	0.0	0.0–97.5
*Lycaon lycaonoides*	8	2 (0/2)	0.0	0.0–84.2	63	9 (1/8)	11.1	0.3–48.3
*Canis orcensis*	15	1 (0/1)	0.0	0.0–97.5	96	8 (1/7)	12.5	0.3–52.7
*Vulpes alopecoides*	1	1 (0/1)	0.0	0.0–97.5	11	2 (0/2)	0.0	0.0–84.2
*Ursus etruscus*	11	1 (0/1)	0.0	0.0–97.5	33	4 (1/3)	25.0	0.6–80.6
*Meles meles*	–	–	–	–	1	1 (0/1)	0.0	0.0–97.5
Carnivore indet	30	–	–	–	47	–	–	–
Total carnivores	86	11 (0/11)	0.0	0.0–28.5	454	52 (12/40)	22.6	12.3–36.2
Large mammal indet.	–	–	–	–	1126	–	–	–

This table shows the abundance of young individuals (i.e., calves and juveniles: cj) and adults (i.e., prime adults and senile individuals: as). For each species, the percentage of calves and juvenile individuals over the MNI count shows the 95% confidence interval (CI) estimated using a binomial approach: p ± z[p(1 − p)/n]^1/2^, where *p* is the proportion of successes in a Bernoulli trial process and *z* is the 1 − α/2 quantile of a standard normal distribution.

**Figure 2:**
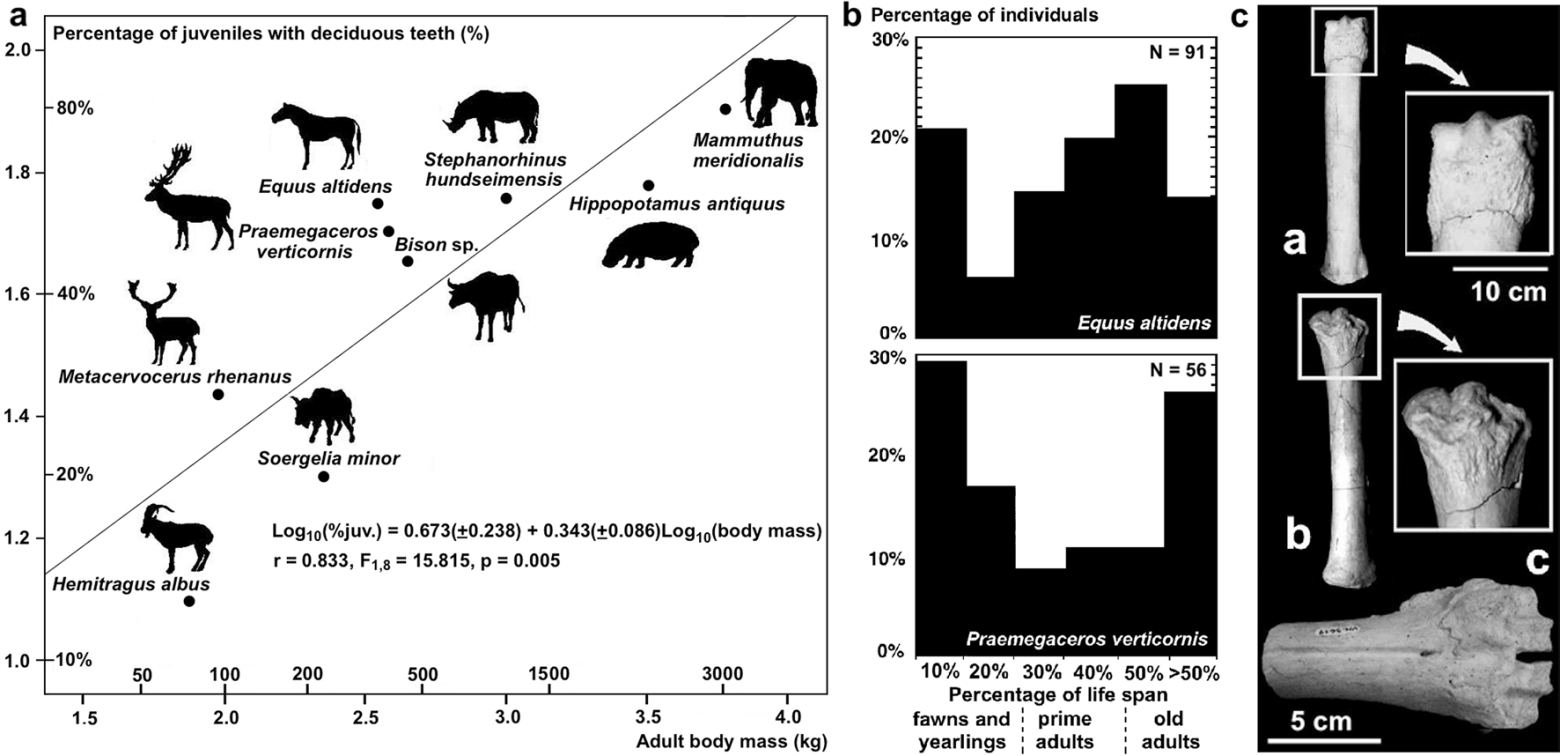
Evidence of prey selection in VM3. (**a**) Least-squares regression between the logarithms of the mean body masses (X-axis) of adult individuals of the herbivore species of Venta Micena, estimated from postcranial measurements (data from Refs.^6,14^), and the logarithms of the percentage of calves and juvenile individuals over the MNI estimates of each species (Y-axis), calculated from teeth counts (data from Table 1). (**b**) Mortality profiles deduced from crown height measurements in horse *Equus altidens* and megacerine deer *Praemegaceros verticornis* (updated from Ref.^6^). (**c**) Three examples of osteopathologies detected in VM3 ((**a**) horse metatarsal with osteophytic overgrowths in the distal epiphysis; (**b**) horse metacarpal showing an intense deformation of the distal epiphysis; (**c**) severe osteoarthritis in a third-four metacarpal of megacerine deer).

Taphonomic analyses have shown that *P. brevirostris* was the bone accumulating agent at VM3 and that most losses of palaeobiological information were a consequence of the selective destruction of skeletal remains by the hyaenas during the period when the bones were exposed before burial^7–10^. Analysis of mortality patterns for ungulate species deduced from juvenile/adult proportions and tooth-wearing classes^6^ indicates that the hyaenas scavenged the skeletal remains from carcasses of animals previously hunted upon by hypercarnivores such as sabre-tooths *Homotherium latidens* and *Megantereon whitei,* jaguar *Panthera* cf. *gombaszoegensis,* and wild dog *Lycaon lycaonoides.* This inference is based on: (i) the positive relationship in herbivorous taxa between the percentage of juveniles and the body mass estimated for the adults, which indicates the selection of young, more vulnerable individuals in the largest prey species (Fig. 2a); (ii) the finding of U-shaped, attritional mortality profiles in the ungulates better represented in the assemblage: the megacerine deer and, with a discrete frequency of very old adults, also the horse (Fig. 2b); and (iii) the presence of abundant autopodial bones with osteopathologies that limited the ability of the animals to escape from predators (Fig. 2c)^6,7,9^. Analysis of skeletal representation for ungulate taxa in VM3 has shown that the hyaenas selectively transported herbivore carcasses and body parts to their maternity den as a function of the mass of the ungulates scavenged. This resulted in the transport as whole carcasses of small-to-medium sized species like goat *Hemitragus albus* and fallow deer *Metacervocerus rhenanus,* while in the case of large-sized species (e.g., horse and *Bison* sp.) the carcasses were dismembered by the hyaenas, which preferentially transported the limbs that provided larger marrow yields^8^. The selective transport of certain anatomical parts suggests that each short-faced hyaena foraged alone in search of scavengeable carcasses, as do modern brown hyaenas: if they had foraged in groups, as spotted hyaenas often do, the members of the hyena clan would have transported all the anatomical regions of each carcass to their maternity den^8^. Later, the fracturing and consumption of major limb bones by the hyaenas at their den was highly selective, correlating with their marrow contents and mineral density. This resulted in well-defined patterns of consumption for the limb bones (e.g., a proximodistal sequence in the humerus and tibia, and a distoproximal one in the radius, femur, and metapodials)^7,9,10^. As a result of these taphonomic biases, the assemblage records marked differences in the abundance of different skeletal remains from each ungulate species as well as among taxa.

**Figure 3:**
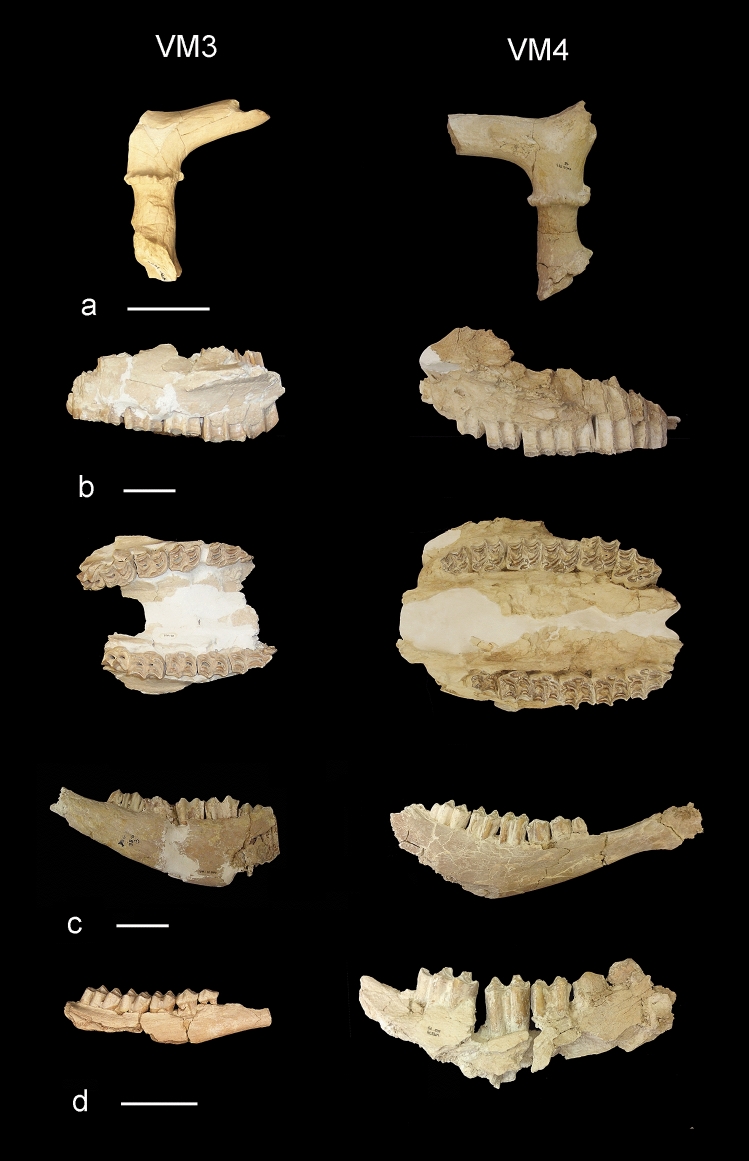
Cranial remains from quarries VM3 (left) and VM4 (right) of Venta Micena showing similar patterns of consumption by the hyaenas^6–10^. (**a**) Antler fragments of *Metacervocerus rhenanus.* (**b**) Maxillae of *Equus altidens* in labial (upper photographs) and occlusal views (lower photographs). (**c**) Jaw fragments of *Bison* sp. (**d**) Jaw fragments of *M. rhenanus* (left) and *Bison* sp. (right). Scale bars represent 5 cm.

**Figure 4:**
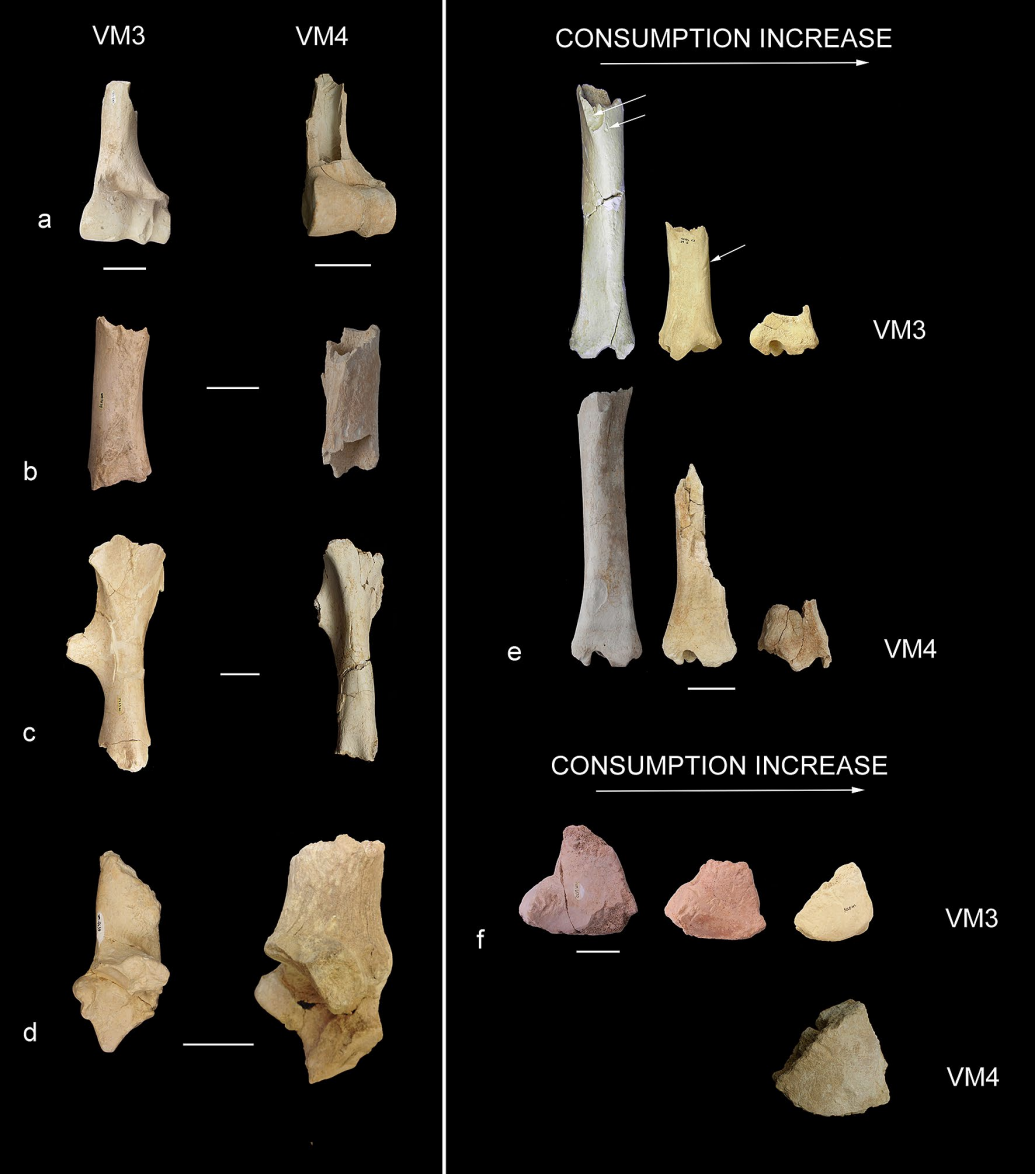
Postcranial remains from quarries VM3 (left) and VM4 (right) of Venta Micena showing similar patterns of bone consumption by the hyaenas^6–10^. (**a**) Distal humeri of *Bison* sp. (left) and *Equus altidens* (right). (**b**,**c**) Femur diaphyseal fragments of large sized herbivores. (**d**) Calcanei of Bovini (left) and *Hippopotamus antiquus* (right). (**e**) Sequences of consumption of tibiae from large-sized ungulates (upper sequence: VM3, lower sequence: VM4). (**f**) Sequences of consumption of lunate bones of *Mammuthus meridionalis* (upper sequence: VM3, lower sequence: VM4; the difference of size is because the lunate of VM4 is from an adult individual while those of VM3 are from juveniles). Scale bars represent 5 cm.

In a recent paper, Luzón et al.^1^ address the taphonomy of VM4, focusing their study on a subset of the bone assemblage unearthed from this quarry, which taphonomic features compare with those published for VM3^6–10,13^. Despite the overall similarity between the bone assemblages of VM4 and VM3 (Table 1, Figs. 3, 4), Luzón et al.^1^ contribute interesting new data for VM4, which we discuss in detail below, and indicate several differences with respect to the assemblage preserved at VM3, which was conclusively accumulated and modified by *P. brevirostris*^6–10^. Moreover, they even suggest the possibility that a different carnivore was the taphonomic agent involved in the site formation process at VM4^1^. For this reason, we perform here a comparative taphonomic study of VM4 and VM3, to shed light on the similarities and differences between the bone assemblages from both quarries, as well as on the bone accumulating and modifying agent at VM4.

In this paper, we: (i) compare the taphonomic data provided by Luzón et al.^1^ for VM4 with those available in the larger assemblage from VM3, and also with new data from an analysis by M.P. Espigares of 3729 fossils unearthed from VM4 during the years 2013–2015; (ii) discuss on the taphonomic agent responsible of the accumulation and modification of skeletal remains at VM4; (iii) provide clues on the palaeoecology of the assemblages of large mammals from VM4 and VM3; and (iv) propose a new model for the generation of the huge amount of fossils preserved across the VM stratum. Due to space limitations, the points (ii) and (iii) are addressed in the Supplementary Information.

## Results: taphonomy of the VM4 bone assemblage

### Patterns of species abundance

In their analysis of the fossil assemblage of VM4, Luzón et al.^1^ indicate that herbivorous taxa comprise the bulk of the fauna. Their data, compiled in Table 1, show that herbivore remains represent 94.2% (1492/1578) of NISP and 78.8% (41/52) of MNI values for large mammals. These figures are close to those of VM3, 93.5% (6570/7027) and 84.4% (287/340), respectively (Table 1). A χ^2^ test shows that these differences are not statistically significant (*p* > 0.3 in both cases). Among herbivores, Luzón et al.^1^ indicate that *E. altidens* is the species most abundantly preserved, both in frequency of remains and number of individuals, followed by cervids, bison, caprines, and megaherbivores (i.e., elephant, rhino, and hippo). This is also the situation in VM3 according to data compiled in Table 1: for example, the NISP value of *E. altidens* represents 31.8% (124/390) of the remains of large mammal identified in VM4 and 49.6% (2937/5924) in VM3. Although this difference is statistically significant (χ^2^ = 46.408, *p* < 0.0001), the frequencies of horse based on MNI estimates, 29.3% (12/41) in VM4 and 31.7% (91/287) in VM3, are similar (χ^2^ = 0.096, *p* > 0.75). The difference based on NISP values seems high, but it falls within the range expected from variations in abundance data from different years for the ungulate prey more common in Serengeti, where the frequencies of Thomson’s gazelle, wildebeest, and zebra fluctuated in the late sixties between 18.9–56.3%, 21.3–42.8%, and 11.1–15.7%, respectively^15,16^. Finally, *P. brevirostris* is the species most represented among carnivores in both assemblages according to NISP values (Table 1), 26.8% (15/56) in VM4 and 30.0% (122/407) in VM3 (χ^2^ = 0.241, *p* > 0.6), followed by canids, ursids and felids.

The distribution of NISP and MNI values among taxa in VM4 and VM3 was further analysed using an approach based on contingency tables. The table for NISP values shows a significant χ^2^ value (Table 2, left part). This results from some differences in taxa abundance between the assemblages compared, which are reflected in the adjusted residuals: remains of megaherbivores and carnivores (excluding hyaenas) are represented in VM4 by higher frequencies than those expected from a random, homogeneous distribution, while they are underrepresented in VM3. This applies to the estimates obtained for VM4 using the data of Luzón et al.^1^ and our own data (Tables S1, S2). The NISP values estimated for *P. brevirostris* by Luzón et al.^1^ suggest a higher frequency of this carnivore in VM4 than in VM3, as indicated by the adjusted residual. However, the abundance of hyaena remains in our dataset for VM4 does not depart significantly from the expectations, as happens in VM3. Given that the database of Luzón et al.^1^ includes less than half of the remains of large mammals included in our database (Table 2), this suggests that the high frequency of *P. brevirostris* reported in VM4 results from poor sampling. The remains of other carnivores are more abundantly represented in VM4 than in VM3. However, it must be noted that a study of 24 dens of the three living hyaenas showed that the abundance of carnivore remains is highly variable, even among dens of the same species^17^. The distribution of MNI values among taxa in VM4 and VM3 (Table 2, right part) does not differ from the expectations of a random distribution according to the low χ^2^ value of the contingency table. Only the adjusted residual for megaherbivores, which are slightly over-represented in VM4 according to the data of Luzón et al.^1^, is statistically significant, while their abundance in VM3 is slightly lower than expected. Moreover, the probabilities of obtaining in the randomization tests the cumulative χ^2^ values observed for the NISP and MNI values of each species (*p* < 0.001 and *p* > 0.97, respectively; Fig. S4) are equivalent to those obtained with their groupings in Table 2.

**Table 2:** Contingency tables for the abundance of large mammals in the assemblages of the two excavation quarries of Venta Micena compared in this study, VM4 (a: data published by Luzón et al.^1^ for the fossils unearthed during the years 2005 and 2019–2020; b: unpublished data analysed by M.P. Espigares for the fossils of 2005 and 2013–2015) and VM3 (updated from Ref.^9^).

Taxa	NISP values				MNI values			
	VM4 (a)	VM4 (b)	VM3	Σ partial rows	VM4 (a)	VM4 (b)	VM3	Σ partial rows
Megahervivores (*Mammuthus meridionalis* + *Hippopotamus antiquus* + *Stephanorhinus* aff. *hundsheimensis*)	35 (17.68) 4.326***	68 (40.88) 4.633***	224 (268.56) − 3.018**	327	8 (3.47) 2.667**	6 (4.94) 0.536	17 (22.65) − 2.360*	31
*Equus altidens*	124 (183.42) − 6.010***	331 (424.05) − 6.436***	2,937 (2785.83) 4.144***	3,392	12 (13.78) − 0.595	20 (19.62) 0.110	91 (89.86) 0.268	123
Large bovids (*Bison* sp. + *Hemibos* aff. *gracilis* + *Praeovibos* sp.)	47 (51.37) − 0.669	70 (118.77) − 5.106***	833 (780.23) 2.190*	950	4 (7.40) − 1.431	9 (10.53) − 0.554	53 (48.22) 1.428	66
Caprines (*Soergelia minor* + *Hemitragus albus*)	32 (35.31) − 0.599	34 (81.64) − 5.889***	587 (536.31) 2.484*	653	4 (5.04) − 0.519	5 (7.18) − 0.932	36 (32.88) 1.101	45
*Praemegaceros* cf. *verticornis*	61 (59.32) 0.242	155 (137.14) 1.759	881 (900.96) − 0.779	1097	7 (8.63) − 0.644	14 (12.28) 0.586	56 (56.26) − 0.072	77
*Metacervocerus rhenan*us	35 (29.58) 1.063	52 (68.38) − 2.197*	460 (449.25) 0.571	547	6 (4.93) 0.537	5 (7.02) − 0.873	33 (32.15) 0.304	44
*Pachycrocuta brevirostris*	15 (8.87) 2.140*	27 (20.50) 1.550	122 (134.69) − 1.201	164	2 (2.69) − 0.458	4 (3.83) 0.099	18 (17.50) 0.237	24
Other carnivores	71 (34.45) 6.684***	234 (79.64) 19.300***	332 (525.17) − 9.474***	637	9 (6.05) 1.353	11 (8.61) 0.944	34 (39.45) − 1.774	54
Σ partial columns	420	971	6376	ΣΣ = 7767 χ^2^ = 570.43***	52	74	338	ΣΣ = 464 χ^2^ = 15.65–

Separate tables are provided for the number of identifiable specimens (NISP, left table) and the estimates of minimum number of individuals (MNI, right table). The cells of each contingency table show the observed frequencies (OF), the frequencies expected from a random distribution (EF, between brackets), the adjusted residuals (normal deviates) and the level of statistical significance according to a two-tailed t-test (^–^p > 0.05,*p < 0.05,**p < 0.01,***p < 0.001). The tables also include the cumulative χ^2^-values [Σ_i_Σ_j_ (OF − EF)^2^/EF] with (r − 1) (c − 1) = 14 degrees of freedom.

In summary, the comparison of the faunal assemblages from both excavation quarries (Tables 1, 2) only shows some minor differences in taxa abundance for horse, megaherbivores, and carnivores other than the hyaena, as well as the presence in VM3 of some remains of two small ungulates (a roe deer-sized cervid and a chamois-sized bovid) and two small carnivores (Table 1), which are not reported by Luzón et al.^1^. Given their comparatively low number of specimens studied at VM4, it is reasonable to expect that the latter taxa, which are poorly represented in VM3, will also appear in VM4 during future excavations.

### Age mortality profiles

Luzón et al.^1^ indicate that two megaherbivores, elephant *Mammuthus meridionalis* and rhino *Stephanorhinus* aff. *hundsheimensis,* show frequencies of non-adults that are close to, or even higher than, those of adults, as happens in VM3 (Table 1). However, the low MNI counts for these species in VM4 do not allow to state this: for example, elephants are represented by a juvenile and an adult, which gives a frequency of 50% of non-adults; with a sample size of only two individuals, the 95% confidence interval calculated with a binomial approach for this percentage is 1.3–98.7% (Table 1). In *S. hundsheimensis,* the frequency of non-adults, 80% (4/5), has also a very wide confidence interval (28.4–99.5%). In three species of medium-to-large sized ungulates, *E. altidens,* the ancestor of water buffalo *Hemibos* aff. *gracilis* and *P. verticornis,* Luzón et al.^1^ report similar frequencies of adults and non-adults, while they indicate that *Bison* sp. shows a lower frequency of juveniles (Table 1). This is true for horse and deer (58.3% and 42.9% of non-adults, respectively), but *Hemibos* is only recorded by one adult individual, which means that the percentage of non-adults for this species is not reliable. Luzón et al.^1^ calculate the percentage of 33% non-adult bison over a sample of only three individuals, of which one is a juvenile: the confidence interval for non-adults (0.8–90.6%) comprises the frequencies for horse and megacerine deer (Table 1), which rules out their suggestion of a lower frequency of juveniles for this bovid. In contrast to VM4, the abundances of non-adult horse, bison and megacerine deer are similar in VM3 (Table 1), where they are represented by higher MNI counts (which makes their percentages reliable). A similar reasoning can be applied to the claim of Luzón et al.^1^ that adults outnumber calves and juveniles among smaller herbivores such as the Ovibovini *Soergelia minor,* the Caprini *Hemitraus albus* and the cervid *Metacervocerus rhenanus:* in these species, MNI counts are very low to calculate reliably the percentage of juveniles (see their confidence intervals in Table 1). In fact, Luzón et al.^1^ acknowledge this limitation when they write that “the total number of individuals in each species is too low to draw reliable conclusions on the resulting patterns” and “a prime-dominant, L- or U-shaped mortality profile cannot be clearly discerned”. The situation in VM3 is quite different (Table 1): MNI counts for the two ungulates better represented in the assemblage, *E. altidens* and *P. verticornis,* allowed to reconstruct U-shaped attritional mortality profiles (Fig. 2b), which evidenced that the hypercarnivores focused on young and old individuals in the case of large prey^6,7^.

### Patterns of skeletal abundance

The limitations and inaccuracies cited above result from the small sample analysed by Luzón et al.^1^ in VM4 (1578 remains of large mammals of which only 420 could be determined taxonomically and anatomically, compared to 8150 and 6331 remains in VM3, respectively: Table 1). These limitations apply also to their inferences on the skeletal profiles of ungulates. For example, they indicate that species of herbivore size class 2 (50–125 kg: *M. rhenanus, H. albus,* and *S. minor*) show biased skeletal profiles, with a predominance of teeth and elements of the forelimb over those of the hindlimb. In VM3, these ungulates also show higher frequencies of teeth than of bones, which has been interpreted as evidence of the transport by *P. brevirostris* of small-to-medium sized ungulates as whole carcasses to their denning site, where the giant hyaenas fractured the bones for accessing their medullary cavities and this resulted in their underrepresentation compared to teeth^7–10^. In the case of the major limb bones of these species in VM4, the elements of the forelimb (12.9%, 13 bones out of 101 determined remains) are twice as abundant as those of the hindlimb (6.9%, 7 bones), but these percentages do not differ statistically (χ^2^ = 2.028, *p* = 0.1544), which indicates the effects of poor sampling. In the species of herbivore size class 3 (125–500 kg), Luzón et al.^1^ indicate that they are well represented by all anatomical elements (e.g., craniodental elements account for ~ 30% of the remains, while both axial and appendicular elements show frequencies > 20%). This pattern is like the one reported in VM3 for medium-to-large sized ungulates^7–10^. However, Luzón et al.^1^ indicate a bias in the disproportionate amount of posterior limb remains compared to anterior limb specimens, which in their opinion contrasts with the more balanced representation of these elements observed in VM3. Specifically, the number of forelimb bones (13.8%, 54 out of 392 bones) is about half the abundance of hindlimb bones (25.3%, 99 bones). This difference is statistically significant (χ^2^ = 16.460, *p* < 0.0001) because the sample size studied here is larger than in the former case. Moreover, Luzón et al.^1^ do not report on the presence of astragali or calcanei, highly mineralised bones well represented in the faunal assemblage of VM3^5,7,9,10^. Given that these elements are not absent from VM4 (our own dataset for VM4 includes nine calcanei and 20 astragali out of 78 autopodial bones: Table S1), their inclusion would further increase the frequency of hindlimb bones. On the other hand, they indicate the presence of 25 pelvises, which outnumber all major limb bones except the tibia (in our dataset, the number of pelvises is 16). Compared to the limb bones, the pelvis is a flat anatomical structure composed of three poorly mineralised bones, which uses to be fractured by the hyaenas for accessing its internal nutrients. As a result, it is usually recorded as fragments that preserve the acetabulum^7^. This suggests that the high number of pelvises reported by Luzón et al.^1^ probably represent pelvis fragments, which explains in part the overabundance of hindlimb elements. In VM3, *E. altidens* and *Bison* sp. are better represented by limb bones than by cranial and axial elements^8^. The living hyaenas do not cooperate to transport large portions of a carcass, which limits the individuals in what they can move^18^. Given the size of the carcasses of the adult individuals of these species, which exceeds what even a hyaena as large as *P. brevirostris* could transport, the overabundance of limb elements of horse and bison indicates the dismemberment of the carcasses and the selective transport to the denning site of the limbs due to their high marrow yields^8,10^. In the case of the horse, hindlimbs predominate over forelimbs in VM3, because the femur and tibia provide more marrow than the humerus and radius, respectively, and this resulted in the preferential transport of hindlimbs by the hyaenas to their den^8^. Therefore, the pattern of skeletal representation noted by Luzón et al.^1^ in VM4 for the size category of the horse, the species better represented in the assemblage, agrees also with the expectations from VM3.

Figures 3 and 4 show examples of the patterns of preservation of cranial and postcranial remains of ungulates in VM4 and VM3, which are strikingly similar. The data compared above on species abundances and skeletal representation suggest that the small differences outlined by Luzón et al.^1^ between both quarries result from random oscillations emerging from the small sample studied at VM4. Surprisingly, they only analyse taphonomically the fossils of large mammals of the years 2005 and 2019–2020 (1578 remains), but do not include those unearthed during the years 2013–2015 (3729 remains). Their inclusion would raise the number of specimens studied to 5338 specimens, a figure more in accordance with the information available for VM3 (the site they intend to compare their results with), which would make their analyses more robust. Moreover, it is difficult to understand why Luzón et al.^1^ do include 4219 skeletal remains in the analysis of spatial patterns with random forest algorithms, which allowed them to assign confidently most of these fossils to any of the two bone accumulations identified at VM4, but do not use such data for analysing the patterns of species abundances and skeletal representation.

### Patterns of bone weathering

**Figure 5:**
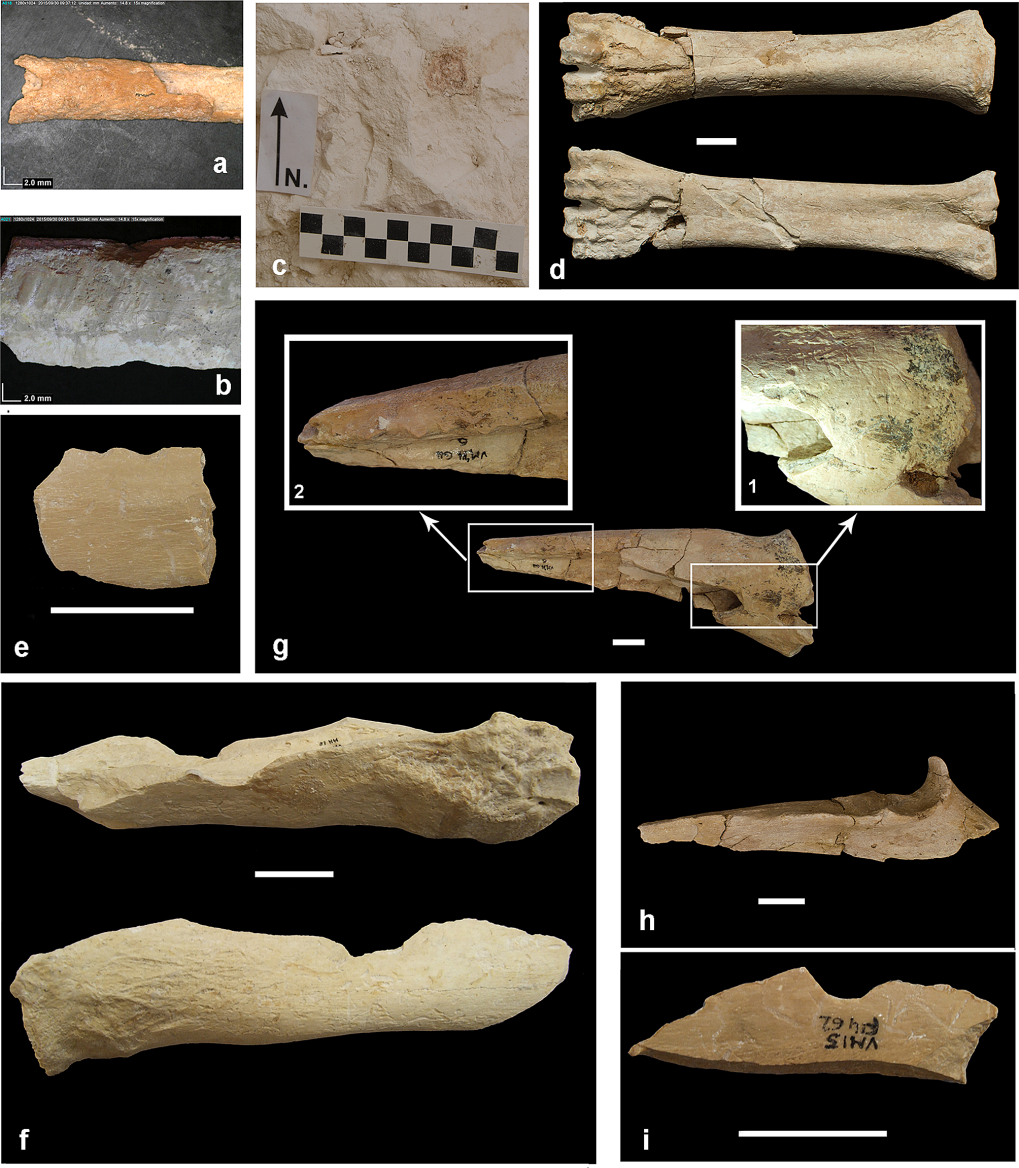
Examples of bone remains and a hyaena coprolite from the excavation quarry VM4 of Venta Micena. (**a**) Diaphysis of a digested long bone of bird. (**b**) Bone fragment showing gnaw marks made by a porcupine (*Hystrix* sp.). (**c**) Coprolite of *Pachycrocuta brevirostris.* (**d**) Third-fourth metacarpal of a large Bovini showing a different degree of bone weathering in its proximal and distal part (this bone, preserved complete, shows some diagenetic fractures orthogonal to the major axis, which resulted from sediment compaction). (**e**) Bone flake with micronotches and tooth marks. (**f**) Fragment of a radius of a large-sized ungulate (the upper view shows the presence of a double opposing notch, the lower one shows one notch, pits, and scores). (**g**) Proximal radius of megacerine deer *Praemegaceros* cf. *verticornis* fragmented and consumed by the hyaenas [the enlarged photographs show pits (1) and crenulated edges (2)]. (**h**) Ulna of hippo *Hippopotamus antiquus* consumed by the hyaenas, showing several pits. (**i**) Notch on an indeterminate bone fragment. Scale bars in (**c**-**i**) represent 2 cm.

Luzón et al.^1^ indicate that 90.8% (1461/1609) of the specimens analysed of VM4 show weathering stage (ws) 0 (bones with no sign of cracking or flaking, preserving their cortical surface intact), which indicates less than 1 year of subaerial exposure before burial^19^, while the remaining 9.2% of elements show ws 1 (cracks poorly developed and longitudinally oriented in the long bones: 0–3 years) or ws 2 (outermost concentric layers of bone showing mosaic flaking and deeper split line cracks: 2–6 years)^19^. In the case of VM3, the estimates calculated over a substantially larger sample of skeletal remains (4921 bones, updated from Ref.^9^) are the following: 75.9% ws 0, 23.4% ws 1, 6.3% ws 2, and 0.04% ws 3 (bone surface with patches of rough, homogeneously weathered compact bone where all the external, concentrically layered bone has been exfoliated: 4–15 years of exposure)^19^. Half of the bones of VM3 classified within ws 1 show shallow split line cracks due to insolation in only one side or in a part or their outer surface, while the other shows ws 0 (Fig. 5d). For this reason, they could equally well have been classified within the latter category, which would raise the frequency of elements with ws 0 to ~ 88%, a figure more in accordance with the data of Luzón et al.^1^. A similar proportion of the few remains classified in VM3 within ws 2 display ws 1 in a part or a side. If they are considered within the latter category, this would raise the percentage of bones with ws 1 to ~ 12%, a figure also closer to that of Luzón et al.^1^. In summary, these estimates suggest that the skeletal remains of VM3 show a slightly more advanced degree of weathering than those of VM4, which tentatively indicates that part of the bones preserved at VM3 were exposed during a longer time before burial. However, most bones from both quarries were buried very shortly after the death of the animals, less than one year. Moreover, the medullary cavities of the bones fractured by the hyaenas in VM3 are infilled by mud flows, but those preserved complete do not show infillings, even in the areas close to large nutrient foramina. This indicates that they were buried with the greasy periosteum intact, which suggests a subaerial exposure of few months^7^.

### Elements in anatomical connection

**Figure 6:**
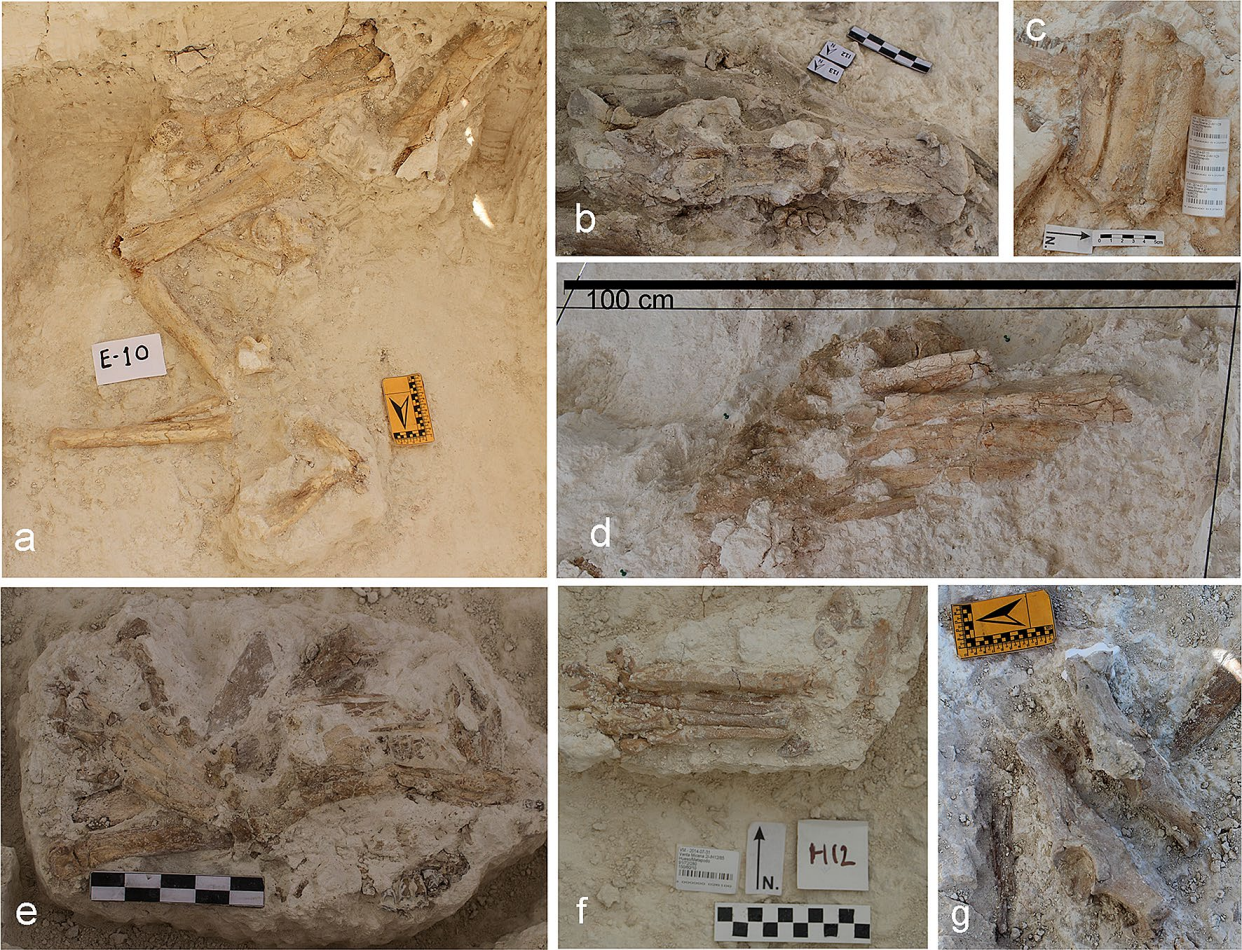
Several elements found in anatomical connection in the excavation quarry VM4 of Venta Micena. (**a**–**c**) *Stephanorhinus* aff. *hundsheimensis* [hindlimb (**a**); axis, first, and second cervical vertebrae (**b**); second, third, and fourth metacarpals (**c**)]. (**d**) Group of five dorsal vertebrae of elephant *Mammuthus meridionalis.* (**e**) Forelimb of an indeterminate felid of small size. (**f**) Hindlimb of a large-sized canid. (**g**) Two hemipelves of horse *Equus altidens.*

According to Luzón et al.^1^, many bones of VM4 are anatomically connected (e.g., a humerus-radius and a femur-tibia, fibula, and talus of rhino *S. hundsheimensis,* both sets located some meters distant; a group of seven dorsal vertebrae of *M. meridionalis;* an almost complete forelimb and two complete hindlimbs of *L. lycaonoides,* also separated by a short distance; and two hemipelvises of *E. altidens*). During the field excavation seasons of 2013–2015, which they do not study, other elements of the same rhino skeleton (e.g., a humerus-radius and a femur-tibia, fibula, talus, and three metapodials as well as a cervical section including the axis and two cervical vertebrae; Fig. 6a–c) were found. In addition, some vertebrae of *M. meridionalis* (Fig. 6d), a complete forelimb of a small-sized felid (Fig. 6e), two complete hindlimbs of a large canid, probably *L. lycaonoides* (Fig. 6f), a skull of *P. brevirostris,* and two hemipelvises of *E. altidens* (Fig. 6g) were unearthed. Surprisingly, these fossils are not cited by Luzón et al.^1^. Although there are also elements of VM3 in anatomical connection (e.g., several skulls showing the mandible articulated with the cranium, two hemipelvises of a juvenile horse, some groups of lumbar vertebrae and several groups of autopodial elements)^7,9,20^, their frequency is lower than in VM4. However, many bones of VM3 are disarticulated but spatially associated to others with which they were anatomically connected in origin^7,9^ (Fig. S3). Again, this difference suggests that the bones preserved at VM3 were exposed during a longer time before their burial, which resulted in more weathering and a higher degree of modification by the hyaenas. Most probably, the reason was that the breeding season in the denning site of *P. brevirostris* in VM3 prolonged a little longer than in VM4 before the rise of the water level of the lake covered the area and capped with limestone the bone assemblage.

### Patterns of skeletal completeness

Luzón et al.^1^ affirm that the skeletal remains of VM4 show a moderate degree of fragmentation. This claim is contradicted by data provided in their Table 2: ~ 36% of the remains measure < 3 cm and > 55% of limb bones show green fractures, which evidence their breakage in fresh state by the hyaenas. In VM3, a substantial proportion of the remains are also small shaft fragments and one third of them measure < 3 cm, like in VM4. Isolated epiphyses and diaphyseal bone shafts are very abundant and outnumber the bones preserved complete in VM3^9,10^: 80.5% (963/1196) of the major limb bones of equids, bovids, and cervids are represented by isolated bone portions that show green fractures (mostly spiral or longitudinal fractures) and only 19.5% are preserved complete^7,9^. These fractures were produced by the hyaenas during the biostratinomic stage, when the bones were still fresh and retained their marrow contents. Green fractures predominate over diagenetic fractures resulting from sediment compaction, which are found in 22.5% of the bones of VM3^9^. The latter tend to be orthogonally oriented to the major axis in the long bones and are delimited in all cases by both bone portions (i.e., there is no fracture defining the end of one specimen), which shows that the assemblage is not reworked^7^.

### Bone transport

There is no evidence of transport by water currents in VM3, because the bones: (i) show a random pattern in their spatial orientation; (ii) have no rounded or polished edges; (iii) show no traces of abrasion from rolling or similar movements within a current; and (iv) detritics are nearly absent from the sediment, which composition is 90–98% pure micritic limestone precipitated in water ponds emplaced on a caliche palaeosoil of diagenetic origin^7,9^. In the case of VM4, Luzón et al.^1^ also indicate the absence of bones with rounded surfaces, which helps them to discard fluvial currents as responsible of the accumulation of skeletal remains. However, they affirm that ~ 40% of specimens show evidence of hydraulic abrasion. Given that it is difficult to explain the presence of bones that were abraded but not rounded, they suggest the following ad hoc hypothesis: while the currents were weak for displacing the remains, abrasion resulted from circulating waters that moved the sediment over the bones, which originated their abrasion^1^. However, detritics are absent from the whole VM stratum^7^ and it is difficult to explain the finding at VM4 of microvertebrate remains, which should have been transported by water currents of low energy. Luzón et al.^1^ indicate that the fossil remains of the two levels of accumulation described in VM4 show patterns of preferential orientations towards the NE. This is also difficult to explain, because they discard the role of hydraulic currents in the transport and deposition of bones. Concerning bone dip, only 4% of the remains show azimuth values over 45°, while 79% of bones are found relatively flat along the topography of the excavated surface^1^. As a result, they suggest gravity as the likely cause for the observed patterns of bone inclination at VM4^1^, a hypothesis previously suggested for VM3^7^. In the case of VM3, rose diagrams show no preferential patterns of alignments in the orientation of the skeletal remains, while dip angles greater than 30° are poorly represented and bones showing a vertical or subvertical inclination are very scarce^9^.

### Carnivore tooth marks

Luzón et al.^1^ indicate that carnivore alterations were only observed in 4.5% of those bones with a well-preserved cortical surface and that only three bones showed 3–6 tooth marks. The frequency of tooth-marked remains in the larger sample of VM4 analysed here is slightly higher, 5.5% (177/3227) (Table S3). The marks include scores, pits, notches, crenulated edges, and furrows made by the hyaenas (Table S4, Fig. 5). Of these marks, ~ 60% appear in limb bone shafts. In addition, one bone shows marks made by a porcupine (Fig. 5b). In the case of VM3, 29.4% (1555/5288) of the remains analysed show carnivore tooth marks^9^. These bones belong to all ungulate species identified in the site and there are also some tooth-marked bones of *Pachycrocuta,* both of adult and non-adult individuals. Many cranial fragments and most limb bones of VM3 show striations and biting marks, the preserved epiphyses have furrows and punctures, and the diaphyses, as well as the skull bones, show scoring and pitting. Pits, scores, and notches are the marks most frequently recorded, although crenulated edges and furrows are also abundant^7,9^. The proportion of tooth-marked bones in VM3 was in all probability even higher, as many limb bones of the assemblage that do not preserve tooth marks show fracture patterns that evidence that they were broken in fresh state by the hyaenas^9^, and this also applies to VM4. A significant part of the tooth marks preserved in VM3 were probably produced by juvenile hyaenas, which deciduous teeth are more cutting than the permanent premolars of adults, which are progressively blunted by bone cracking, and this results in inconspicuous tooth marks^7^. However, a minor implication of some small or medium-sized carnivore like the wolf *C. orcensis*^21^ cannot be discarded. Among modern hyaena dens, the frequency of tooth marked bones is highly variable^17^: 29.0–53.5% in spotted hyaenas, 22.1–100% in brown hyaenas, and 6.0–56.2% in striped hyaenas (the lower limit for this species is contentious, because it corresponds to a den where the bones are highly weathered). These frequencies are higher than in VM3 and VM4, but many fossil bones of the site are pending of restoration and evidence of gnawing by juvenile hyenas is usually very subtle.

The higher proportion of tooth-marked bones in VM3 agrees with a longer exposure of the skeletal remains than in VM4, which would explain why they were exploited more thoroughly by the hyaenas. As discussed earlier, this is also suggested by the more advanced degree of bone weathering and by the lower frequency of articulated elements. According to Luzón et al.^1^, bones with salivary and gastric alterations are absent in VM4, but they are recorded in VM3 at very low frequencies (0.34% and 0.15% of 5288 specimens analysed, respectively)^9^. This suggests that their absence from VM4 results from the small sample of remains studied. In fact, our analysis of the specimens from the excavation seasons not studied by Luzón et al.^1^ showed the presence of two bones with evidence of digestion (0.06% of the specimens analysed; Fig. 5a) and two others with salivary alterations resulting from licking (Table S4). In the study of 24 hyaena dens cited above^17^, evidence of acid or gastric edging of bones was detected in only eleven skeletal remains (six from spotted hyaena, four from brown hyaena and one from striped hyaena assemblages). These low numbers are not surprising, because neither striped hyaenas nor brown hyaenas regurgitate bones.

Finally, Luzón et al.^1^ indicate that hyaena coprolites are absent in VM4, but a small coprolite was unearthed during the excavation of 2014 (Fig. 5c). The hyaena den of VM3 also preserves some coprolites represented by isolated pellets with diameters of 3–6 cm^7^.

### VM4 and VM3: coeval or successive bone accumulations?

According to Luzón et al.^1^, VM4 is in the context of a series of short-time events (they identify two of them in VM4, while only one was recognized in VM3^7^) followed by rapid sedimentation, as indicated by the low degree of bone weathering, the low frequency of tooth-marked bones, and the presence of skeletal remains anatomically connected. In the case of VM3, the more advanced weathering, the higher frequency of bones with bite marks and the lower proportion of elements in anatomical connection suggest that the deposition of limestone that capped the bone assemblage after the rising of the water table of the Baza palaeolake was delayed compared to VM4.

The bone assemblage accumulated in the hyaena den of VM4 is positioned ~ 350 m distant from VM3 (Fig. 1f). This distance is very short for considering the possibility of two neighbouring hyaena clans: in Serengeti, where spotted hyaenas engage in prolonged clashes with neighbouring clans, the radius of the permanent territory defended by a clan around the communal den fluctuates between 2.6 and 5.7 km^22^. This suggests that VM4 and VM3 were not coeval but correspond to separate events of accumulation during different years. More specifically, a study of a spotted hyaena clan in Masai Mara showed that the hyaenas used 57 different sites for communal denning during a period of ten years, with an average distance between the dens used consecutively of 1.5 ± 0.1 km^23^. The distance between VM4 and VM3 is five times shorter than the one expected when the adult spotted hyaenas move their cubs to a new denning site, which suggests that VM4 and VM3 do not represent a residential move of the same hyaena clan. Therefore, the most parsimonious interpretation is that the bone assemblages preserved at both denning sites were accumulated by the hyaenas during the dry seasons of different years in the emerged plain that surrounded the lake surface covered by permanent waters (Fig. 1f). During the interval of time in which the accumulation of the bone assemblage of VM3 took place, the rising of the water table in the rainy season probably occurred some months later than during the years that correspond to the two accumulations of bone remains detected in VM4^1^. Following this interpretation, the remains accumulated at the denning site of VM4 were capped with limestone (which protected them from weathering) somewhat earlier than those of VM3, which explains the minor taphonomic differences between both sites cited above. This interpretation also helps to clarify why the whole VM stratum, which outcrops along ~ 2.5 km, seems to be littered with fossils of large mammals, as it is difficult to conceive a “megadenning site” of *P. brevirostris* that extended over several squared kilometres. In our model, the VM stratum would represent successive deposits of micritic limestones in the plain that surrounded the Baza palaeolake during several lowstand-highstand cycles, each corresponding to the dry and rainy seasons of one year (Fig. S2d). Each year, the hyena clans that inhabited the Baza Basin would randomly select their denning sites on this plain. After enough years, the surface seasonally submerged of the plain that surrounded the lake would be almost entirely covered by fossils of large mammals, which were preserved in the micritic limestones of the VM stratum.

## Concluding remarks

Taphonomic analysis of the remains of large mammals preserved at VM4 shows that this bone assemblage is very similar to the one preserved at VM3, the main excavation quarry of Venta Micena, from which many thousands of fossils were unearthed during the last decades. Contingency tables show that the only significant differences between both sites are the frequencies of skeletal remains of megaherbivores, slightly overrepresented at VM4, and of horse *E. altidens,* a species more abundant in VM3. These variations in prey abundance are not unexpected in natural ecosystems according to survey data from different years. Many differences between VM4 and VM3 reported by Luzón et al.^1^, particularly those related to the abundance of juvenile individuals, result from poor sampling, which is reflected in low NISP and MNI estimates for most species. Our study, based on a larger dataset, shows no major differences in the taphonomic signatures of VM4 and VM3 except for a somewhat longer time of exposure at VM3, which resulted in a more in-depth consumption by the hyaenas of the bones accumulated. Therefore, the results obtained in this study suggest that the bone assemblages of VM4 and VM3 were produced in non-coeval denning areas of *P. brevirostris* in the plain that surrounded the Baza palaeolake.

## Materials and methods

In this study, we analysed 8831 vertebrate fossil remains from VM3 unearthed during the field excavation seasons performed between the years 1982 and 2005. Most of these specimens were taphonomically analysed by M. P. Espigares^9^. The materials from VM4 analysed here consist of 3961 vertebrate remains (mostly large mammals) recovered during systematic excavations in the years 2005 and 2013–2015.

Anatomical and taxonomic data were determined using atlases of comparative anatomy^24–27^ and palaeontological publications on the Orce sites. Species of large mammals (mean mass estimates from Refs.^6,14^) were distributed among size categories following Refs.^28,29^: small size (S), < 23 kg; medium-to-small size (SM): 23–114 kg, medium size (M): 114–227 kg, medium-to-large-size (ML): 227–340 kg; large size (L): 340–907 kg; very large size (VL): > 2721 kg (size classes 5 and 6 from Refs.^28,29^ are grouped in this study). Elements that do not preserve taxonomically diagnostic features were classified to order, infraorder, family, or tribe level, and were then assigned to a size category.

The faunal assemblages of VM3 and VM4 were taphonomically analysed following the standard methodology^30–32^. Numbers of identified specimens (NISP), minimum numbers of elements (MNE) and minimum numbers of individuals (MNI) were calculated for all taxa. Four age groups were established for the specimens: immature individuals, subdivided in calves and juveniles, and adults, classified as adults sensu strictum (i.e., yearlings and prime adults) and past-prime adults (i.e., senile individuals). Criteria for estimating age at death included patterns of tooth replacement and degree of tooth wearing for deciduous and permanent teeth, as well as degree of epiphyseal fusion for limb bones.

Bone cortical surfaces were analysed with a stereoscopic binocular microscope (Olympus SZ 11) and a digital microscope (DINO-LITE Model AM4115TL). In the case of VM4, surface modification was analysed in only a part of the assemblage, because many bones are badly conserved and need restoration. Carnivoran activity was identified based on Refs.^33–38^. Most tooth marks identified were pits, notches, and scores; furrowing and crenulated edges were present but in lower percentages. Bone breakage patterns were classified according to Ref.^39^. Weathering and other bone surface modifications were identified and described following Refs.^19,31,40,41^.

The abundance of each species of large mammals identified in VM4 and VM3 was tested statistically using two contingency tables, one for NISP values and another for MNI estimates. In the case of VM4, data provided by Luzón et al.^1^ were used. However, given that their study only analysed the fossils from the excavation seasons of the years 2005 (245 skeletal elements) and 2019–2020 (1364 remains), we also included in this comparison unpublished data analysed by M.P. Espigares on 3974 fossils unearthed during the years 2005 and 2013–2015 (see Tables S1, S2). Data for VM3 were updated from Ref.^9^. The limitations posed on this analysis by the low sample sizes reported by Luzón et al.^1^ for most taxa in VM4 made necessary to group several species according to taxonomic affinities (e.g., large bovids, caprines, and other carnivores apart from *P. brevirostris*) or size categories (e.g., megaherbivores).

Each contingency table has *r* rows (species or group of species) and *c* columns (their raw abundances in the two datasets for VM4 and in VM3). The statistic for testing against independence between species abundances and assemblages is: χ^2^ = Σ_i=1_^r^ Σ_j=1_^c^ (O_ij_ − E_ij_)^2^/E_ij_, where O_ij_ is the observed frequency of species *i* in assemblage *j* for the *ij-*th cell (i.e., n_ij_) and E_ij_ represents the expected frequency for this cell under the null hypothesis of independence (i.e., a random, homogeneous distribution of species among the assemblages). The latter is computed as: E_ij_ = (Σ_i=1_^r^ n_i._·Σ_j=1_^c^ n_.j_)/Σ_i=1_^r^·Σ_j=1_^c^ n_ij_, where n_i._ and n_.j_ are the total number of cases that show the *i-*th and *j-*th attributes [partial sums for rows (species) and columns (assemblages) in the table, respectively]. When the null hypothesis holds, χ^2^ is approximately distributed as a chi-square variable with (r − 1) (c − 1) degrees of freedom.

The individual cells of the contingency tables were also analysed independently with the method of adjusted residuals^10^. This allows the determination of which species or groups of species (rows) are significantly over-represented in each assemblage (columns). Let e_ij_ = (O_ij_ − E_ij_)/E_ij_^1/2^. The mean of this variable equals zero and its variance is v_ij_ = (1 − n_i._/n)·(1 − n_.j_/n). The adjusted residuals are d_ij_ = e_ij_/v_ij_^1/2^, and they result from standardization (i.e., z-score normalization) of e_ij_ values. Adjusted residuals are approximately normally distributed [N(0,1)] when there is no association between the rows and columns of the contingency table. However, a situation of dependency generates residuals that are higher in absolute value than the standard normal deviate for a specific level of confidence (e.g., 1.96 for *p* < 0.05). For this reason, when the absolute value of the adjusted residual (*d*_*ij*_) for a given cell is higher than this deviate, the null hypothesis of independence is rejected for this cell (a positive value indicates an over-representation of the *i-*th species in the *j-*th assemblage compared to the expectations from a random distribution of species among assemblages, while a negative one points to an under-representation).

A randomization test for contingency tables was also used to compare species abundances in the assemblages of VM4 and VM3 without the need of grouping the species with low frequencies in larger categories^42^. It is worth noting that MNI estimates are more adequate for describing the relative frequencies of species in those assemblages that show a high degree of fragmentation^43^. Given that most bones of VM3 and VM4 were subject to ravaging by the hyaenas, the use of MNI counts seems the best choice for comparing both assemblages. According to data from Luzón et al.^1^, sample sizes were fixed for VM4 in 390 (NISP values) and 52 (MNI counts). The frequencies considered for VM3 were 6331 and 339, respectively (these numbers refer to those remains identified taxonomically: see Table 1). This allowed to generate an empirical distribution of the χ^2^ statistic by simulating a set of random samples (n = 10^4^) according to the marginal frequencies of each species.

## Supplementary Information

Supplementary Information.

## Data Availability

The datasets generated during and/or analysed during the current study are available from the corresponding author on reasonable request. Most of these data are included in the Supplementary Information.
