# Supplementary material for: Déjà vu: a reappraisal of the taphonomy of quarry VM4 of the Early Pleistocene site of Venta Micena (Baza Basin, SE Spain)
Source: Sci Rep. 2022 Jan 13;12:705. doi: 10.1038/s41598-021-04725-3 (PMC8758678; doi:10.1038/s41598-021-04725-3)
Supplement: Supplementary file 1 — Supplementary Information. [file 41598_2021_4725_MOESM1_ESM.pdf]

Supplementary Information of "Déjà vu: A reappraisal of the taphonomy of quarry VM4 of the Early Pleistocene site of Venta Micena (Baza Basin, SE Spain)"

Paul Palmqvist, M. Patrocinio Espigares, Juan A. Pérez-Claros, Borja Figueirido, Antonio Guerra-Merchán, Sergio Ros-Montoya, Guillermo Rodríguez-Gómez, José Manuel García-Aguilar, Alejandro Granados, Bienvenido Martínez-Navarro

Geology and sedimentological context of Venta Micena

Venta Micena-4 (Luzón et al. 2021) lies in the NE sector of the Baza Basin (Guadix-Baza Depression, Province of Grenade, SE Spain), in the vicinity of the town of Orce (Fig. S1). This inland basin extends over an area of ~4,000 km^2 and is surrounded by the highest reliefs of the Alpine-Betic orogen, which rise an altitude of ~3,000 m. The basin preserves a sedimentary record of high stratigraphic completeness of the continental Plio-Pleistocene, composed of lacustrine and fluvial deposits, including limestones, marls, shales, sands, and conglomerates, as well as dark clays and silexites originated from hot springs, which are associated to the main archaeological and palaeontological sites of the basin (García-Aguilar & Palmqvist, 2011; García-Aguilar et al. 2014, 2015). These deposits show a complex sedimentary architecture, which results from active tectonics and orbitally induced climatic cycles (García-Aguilar et al. 2013). The basin was connected to the Mediterranean Sea by the Almanzora Corridor, which closure between the end of the Tortonian and the latest Messinian–Zanclean led to a continental regime (Guerra-Merchán 1990, 1993; Soria et al. 1999; Hüsing et al. 2010). From these times onwards, it was subject to isostatic uplifting, with an average uplift rate of ~200 m/Ma, as estimated from Late Neogene coastal marine conglomerates and coral reefs (Braga et al. 2003). As a result, the glacis surface of the basin (i.e., the uppermost horizontal infilling level) stands now ~1,000 m on average above sea level.

During most of the Plio-Pleistocene, the Guadix-Baza Depression developed a network of endorheic drainage, being subject to: (i) intense tectonic subsidence relative to the surrounding mountains, which allowed the accumulation of a thick (>400 m) and relatively continuous record of continental sediments; and (ii) hydrothermal activity (Figs. S1B–C), which provided a mild and productive environment for the terrestrial fauna (García-Aguilar et al. 2014, 2015). Hot springs were a major determinant in the establishment of a diverse fauna of large mammals, which remains were preserved in many localities across the basin (e.g., Viseras et al. 2006; Arribas & Palmqvist, 1998; Arribas et al. 2009; Palmqvist et al. 2005; Ros-Montoya et al. 2017; Maldonado-Garrido et al. 2017; Martínez-Navarro et al. 2018). The Orce area, a satellite basin in the NE sector of the sedimentary depression with an extent of ~170 km^2, preserves a worldwide unique fossil record: for example, >24,000 skeletal remains of large mammals have been unearthed from a surface of ~400 m^2 excavated in the late Early Pleistocene (Calabrian, Late Villafranchian) site of Venta Micena during a number of excavation seasons unevenly distributed across the last decades (the fossils come from several excavation quarries, including VM2, VM3, and VM4, but most of them are from VM3; Fig. S2). This represents a mean density of fossils of more than 60/m^2 (Martínez-Navarro 1991; Palmqvist et al. 1996; Arribas & Palmqvist 1998; Palmqvist & Arribas 2001; Espigares 2010; Palmqvist et al. 2011; Martínez-Navarro et al. 2018). Although this density is not homogeneously recorded across the 80-120 cm thick Venta Micena stratum, which outcrops over ~2.5 km (Anadón et al. 1987), it suggests that tens of millions of fossils were preserved in the micritic limestones of this lithological unit (Arribas & Palmqvist 1998).

From a stratigraphical and sedimentological point of view, several issues raised by Luzón et al. (2021) are subject to debate. First, they indicate that "the Orce region was subject to the lowstand–highstand dynamics of a large saline lake that dominated the basin", but they do not clarify the temporal extent of this lake. This represents an oversimplification of the sedimentary dynamics of the Baza Basin. The palaeolake of this basin extended over ~1,125 km^2 and was part of a more complex sedimentary system that included alluvial, fluvial, carbonate lacustrine, detrital lacustrine, and evaporitic lacustrine facies. As a result of tectonic factors, the deposits of these facies varied in both time and space. In the case of the lacustrine environments, the deposits relied also on the effects of allocyclic factors on climate and precipitations, including oscillation cycles within the Milankovitch band (García-Aguilar & Palmqvist 2011). Gibert et al. (2007) and Medina-Cascales et al. (2020) consider that the lithological unit that characterizes the large saline lake was deposited during the whole Pliocene and part of the Pleistocene, showing a rather continuous sedimentation through time with lateral changes of facies to other marginal units. In contrast, other researchers (e.g., Vera et al. 1984; Soria et al. 1987; Garcés et al. 1997; García-Aguilar & Martín 2000; García-Aguilar & Palmqvist 2011; García-Aguilar et al. 2014) indicate a discordance between two tectono-sedimentary units. The unit situated below the discordance is represented by alluvial conglomerates in the northern edge of the basin. Laterally, these facies change to lacustrine marls and limestones, with local appearances of travertines. The sites of Botardo and Galera 1C (Martín Suarez 1988; Agustí et al. 1997; Piñero & Agustì 2020), as well as others not published by the moment, indicate a latest Miocene–Early Pliocene age for this unit (i.e., top of biozone MN-13, MN-14, and MN-15). The unit is also recognized in the southern edge of the basin (Baza sector), in the sites of Colorado (1 and 2), Aljibe (1, 2, and 3), Baza-1, and Cómodo, which allows to assign them the same age (Guerra-Merchán 1993; Ros-Montoya et al. 2017). During this stage, climate was warm and moist, which evidences an environment typically subtropical or a rainy tropical climate (Foucault & Mélières 2000; Haywood & Valdés 2004). This climate would not favour an evaporitic sedimentation. Towards the interior of the basin, the unit placed above the discordance is characterized by detrital–evaporitic sedimentation, showing an alternation of sandstones and gypsum, or marls and gypsum. Towards the edge of the basin, these lithologies change to conglomerates, sands and lutites, which were deposited in fluvial or fluvio-lacustrine environments. This stage shows intense synsedimentary tectonic activity, which favoured a high subsidence. As a result, the central sector of the basin achieves a sediment thickness of at least 300 m (Vera 1970; García-Aguilar & Martín 2000; García-Aguilar & Palmqvist 2011). Climate was semiarid, with cyclic oscillations in the Bond band of frequencies (Hagelberg et al. 1994; Bond et al. 1997; García-Aguilar et al. 2013). This resulted in alternating periods of flooding and drying of the outer parts of the lake over hundreds to thousands of years (these cycles are represented by deposits of marls/sands and gypsum, respectively). The gypsum disappears towards the upper part of the unit, being substituted by a predominance of conglomerates and sands, which evidences a context of progradation of detritic sediments towards the interior of the basin. Biostratigraphic and paleomagnetic data (Agustí et al. 1997; Garcés et al. 1997) indicate an age range for this unit between the upper part of the Late Pliocene (MN16) and the lower part of the Early Pleistocene (MmQ1). In contrast, the lithological unit in which are placed the Early Pleistocene site of Venta Micena and two other sites of Orce that are slightly younger, Barranco León and Fuente Nueva-3, is included in the MmQ2 biozone of the Matuyama Chron, between Jaramillo and Olduvai (Scott et al. 2007), as acknowledged by Luzón et al. (2021). This means that Venta Micena is younger than the evaporitic unit cited before. For this reason, to affirm that the lake in which the limestones of Venta Micena were deposited was isolated from the large saline lake that covered most of the Baza Basin (Luzón et al. 2021) is incorrect, because this saline lake did not exist by these times.

Second, Luzón et al. (2021) also affirm that "when the coastline of the lakes recedes, numerous fresh-water lagoons could arise". This statement is also wrong according to most sedimentary models linked to lake environments (Anadón-Monzón 1984; Freytet 1975; Murphy & Wilkinson 1980): during a lowstand cycle of lake retreat, the lagoons disconnected from the main water body cannot be considered as "freshwater" ponds, because they show an oligosaline character due to water evaporation and emersion. Sedimentologically, this is evidenced in the shallowing upward lake sequences of VM (Fig. S2d). Moreover, the sediments of Barranco León preserve several mollusc species (e.g., gastropods *Bithynia tentaculata, Hydrobia* aff. *acuta, Melanoides tuberculata,* and *Gyraulus* cf. *laevis,* bivalve *Pisidium casertanum*) that indicate an euryhaline environment (Albesa & Robles 2020). The limits of tolerance to salinity of these species offer clues on the lake waters: although *B. tentaculate, H. acuta* and *M. tuberculata* can live in waters with elevated salinities (12–30‰), their optimum is lower (e.g., 0.2–3‰ in *M. tuberculata*). In addition, *P. casertanum* and *G. laevis* tolerate salinities of only up to 3–5‰ (Albesa & Robles 2020). This indicates a thermophile lacustrine environment with alternating phases of salinity, from moderately oligosaline waters in the main body of the lake to brackish conditions in the surrounding swampy areas. Such environmental inference agrees with: (i) the presence in the sediments of microcrystalline gypsum originated by neoformation (García-Aguilar et al. 2014); (ii) the high $\delta^{15}N$ values measured in the bone collagen of the large-sized hippo *Hippopotamus antiquus,* a species more dependent of the lacustrine environments than the extant *H. amphibius,* as it fed predominantly on the aquatic macrophytes that grew in the oligosaline waters of the lakes instead of consuming terrestrial grasses, as do the living hippos (Palmqvist et al. 2003, 2008a, 2008b; García-Aguilar et al. 2014, 2015); and (iii) the identification in VM4 of skeletal remains of an aquatic bird preliminarily attributed to a common Shelduck (*Tadorna tadorna*). This waterfowl dwells in coastal mudflats and lagoons, estuaries, and riverine environments of Europe, generally occurring in salt or brackish water, where it feeds mostly on aquatic invertebrates, with predilection for saltwater snail *H. acuta* (Carboneras & Kirwan 2018), a species that can survive at salinities of up to 39‰ (Britton 1985). In the case of Venta Micena, the finding of the ostracodes *Ilyocypris bradyi* and *I. gibba* provides additional information on the paleoenvironment: *I. bradyi* is typical of high energy streams with abundant underwater vegetation, while *I. gibba* lives in shallow lake borders with warm and fresh to oligosaline waters, a sandy substrate and lush vegetation (Martínez-García et al. 2015, 2017).

On the bone collecting agent at VM4

Luzón et al. (2021) acknowledge that the presence of tooth marks, furrowing and notches make undeniable the role played by carnivores in the formation of the bone assemblage preserved at VM4. However, they contradict themselves by stating that while the impact of carnivores on VM4 is notable, carnivore activity in general can be considered of low intensity given the appearance of skeletal elements in anatomical connection, the low frequency of tooth-marked bones and of bones with furrowing, as well as by the absence of digested bones and salivary alterations, which are also scarcely represented at VM3. In their opinion, these observations contrast with the taphonomic features of other bone assemblages conclusively collected by the giant, short-faced hyaena *Pachycrocuta brevirostris,* such as Vallparadís, Zhoukoudian, VM3, and Fonelas P-1. For this reason, they affirm that VM4 should not be considered as a hyaena denning site, as described in the case of VM3. Although not explicitly acknowledged, this is the one of the cores of their paper, because they suggest that *P. brevirostris* was not the main accumulating agent at VM4, which would imply that other carnivores were responsible of collecting and modifying the bone assemblage preserved at this quarry. In fact, Luzón et al. (2021) conclude that future research at VM4 will attempt at discerning the precise carnivores involved in the site

formation process, identifying the “presence of a single or multiple predator types”. Therefore, it is worth considering here which carnivores other than the hyaenas could be responsible of generating the bone accumulation of VM4 and, eventually, the one of VM3, as Luzón et al. (2021) even suggest that their hypothesis that *P. brevirostris* was not the main accumulating agent at VM4 also provides an interesting point of debate for interpreting VM3.

Apart from *P. brevirostris,* four medium-to-large sized hypercarnivores are present at VM4: two machairodonts, the scimitar-tooth *Homotherium latidens* and the dirk-tooth *Megantereon whitei,* the jaguar *Panthera* cf. *gombaszoegensis,* and the wild dog *Lycaon lycaonoides.* In addition to these primary predators, there are several small-to-medium sized carnivores such as *Lynx* cf. *pardinus,* canids *Canis orcensis* and *Vulpes alopecoides,* and the badger *Meles meles,* the latter only identified in VM3. Given the behaviour of their living analogues, it is unreasonable to consider any of these small carnivores as an effective accumulating agent of skeletal remains of large-bodied ungulates. Similarly, the bear *Ursus etruscus* can be also discarded due to its omnivorous diet, evidenced by stable isotopes, tooth-microwear and morphometric analyses (Palmqvist et al. 2008b; Medin et al. 2017, 2019). In the case of the pantherine felid *P. gombaszoegensis,* Luzón et al. (2021) include this species in their Table 1. However, they indicate in their supplementary information that this species has been only recorded at VM3. There is another incongruency concerning *L. lycaonoides:* Luzón et al. (2021) calculate in their Table 1 a NISP = 8 for this wild dog, although they indicate in the text that a couple of hindlegs (which include many more bones) were unearthed from VM4.

The predatory guild of VM4 was dominated by two species of sabre-tooth cats, *H. latidens* and *M. whitei* (Martínez-Navarro & Palmqvist 1995, 1996; Arribas & Palmqvist 1999). Although Luzón et al. (2021) do not seem to consider neither of them as a possible accumulating agent, they suggest that these predators exploited in depth the carcasses of their prey. *Megantereon* is a stoutly built dirk-tooth machairodont with an estimated body mass of 100–110 kg (Martínez-Navarro & Palmqvist 1996; Christiansen & Adolfssen 2007) (i.e., like a small lioness or a large male jaguar). The low value of the brachial index (i.e., ratio of humerus length to radius length), ~80%, and the short metapodials suggest at first glance an ambush predator that hunted in mixed and closed habitats (Palmqvist et al. 2003). The forelimbs are powerful, with a large dewclaw, and the upper canines are extremely long, sharp, and laterally compressed (Arribas & Palmqvist 1999; Palmqvist et al. 2007; Christiansen & Adolfssen 2007). In contrast, *Homotherium* is a scimitar-tooth cat similar in size to a modern lion (145–220 kg; Anyonge 1993) with forelimbs more gracile and elongated than in the typically hyper-robust dirk-tooths like *Megantereon* or *Smilodon,* which provided considerable leverage (Anyonge 1996a). Given that machairodonts have shorter forelimbs than pantherine cats of similar size (Palmqvist et al. 2003), the higher value of the brachial index in *Homotherium* (~90%) points also to increased cursoriality. The presence of partially retractable claws in *Homotherium,* a condition only found in the cheetah among modern felids, is inferred from reduced asymmetry of the middle phalanges (Antón et al. 2005) and from small attachment scars on the metacarpals of digits II–V for the muscles used by felids to retract the claws (Rawn-Schatzinger 1992). This condition indicates less prey-grappling capability than in other sabre-tooths, which suggests moderately increased cursoriality in open habitat (Martin et al. 2000) and indicates an adaptive trade-off between improved traction for pursuing prey and reduced grappling ability (Antón 2013). Compared to the pantherine felids, the greater tuberosity of the humeral head and the narrower distal radius of *Homotherium* also suggest adaptations for cursoriality, although it must be noted that the lumbar spine of this scimitar-tooth cat is shorter and stiffer than in the cheetah and that the tail of *Homotherium* is short (Antón et al. 2005), a condition also suggested by the strong posterior tapering of the sacrum in *Megantereon* (Christiansen & Adolfssen 2007).

Moreover, the cheetah has long hindlimbs relative to its forelimbs, but this proportion is reversed in *Homotherium,* which results in a sloping back, like in a hyaena (Rawn-Schatzinger 1992). These features, well studied in the North American *H. serum,* suggest that while the cheetah can accelerate quickly to develop short bursts of maximum speed, *Homotherium* was better equipped to run at more moderate speeds but over longer distances, as in the case of hyaenas (Antón et al. 2005). The reduction of claw retractability limited grappling efficiency in the forepaws, which suggests that *Homotherium* may have required of group action for bringing down large prey. This would make it a unique cursor, flesh-specialized predator of open habitat, and potentially social (De Santis et al. 2021). In fact, the possibility of sociality has been recently confirmed with nuclear genome and exome analysis of the scimitar-toothed cat (Barnett et al. 2020). Moreover, compared with dirk-toothed machairodonts like *Megantereon* or *Smilodon,* the lack of retractable claws in *Homotherium* relates with a poorly equipped skull for delivering a unique, fatal stabbing bite to its prey (Figueirido et al. 2018). *Homotherium* has a comparatively large brain with an enlargement of the optic centre, a condition also found in the cheetah (Rawn-Schatzinger 1992). In contrast, *Megantereon* shows a comparatively smaller brain with olfactory lobes well developed, as expected in an ambush predator (Arribas & Palmqvist 1998). Interestingly, a recent comparative genomic analysis of *H. latidens* has found signatures of positive selection in several genes involved in vision, cognitive function, and energy consumption, which are putatively consistent with diurnal activity, well-developed social behaviour, and cursorial hunting (Barnett et al. 2020).

Sabre-tooths have no truly living analogues and dominated the mammalian carnivore guild during most of the Cenozoic, filling the niche now occupied by the large pantherine felids (Van Valkenburgh 2001, 2007). Their large, laterally compressed upper canines represent an adaptation for killing quickly and efficiently large prey (including megafauna) with deep wounds onto the prey throat rather than using the prolonged suffocating throat bite typical of the extant felines (Gonyea 1976; Akersten 1985; Anyonge 1996a; Antón et al. 2004; McHenry et al. 2007; Christiansen 2008; Salesa et al. 2010; Meachen-Samuels & Van Valkenburgh 2010; Andersson et al. 2011; Meachen-Samuels 2012; Martín-Serra et al. 2017). However, canine length posed a biomechanical constraint on mandibular gape for delivering the killing bite, which involved the reorganization of the temporalis to avoid the over-stretching of muscle fibres during wide gaping (Fig. S5). This was achieved by several changes in the craniodental anatomy of sabre-tooths compared to the pantherine felids, as discussed below. Skull reorganization in sabre-tooths resulted in a narrowing of the temporalis fibres and their more perpendicular orientation to the tooth row (Fig. S5A), which allowed to increase jaw gape up to 180º while retaining a degree of muscle stretch like that of pantherine felids (Fig. S5C). However, this also implied that the point of maximum bite force exerted at the carnassial was positioned more backwardly, which led to a reduction of the post-canine dentition not related to the slicing function of the carnassial. This reduction is particularly evident in the African sabre-tooth *M. whitei* (Martínez-Navarro & Palmqvist 1995, 1996; Palmqvist et al. 2007; Martínez-Navarro et al. 2014), as discussed below. For this reason, although canine enlargement in sabre-tooths provided a greater killing efficiency compared to the pantherine cats, the marked reduction of the postcanine dentition resulted in lesser abilities to process prey carcasses and consuming bones than in the living felids. This in turn resulted in a greater availability of scavengeable resources for other carnivores like the hyaenas (Martínez-Navarro & Palmqvist 1995, 1996; Arribas & Palmqvist 1998, 1999; Palmqvist et al. 2003, 2007). It has also been suggested that *Megantereon* had the ability to tree-store the prey leftovers, as do modern leopards (Agustí & Lordkipanidze 2019). The postcranial anatomy of *Megantereon* is overall like in the extant *Panthera onca,* which indicates the ability to use the forelimb to grapple with prey relatively large for the predator's body size. Although this is also consistent with the ability of long-distance dragging (Lewis 1997), the extremely elongated and flattened upper canines of *Megantereon* would have

been more susceptible to breakage than those of the conically toothed modern felids due to the medio-laterally directed forces that result from transporting with the mouth the unevenly distributed load of a prey carcass (Van Valkenburgh & Ruff 1987; Lewis 1997), not to mention the loads generated by climbing the prey carcass into a tree. This implies that the carcasses of the ungulate prey hunted by this sabre-tooth were unlikely to be tree-cached. Moreover, the stoutly built limb bones of *Megantereon* (Martín-Serra et al. 2017) do not indicate a scansorial locomotion in an arboreal environment. Instead, they suggest a super-ambush predatory style (Meachen-Samuels 2012), because the rotatory abilities and strength of the forelimb of *Megantereon* were probably associated with prey grappling in mixed and closed environments (Lewis 1997). This is also suggested by isotopic analyses at VM3 (Palmqvist et al. 2003, 2008a, 2008b), which indicate that browsing deer represented the bulk of its prey. Moreover, scansorial carnivores show several skeletal traits indicative of this mode of locomotion, including more curved claws, longer proximal phalanges, longer metatarsals, and longer tails than in the more terrestrial species (Van Valkenburgh 1987), traits that have not been reported in the short-tailed *Megantereon.*

Inferences on predator-prey relationships in VM3 have been derived from the abundance of carbon and nitrogen isotopes in the bone collagen of the large mammals (Palmqvist et al. 2003, 2008a, 2008b) and the use of the dual-isotope, three-source linear mixing (Phillips 2001). This approach showed that juveniles of elephant *Mammuthus meridionalis* represented a significant proportion (~10%) of the diet in the case of *H. latidens,* while bison *Bison* sp. (52%) and horse *Equus altidens* (38%) comprised the bulk of its diet. It should be noted here that remains of juvenile mammoths are abundantly represented in the faunal assemblage from Friesenhahn cave (Texas), a Pleistocene site interpreted as a den of the similarly sized North American *H. serum.*

As noted above, the elongated and laterally compressed canines of sabre-tooths were optimal for killing large prey, but were more vulnerable to fracture than the shorter, conically shaped canines of the living felids due to the unpredictable, non-directed loads generated in prey stabilization during the killing bite (Van Valkenburgh & Hertel 1993; Van Valkenburgh 2009). For this reason, the heavily muscled forelimbs of sabre-tooths were imperative for pulling down and immobilizing prey before positioning the bite over the prey throat or belly, where bite depth is essential to generate strikes reaching major blood vessels (Gonyea 1976; Akersten 1985; Anyonge 1996a; Antón et al. 2004; Christiansen 2008; Salesa et al. 2010; Andersson et al. 2011; Meachen-Samuels 2012; Martín-Serra et al. 2017). The functional need of immobilizing prey is reflected in the anatomy of the North American *Smilodon fatalis,* in which the short and robust forelimb bones are reinforced by cortical thickening to a greater degree than in the extant pantherine cats. This allowed *S. fatalis* to minimize prey struggling, helping to position the killing bite carefully to avoid contact with bone (Meachen-Samuels & Van Valkenburgh 2010; Martín-Serra et al. 2017). A pioneer study of the skulls of the iconic *S. fatalis* and the living lion based on finite element analysis (FEA) was aimed to analysing their biomechanical response to extrinsic forces, which allowed to simulate a prey struggling scenario during the killing bite (McHenry et al. 2007). This study concluded that the skull of *S. fatalis* was less equipped than the skull of the lion to resist extrinsic forces, which pointed to rapid slashing bites during prey killing. In contrast, the living big cats use their shorter and stouter canines to hold a suffocating bite in the snout of large prey such as buffalo, which explains that the role of their forelimbs while subduing prey is not as important (McHenry et al. 2007; Salesa et al. 2010; Meachen-Samuels 2012; Martín-Serra et al. 2017). Moreover, combined microanatomical analyses with FEA for simulating different prey-killing scenarios in *S. fatalis, H. serum,* and a sample of living carnivores, including the lion, showed that while the rostrum of *S. fatalis* was almost entirely composed of cortical bone, the skull of the lion has a substantial amount of trabecular bone (Figueirido et al. 2018). Given that cortical bone supports better directed loads while

trabecular bone can support unpredicted and multidirectional forces, the skull of *S. fatalis* was well-equipped to deliver a quick killing-shear bite (Figueirido et al. 2018). In contrast, the forces generated when feeding on hard objects like bones are unpredictable, which implies that the large amount of cortical bone in the skull of *S. fatalis* would be an impediment to feed on bones.

Studies of microwear textures of canines and carnassials of sabre-tooths provide additional evidence on the lower ability of these hypercarnivores to process the prey carcass compared to the living felids. Anyonge (1996b) analysed the number of pits and scratches in the upper canines of the North American *Smilodon fatalis* and several living carnivores with disparate feeding and hunting repertoires, including the spotted hyaena, the lion, the cheetah, and the wild dog. He showed that the canines of *S. fatalis* exhibited the lesser number of pits of all the canines included in the sample, which in his opinion indicated that these teeth avoided contact with bone during prey killing and feeding (Anyonge 1996b). Carnassial microwear provides a more compelling source of evidence: while the canines are directly involved in prey killing, the carnassials are used exclusively in food processing. Van Valkenburgh et al. (1990) performed scanning electron micrographs of the wear facet of the carnassial for estimating the average density, size, shape, and orientation of microwear features in a wide sample of living carnivores, including the bone-cracking spotted hyaena and the cheetah. The latter species feeds exclusively on soft organs (except the intestines) and only consumes bones of small prey (Schaller 1968; Brain 1981; Skinner & Smithers 1990). The results showed that while the carnassials of hyaenas exhibit a combination of relatively few long scratches and a high proportion of pits to scratches, the carnassials of the cheetah are characterized by a predominance of narrow scratches with very few pits. Strikingly, the microwear pattern of the carnassials of *S. fatalis* differed from the extant carnivores sampled in showing relatively narrow and long scratches combined with an extremely low frequency of pits (Van Valkenburgh et al. 1990). Concerning *Homotherium,* a recent study of dental microwear texture analysis has shown that *H. serum* consumed soft and tough foods, like the tough flesh of juvenile mammoths, and actively avoided bone (DeSantis et al. 2021). This indicates that *H. serum* probably consumed even less bone than the cheetah (DeSantis et al. 2021), the carnivore that shows the lowest degree of bone consumption among modern African carnivores (Schubert et al. 2010). The remains analysed in the study of DeSantis et al. (2021) come from the Late Pleistocene deposits of Friesenham cave, a site interpreted as a denning refuge of *H. serum* (Marean & Eckhardt 1995). Although the bone assemblage of this site preserves remains of 20 species of large mammals, it shows a low evenness: juvenile mammoths represent 31.2% of the remains, most of them of animals 2-4 years of age with an estimated mass of 500–800 kg, while 30.3% of the remains are of *H. serum,* mostly juveniles and senile individuals (Marean & Eckhardt 1995). As noted earlier, the avoidance by sabre-tooths of hard and tough tissues in the prey carcass would result in a significant quantity of resources left over to the scavengers. No study on the microwear features of the canines and carnassials of *M. whitei* is available, but it is reasonable to think that this highly specialized dirk-tooth predator would have been even, at least, as constrained as *S. fatalis* and *H. serum* to feed on soft tissues.

Other morphological features of the skull of sabre-tooths relate to the need of avoiding canine breakage when feeding on the prey carcass. For example, all sabre-tooths show a protruding incisor arcade and most of them (perhaps with the only exception of *Xenosmylus hodsonae*) exhibit marked diastemas between the third incisors and the canines. This feature would make it independent the function of the incisors in sabre-tooths, employed to tear chunks of flesh from the prey carcass (a task performed in modern felids with the assistance of their stout, conical canines), from the function of the canines, which would be exclusively used to deliver deep wounds during prey dispatch (Biknevicius et al. 1996). Therefore, prevention of canine breakage during prey killing and feeding encounters seems

to have been a strong selective force in sabre-tooths, which suggests that a non-scavenging behaviour was a clear ecological limitation imposed by their hypertrophied upper canines.

In summary, the highly derived craniodental and postcranial anatomy of sabre-tooths suggests that, compared to the pantherine felids, they: (i) were able to hunt larger ungulate prey relative to their body size, exerting a higher predation pressure on the juveniles of megafauna; and (ii) exploited their prey to a lesser extent, which would have resulted in larger amounts of flesh and all internal bone nutrients abandoned in the prey carcasses (Arribas & Palmqvist 1999; Palmqvist et al. 2003, 2007, 2011; Ripple & Van Valkenburgh 2010; Martínez-Navarro et al. 2014; Van Valkenburgh et al. 2016; Martín-Serra et al. 2017; Martínez-Navarro 2018). These resources would in turn be scavengeable by *P. brevirostris,* as documented at VM4 and VM3, and by the Oldowan hominins in the case of BL and FN-3 (Espigares et al. 2019). Interestingly, evidence of competition between hominins and hyaenas for an elephant carcass has been documented at FN-3 (Espigares et al. 2013). Given the data discussed above, the role of sabre-tooths *H. latidens* and *M. whitei* as bone accumulating and modifying agents in VM4 can be conclusively discarded.

In the case of European jaguar *P. gombaszoegensis,* a species recorded in VM3 but not in VM4, it must be noted that felids do not transport bones to denning areas placed on open air sites. Indeed, felids only accumulate bones in the karstic environment, as in the case commented above for *H. serum* and the deposits of leopard dens in caves. No taphonomic field study is available for the extant *Panthera onca,* but an experimental feeding program designed for assessing the nature and extent of alterations that captive jaguars can produce on horse bones showed that they are able of generating tooth-marks on long limb bone diaphyses and epiphyseal deletion (Rodríguez-Alba et al. 2019). However, breakage of major limb bones of medium-to-large ungulates resulting in green fractures, as those abundantly recorded at VM4 and VM3 (Arribas & Palmqvist 1998; Espigares, 2010; Palmqvist et al. 2011; Luzón et al. 2021), is clearly beyond the capacity of a jaguar. The bone modifying abilities of lions are also difficult to estimate, because these felids consume most of their prey near the kill site, where the skeletal remains are subsequently scavenged by the hyaenas, jackals and vultures. An exception could have been the man-eating lions of Tsavo, a couple of male lions reported to have caused a temporary halt to the construction of the railway between Mombasa and Lake Victoria by killing at least 135 workers (Kerbis Peterhans et al. 2001). These lions were observed dragging off some of their human prey to a home cave to feed them (Patterson 1925), a behaviour not documented among extant lions. However, when this cave was rediscovered one hundred years later, the lair was empty of bones (Kerbis Peterhans et al. 1998). More reliable information was obtained from an assemblage of wildebeest bones presumably accumulated by lions within Olduvai Gorge in the short-grassland ecological unit of the Serengeti, although these remains were probably secondarily ravaged by spotted hyaenas (Arriaza et al. 2016). This monospecific assemblage shows many complete carcasses, there are tooth-marked skeletal elements and the frequency of fractured bones (mostly small elements) is very low, a pattern of bone completeness that argues against hyaenas as the main bone modifying agent (Arriaza et al. 2016): most long bones found at spotted hyaena dens are broken and bone cylinders (i.e., isolated diaphyses) use to be abundantly represented, as in VM4 and VM3 (Arribas & Palmqvist 1998; Espigares 2010; Palmqvist et al. 2011; Luzón et al. 2021), while they are missing in the Olduvai assemblage (Arriaza et al. 2016). Finally, leopards are an effective bone accumulating agent in caves (Brain 1980, 1981), but the patterns of skeletal representation in their assemblages show differences with those collected by hyaenas: small elements like vertebrae, ribs, and phalanges represent up to 60% of postcranial remains preserved at leopard dens, while girdle and other limb bones account for the remaining 40%. In contrast, these percentages are reversed in the dens of spotted and striped hyenas, where the frequency of vertebrae, ribs and phalanges ranges between 16–

30% and 7–10%, respectively, while the percentage of girdle and limb bones (excluding phalanges) fluctuates between 70–84% and 90–93%, respectively (see review in Arribas & Palmqvist 1998). The frequencies of ribs, vertebrae, and phalanges in VM4 (16.9%) (Luzón et al. 2021) and VM3 (11.2%) (Espigares 2010) are like those typical of hyaena dens, which clearly discards a felid as the main accumulating agent of bones at both sites.

Finally, the hypercarnivorous canid *L. lycaonoides* showed a highly elaborated social behaviour, as happens in the extant African wild dog *Lycaon pictus* (Palmqvist et al. 1999; Bartolini-Lucenti et al. 2021). Wild dogs are cursorial predators that hunt daily at dawn and dusk (Creel & Creel 2019). Compared to other members of the African predatory guild, they have a high hunting success rate (44% on average, but up to 100% depending on pack size) (Creel 1997), which reflects: (i) the high predictability of the outcome of the hunt; (ii) the very high energy expenditure invested in chasing their prey; and (iii) the loss of up to half of their kills due to kleptoparasitism by other predators, particularly spotted hyaenas (Bertram 1979; Gorman et al. 1998; Hubel et al. 2016). Rather than employing hunting strategies based on speed or stealth, wild dogs rely upon chasing their prey to exhaustion. For this reason, they focus on slow prey individuals (e.g., young, pregnant, old, sick, injured, or in poor condition) more frequently than do stalking predators, and multiple kills are common in large packs (Creel & Creel 2002). Wild dogs are extremely nomadic, with packs traveling up to 50 km per day. They infrequently scavenge (Schaller 1972), as this behaviour increases encounter risk with dominant competitors like hyaenas and lions. In fact, the avoidance of these predators also explains their crepuscular activity pattern (Hayward et al. 2006). Wild dogs rear their offspring for three months in fixed sites, where the pack returns daily to provision the pups by regurgitation. The den site is usually selected in resource-scarce environments like rocky areas and mopane woodland, which are avoided by lions and spotted hyaenas, their main predators (van der Meer et al. 2013) (similarly, spotted hyaenas avoid placing their dens in areas frequented by lions; Périquet et al. 2016). In contrast to hyaenas, free-ranging canids do not regularly transport their prey to the denning site (Sanz and Daura 2018) and wild dogs are not an exception to this because their pups are fed with regurgitated meat, which makes it unnecessary to transport bones to the den. For this reason, the only skeletal remains found in the denning sites of wild dogs are the small bones digested in their scats (Fourbel et al. 2018). For all these reasons, *L. lycaonoides* can also be discarded as an accumulating agent of bones at VM4.

The evidence discussed above indicates that with the only exception of the giant hyaena *P. brevirostris,* no carnivore recorded at VM4 and VM3 can be considered as the main responsible of accumulating and modifying the huge bone assemblages preserved at these quarries. The high moment arms for masseter and temporalis muscles in the mandible of *P. brevirostris* indicate a substantial strength for bone fracturing with its well-developed premolar teeth (Palmqvist et al. 2011). Similarly, mandibular force profiles estimated using a biomechanical approach that models the mandibles of hyaenas as beams showed that the resistance of the jaw of *P. brevirostris* against dorsoventral loads during bone-cracking activities was substantially greater than in the living hyaenas; this is particularly evident in the jaw symphysis, which is extremely buttressed dorsoventrally and thus better suited for fracturing bones than in the modern hyaenids (Palmqvist et al. 2011). Moreover, a recent ecomorphological study has shown that *P. brevirostris* was specialized in non-dragging carcass transport during long distances (Pérez-Claros & Coca-Ortega 2020): in this species, as happens in *Hyaena hyaena* and *Parahyaena brunnea,* the two extant scavenging durophagous hyaenas, the lower canines are comparatively more developed than in *Crocuta Crocuta,* which behaves both as a hunter and a scavenger (Palmqvist et al. 1996). This represents an adaptation to carry heavy loads to the denning site (Pérez-Claros & Coca-Ortega 2020), as reflected in the mean distance of transport of the remains: 6.50 ± 2.53 km in *P. brunnea* (Kuhn et al. 2008) and 0.56 ± 0.08 km in *C. crocuta* (Skinner et al.

1986). However, it is important to note that spotted hyenas transport the remains over greater distances than leopards (0.18 ± 0.03 km) (Brain 1981). Compared to the living hyaenas, *P. brevirostris* also shows a shortening of the distal limb segments, which represents another adaptation for long-distance transport of ungulate carcasses: the brachial index (radius length/humerus length) and the crural index (tibia length/femur length) take in *P. brevirostris* lower values, 0.91 and 0.74, respectively, than in *C. Crocuta* (1.08 and 0.82), *H. hyaena* (1.07 and 0.88) and *P. brunnea* (1.00 and 0.82). Although such shortening may be explained in part as resulting from the enormous size of this extinct hyena, given the inverse relationship in modern carnivores of the brachial and crural indexes with body mass (Anyonge 1996a), the tibia of *P. brevirostris* seems to have been particularly short in relation to the femur. This reduction has been envisioned in hyenas as an adaptation to carry large pieces of carcasses without dragging (Spoor 1985). Modern hyenas show a comparatively long radius, which raises the forequarters and therefore the head of the animal. Although the radius is also shortened in *P. brevirostris* compared to other hyenas, the ratio between the fore- and hindlimb lengths (1.06) is close to the value of brown hyenas (1.06). This is achieved by the extreme shortening of the tibia in the giant hyena, which indicates a similar emphasis on the forequarters to that seen in brown hyenas. This feature suggests a less cursorial lifestyle for *P. brevirostris* than in the spotted hyena, although the shortening of the limbs provided greater power and more stability to dismember and carry large pieces of ungulate carcasses (Turner & Antón 1996; Palmqvist & Arribas 2001; Palmqvist et al. 2011). Moreover, the assemblages of VM4 and VM3 preserve remains of animals that lived in different habitat types (e.g., grazing horse and bison from open plains, browsing deer from forest areas, hippo from aquatic environments, and muskox from mountain areas), which suggests a large survey area by the giant hyaenas and long transport distances. In addition, the NISP values calculated for the remains accumulated by *P. brevirostris* in the earthen dens of VM4 and VM3, several thousands in both cases, are two orders of magnitude higher than those observed for brown and spotted hyaenas in this type of den (87.8 ± 61.8 and 99.6 ± 90.4, respectively) and are even higher than in the cave dens of brown hyaenas (763.6 ± 197.5), in spite of the fact that the karstic environment facilitates a greater accumulation of remains (Lansing et al. 2009).

Given the data discussed above, it is very difficult to argue on the possibility that other carnivore than the giant hyaena *P. brevirostris* could be responsible of generating the huge bone accumulations assemblages preserved at VM4 and VM3. Moreover, although the high frequency of juveniles among the ungulates of VM4 (46.3%, 19/41) and VM3 (43.6%, 125/287) (Table 1) could suggest, at first sight, that these bone assemblages were accumulated by a primary predator, which focused on young individuals (more vulnerable to predation), most prey individuals of those assemblages collected by primary predators tend to fall within a rather restricted body size range around the predator’s size and there uses to be also a relatively low diversity of prey species from a limited environmental range, according with the predator's preferences (Arribas & Palmqvist 1998). This is clearly not the case at VM4 and VM3, which record a high diversity of ungulates (Table 1) with a variety of feeding/habitat preferences and a body mass range that covers nearly three orders of magnitude (~10–6000 kg) (Palmqvist et al. 1996; Arribas & Palmqvist 1998). In addition, the carnivore:ungulate ratio uses to be high (~0.4) in the assemblages collected by primary predators like leopards, which often prey on smaller carnivores (Brain 1981), while it tends to be lower (~0.2) in the assemblages collected by hyaenas, although there is substantial variation between spotted hyaenas, striped hyaenas, and brown hyaenas (Stewart et al. 2021). The values of this index are comparatively low in VM4 (0.27, 11/41 according to Luzón et al. and 15/55 in our own data) and VM3 (0.18, 53/287) (Table 1), which argues for the scavenging *P. brevirostris* as the bone accumulating agent of both assemblages. Similarly, two ratios have been proposed for estimating the intensity of ravaging in an assemblage (Domínguez-Rodrigo & Organista 2007): (i) axial bones to appendicular bones,

which would range between 4.25 for a carcass transported complete or died in a setting devoid of competition among carnivores, to 0 for a completely ravaged skeleton; and (ii) (proximal humerus plus distal radius)/(distal humerus plus proximal radius), which measures the relative abundance of the least dense bone portions (which are preferentially consumed by the scavengers) and takes a value comprised between 1 in an undisturbed carcass and 0 in the situation of highest ravaging intensity. The values of the first ratio differ between VM4 (0.37, 92/250; Luzón et al. 2021) and VM3 (0.13, 502/3,721; Espigares 2010), but the difference is lower according to our own data for VM4 (0.28, 243/882). This indicates that although both assemblages were biased by the selective transport by hyaenas of skeletal parts and body portions to their maternity dens, with a clear preference for the limbs in the case of medium-to-large sized ungulates (Palmqvist & Arribas 2001), VM3 shows a greater intensity of ravaging, as expected from the longer time of exposure of the remains in this quarry (which is evidenced by the more advanced degree of bone weathering). In the case of the second ratio, Luzón et al. (2021) do not provide separate counts for proximal and distal epiphyses of major limb bones in VM4, and our own data are too preliminary to calculate reliably this ratio. However, the value in VM3 (0.32, 89/274) (Espigares 2010) also agrees with the expectations for a ravaged assemblage. All this indicates that the bone accumulations of VM4 and VM3 were most probably generated by a secondary carnivore like *P. brevirostris,* which was specialized in scavenging the prey hunted selectively upon by other predators (Palmqvist et al. 1996; Arribas & Palmqvist 1998; Palmqvist & Arribas 2001; Espigares 2010; Palmqvist et al. 2011).

Palaeoecological reconstruction of Venta Micena

Luzón et al. (2021) affirm that the faunal composition of the bone assemblages of VM4 and VM3 indicates a similar palaeoclimatical setting for both sites, dominated by warm and drier conditions than those suggested by the fauna present at Barranco León (BL) and Fuente Nueva-3 (FN-3), two sites of the Baza Basin that are slightly younger (1.4–1.3 Ma) than VM (1.6–1.5 Ma) (Palmqvist et al. 2016). This conclusion is based on the presence/absence of several water dependent species of amphibians and reptiles in the assemblages of small vertebrates from these sites (Agustí et al. 2010; Blain et al. 2011, 2016). However, herpetofaunal remains are poorly represented in VM3 and VM4 (only three species recorded) compared to BL (22 species) and FN-3 (16 species) (Blain et al. 2016), which casts serious doubts on this comparison. In contrast, the composition of the assemblages of large mammals of VM, BL, and FN-3 provides evidence that favours the opposite interpretation.

First, isotopic analyses of the ungulates from VM3 (Palmqvist et al. 2003, 2008a, 2008b; García-Aguilar et al. 2015) indicate the presence of six grazing species (horse *E. altidens,* buffaloes *Bison* sp. and *Hemibos* cf. *gracilis,* muskox *Praeovibos* sp., goat *Hemitragus albus,* and elephant *M. meridionalis*), one species that exclusively fed on aquatic vegetation (hippo *H. antiquus*), two mixed feeders that fed on grass and leaves (ovibovine *Soergelia minor* and fallow deer *Metacervocerus rhenanus*) and two browsers (deer *Praemegaceros* cf. *verticornis* and rhino *Stephanorhinus* cf. *hundsheimensis*). This fauna agrees with the expectations for an open plain with patches of bush and forest (Mendoza et al. 2005). Although the faunal assemblages of BL and FN-3 are similar to the one of VM, they record a second horse species, the large sized *Equus sussenbornensis,* and the mesodont *S. minor* is replaced by *Ammotragus europaeus,* a hypergrazing caprine with very hypsodont teeth (Moulé et al. 2004; Martínez-Navarro et al. 2010; Rodríguez-Gómez et al. 2016, 2017). The presence of *E. sussenbornensis* and *A. europaeus,* two species adapted to open plains, indicates that the environment of these sites was clearer of trees than in VM. A mesowear analysis (Saarinen et al. 2021) based on a small sample of teeth from BL, FN-3, and VM has suggested that *E. sussenbornensis* had more browsing habits than *E.*

altidens and that the diet of the latter species was more grass-dominated in VM than in the two other sites. Despite this, the record of two grazing equids in BL/FN-3 suggests more arid conditions and an herbage of lower quality than in VM (contra Saarinen et al. 2021). The reason is that the forestomach fermentation of ruminants is in advantage under conditions of limiting quantities of food: compared to hindgut fermenting monogastric herbivores like horse, rhino, and elephant, ruminants are very efficient at extracting maximum amounts out of the cellulose and cell contents of food of moderate fibre content; for this reason, if feeding on food of relatively good quality they can subsist on a lesser amount of food per day (~70%) than monogastric herbivores of similar body size, which rely on rapid passage times and the processing of large quantities of food (Janis 1976; Janis et al. 1984; Duncan et al. 1990). If food availability is not a limiting factor, however, equids can feed on low quality grasses too fibrous for a ruminant to subsist on, such as herbage with high fibre contents, because they process a large volume of food in a short time (see review in Palmqvist et al. 2008a). For this reason, the presence of two horses in BL/FN-3 indicates a more arid and open environment compared to the previous conditions in VM4 and VM3.

Second, several studies have focused on modelling the trophic relationships among the large mammal species identified in the Orce sites and estimating prey carrying capacity in these ancient ecosystems. These analyses were based on a model that estimates the amount of meat available to the secondary consumers (i.e., carnivores and scavengers) and its distribution among these species (Rodríguez-Gómez et al. 2013, 2014a, 2014b, 2016, 2017). The model computes: (i) the age structure that makes the population of each primary consumer stable and stationary; (ii) the distribution of individuals among adults and non-adults; (iii) the biomass of ungulates that can be extracted in the long term by the secondary consumers; (iv) the dietary requirements of the secondary consumers when they reach their maximum population densities; and (v) the distribution of prey biomass among the secondary consumers, which allows to estimate the sustainable density of each carnivore and scavenger (Rodríguez-Gómez et al. 2013, 2014a, 2014b; Martín-González et al. 2019). This approach provided estimates of yearly available biomass (kcal/km^2) in VM that were ~25–30% higher than those of BL/FN-3 (Rodríguez-Gómez et al. 2016, 2017; Martín-González et al. 2019), which indicates a more productive ecosystem for the secondary consumers in VM and argues for a higher rainfall. In which concerns prey carrying capacity, data compiled in Figure S6 for a set of African Natural Parks and Game Reserves (Hatton et al. 2015; Fick and Hijmans 2017) shows a direct and statistically significant relationship between prey biomass (kg/km^2) and annual rainfall (mm). It must be noted, however, that there is considerable scatter around the least-squares regression line, which mainly depends on soil type (Bell 1981) and on the presence of thermal springs (Fig. S6). Figure S6 also includes the mean estimates of carrying capacity of the Orce sites, 3,827.8 kg/km^2 for VM and 3,307.6 kg/km^2 for BL/FN-3, obtained using an approach based on the Weibull model (Martín-González et al. 2019; Rodríguez-Gómez et al. 2022). Therefore, both meat availability and carrying capacity indicate a more productive environment in VM than in BL/FN-3. Moreover, given the direct relationship between prey biomass and annual rainfall (Watson 1972; Coe et al. 1976; Bell 1981), our rainfall estimates for the Orce sites (Fig. S6) suggest that climatic conditions were more humid in VM3 (832 mm) than in BL/FN-3 (639 mm). In a recent paper, however, mean values of annual precipitation, minimum annual temperature, and net primary production were estimated from dental ecometrics of the large herbivorous mammals from several sites of the Guadix-Baza Depression (Saarinen et al. 2021). In contrast to our estimates, the results of this study argued for a lower annual precipitation, a higher temperature, and a lower net primary production in VM than in FN-3 and BL (Saarinen et al. 2021). However, these results are not consistent with the number of large mammals recorded at these sites: 21 species in VM (13 primary consumers and eight secondary consumers; Rodríguez-Gómez et al. 2017) and 18 species in FN-3 and BL (10 primary consumers and eight secondary consumers, including humans; Rodríguez-Gómez

et al. 2016), which also argues for a higher annual rainfall in VM. On the other hand, isotopic data do not support the low estimate of mean annual precipitation obtained in the study of Saarinen et al. (2021) for VM, only 449 mm. More specifically, $\delta^{15}N$ values in the bone collagen of herbivorous mammals correlate negatively with annual rainfall. The range of $\delta^{15}N$ values in VM (1.2–7.7‰) (Palmqvist et al. 2003, 2008a, 2008b) is like the one recorded at Kasungu National Park, Malawi (1.6–7.2‰) (Sealy et al. 1987), which has an average rainfall of 780 mm per year (a value that doubles present-day precipitations in the Baza Basin) (García-Aguilar et al. 2014). This estimate is close to the one deduced here for VM, 832 mm (Fig. S6), and higher that the estimates obtained for FN-3/BL, 639 mm according to data in Figure S6 and 741–753 mm based on fossil remains of water-associated amphibians and reptiles (Blain et al. 2011).

Taxonomy of the large mammals preserved in Venta Micena

Luzón et al. (2021) indicate that similar species of large mammals are present in VM4 and VM3. However, despite the wealth of publications on the taxonomy of these species, they revise and update the faunal list of the site with wrong taxonomic assignments. For example, they include the rhino *Stephanorhinus etruscus,* which in their opinion was misidentified in previous publications as *S. hundsheimensis.* Similarly, they identify the small-to-medium sized caprine *H. albus* as *Capra alba* and change the specific name of sabre-tooth *M. whitei* to *Megantereon cultridens.* Unfortunately, they do not perform a systematic study of these taxa and only provide some brief explanations. Moreover, they maintain most of the taxonomical adscriptions that were performed during last fourth decades in the site since the first publication of the faunal list of Venta Micena by Moyà-Solà et al. (1981), which has been revised later in several studies (e.g., Moyà-Solà et al. 1987; Martínez-Navarro 1991; Cregut-Bonnoure 1999; Martínez-Navarro et al. 2011; Palmqvist et al. 2011; Ros-Montoya et al. 2012; among others). Despite this, Luzón et al. (2021) disqualify in some cases the previous determinations without providing any real argument or performing a new comparative study of the species concerned. This is especially clear when they write on the rhinoceros, the hunting dog *Canis (Xenocyon),* and the sabre-tooth *Megantereon,* but also applies to other taxa. The most important problem here is that they cite a lot of references that they do not seem to have read, which leads them to confusing, mistaking, and inventing the data, as explained below.

For example, Luzón et al. (2021) change the name of the species *Stephanorhinus* aff. *hundsheimensis* to *Stephanorhinus etruscus* Toula, 1902. This rhino was firstly described at Venta Micena as *Dicerorhinus etruscus brachycephalus* (Santafé-Llopis & Casanovas-Cladellas 1987), following the taxonomy of Guerin (1980). *Dicerorhinus* is an endemic form, nowadays represented in South-eastern Asia at the islands of Sumatra and Borneo by the species *D. sumatrensis,* although its fossil record includes all of Indochina and southern Himalayan region. However, the lineage of *Dicerorhinus etruscus* is not the one found around all the Plio-Pleistocene of Eurasia, as noted by Guerin (1980). For this reason, Fortelius et al. (1993) ascribed the species to the genus *Stephanorhinus* Kretzoi, 1942, and considered that it was a mistake to create the subspecies *brachycephalus,* because it was described on the base of a specimen from the German site of Daxlanden which, in fact, must be ascribed to the Middle and Late Pleistocene species *Stephanorhinus kirchbergensis.* Fortelius et al. (1993) considered as valid the species *S. etruscus,* which is recorded during the Early to Late Villafranchian (Pandolfi et al. 2017), and *S. hundsheimensis* for the Epivillafranchian and the base of the Middle Pleistocene, giving to these species an important biochronological value. After the publication of Santafé-Llopis & Casanovas-Cladellas (1987), nobody has published an in-depth study of the fossil rhino of VM, but it was revised by F. Lacombat during his visit to Orce in 2005, who suggested to

call it as *S.* aff. *hundsheimensis* (i.ex. Ros-Montoya et al. 2012; Rodríguez-Gómez et al. 2017), which means that it is a new form closely related to *S. hundsheimensis,* but different. Unfortunately, this study has not been yet published. Now, without an in-depth study of the rhino materials from the site, Luzón et al. (2021) conclude that the VM rhino must be ascribed to *S. etruscus.* This conclusion is not based on any anatomical or metrical analyses of the few fossils that they study. Instead, it is based on the chronology of the remains, which provides an example of circular reasoning of the type "if it is Tuesday, this must be Belgium" (Palmqvist et al. 2016). More specifically, they textually affirm in their supplementary text that *"Since the intended concept of Guerin's nomenclature Dicerorhin*us etruscus brachycephalus *essentially maps onto* Stephanorhinus hundsheimensis, *this name, usually denoted with a cautionary "cf.", has more or less automatically been applied to the Venta Micena rhinoceros in recent decades (Martínez Navarro et al. 2014; Rodríguez-Gómez et al. 2017; Palmqvist et al. 2018). This is surprising because elsewhere the species is typical of the Middle rather than Early Pleistocene."* It is obvious that Luzón et al. (2021) do not consider (or do not understand) which is the difference between "cf." (i.e., which may be this species) and "aff." (i.e., which is a species similar to, but different), and they also misrepresent the contents of the cited references, because Martínez-Navarro et al. (2014) do not write on rhinocerothids (also, the reference Palmqvist et al. is 2016, not 2018). Finally, there is a recent paper (Pandolfi et al. 2021) on the fossil rhinos from the Georgian site of Dmanisi, dated to 1.8 Ma. In this study, based on a couple of crania, two morphotypes of rhinoceros have been described, one with browsing affinities and the other with features that indicate a grazing diet, which opens the possibility that two species of rhinos inhabited in the same environment at Dmanisi (and also in the Late Villafranchian of European).

With very few arguments, Luzón et al. (2021) also discredit the published studies on the canids from Venta Micena. First, they affirm in the first paragraph that *"Two species are currently recognized at Venta Micena (e.g., Medin et al. 2017), instead of the three identified originally by Pons-Moya (1987)."* We do not know what they consider as canids, because the species of canids listed in the faunal list published by Medin et al. (2017) are *Lycaon lycaonoides, Canis mosbachensis,* and *Vulpes praeglacialis.* Maybe, they consider that foxes are not canids. Obviously, the study of Medin et al. (2017), which was entitled *"Late Villafranchian* Ursus etruscus *and other large carnivorans from the Orce sites (Guadix-Baza basin, Andalusia, southern Spain): Taxonomy, biochronology, paleobiology, and ecogeographical context",* did not include the genus *Vulpes,* as the latter comprises small-sized carnivores. Therefore, it seems clear that they did not read this paper (nor even paid attention to its title). By the way, Pons-Moyà (1987) cited the presence of four canid genera (not three) at Venta Micena (*Xenocyon, Cuon, Canis,* and *Vulpes*), but the presence of *Cuon* was based on a badly restored lower carnassial. When this tooth was correctly restored, it was clear that it belonged to the genus *Canis* (Martínez-Navarro 1991). Similarly, Luzón et al. (2021) change with no taxonomic criteria the name of the hypercarnivorous hunting dog of Venta Micena, *Lycaon lycaonoides,* which was published by Martínez-Navarro & Rook (2003), to *Xenocyon (Lycaon) lycaonoides.* In doing so, they do not realise that *Xenocyon* was synonymized in the same genus with *Lycaon* (Martínez-Navarro & Rook 2003). Instead, they follow the arguments of Hartstone-Rose et al. (2010), who tried to separate *Lycaon* from *Xenocyon* based on anatomical criteria. For this reason, they write that *"The phylogenetic analysis by Zrzavý et al. (2018) ruled out any close affinity between* Lycaon *and* Xenocyon. *The two genera differ, for instance, in the morphology of the paranasal sinuses as well as in dental characters."* Then, the obvious question here is: if they think that *Xenocyon* and *Lycaon* are quite different genera, why do they mix both names when they describe the hunting dog of Venta Micena as *Xenocyon* (*Lycaon*) *lycaonoides*? Moreover, there is a recent paper (not cited by Luzón et al. 2021) that uses anatomical and morphometric data of a large database that incorporates most of the records of fossil hunting dogs from the late Early Pleistocene of Europe, Asia, Africa, and North America. In this

paper (Bartolini-Lucenti et al. 2021), the hunting dog of Dmanisi and VM is classified as *Canis* (*Xenocyon*) *falconeri.*

Probably, the most significant change of a specific name concerns the sabre-tooth *Megantereon.* As noted by Luzón et al., Turner grouped all the species published of this genus in *Megantereon cultridens,* because he interpreted that the anatomical and metrical variability within the genus was the reflection of a high level of sexual dimorphism combined with geographic differences (Turner 1987). Later, a review of the hypotheses on the origin, evolution, and dispersal of *Megantereon* based on the comparison with multivariate methods of all published craniodental specimens from Africa, Eurasia, and North America led to the conclusion that only two species were valid, *M. cultridens* and *M. whitei* (Martínez-Navarro & Palmqvist 1995, 1996). Under the new interpretation, *M. cultridens* would be the primitive form in the New World and the one found in Europe until middle Early Pleistocene times (i.e., Middle Villafranchian). The second species, *M. whitei,* would group all Plio-Pleistocene African specimens (Palmqvist 2002) as well as those from several localities in the North and East of the circum-Mediterranean region (e.g., Apollonia-1 in Greece; Argentario and Pirro Nord in Italy; Untermassfeld in Germany; Urkút in Hungary; Venta Micena in Spain; Ubeidiya in Israel; Dmanisi in Georgia), where *M. cultridens* was replaced by *M. whitei* in the late Early Pleistocene (i.e., Late Villafranchian).

The African species, *M. whitei,* shows an extreme reduction in the size of the third lower premolar, which results in the appearance of a diastema between this tooth and the fourth lower premolar, as well as a significant reduction in the dimensions of latter tooth, while only minor differences are recorded between both species in the size of the lower carnassial (Martínez-Navarro & Palmqvist 1995, 1996), the single tooth analysed in the previous study (Turner 1987). The derived *M. whitei* arrived at the gates of Europe at ~1.8 Ma, as evidenced in Dmanisi, which was coincident with the first hominin dispersal out of Africa (Arribas & Palmqvist 1999; Rook et al. 2004; Martínez-Navarro 2004, 2010; Bartolini-Lucenti et al. 2021). Turner (1987) was aware that the poor fossil record of these hypercarnivores, combined with the presumed anatomical and metric differences between both sexes, made it difficult to compare the specimens of *Megantereon* from different localities. For this reason, he measured the size of the lower carnassial in a large sample of *P. pardus* and concluded that the metric differences recorded in *Megantereon* did not exceed those derived from sexual dimorphism and geographic variation in extant felids of similar size. For this reason, some researchers considered the possibility that most of the relatively few European fossils attributed to African *M. whitei* (Martínez-Navarro & Palmqvist 1995, 1996), which lower carnassials are of smaller size on average than those of *M. cultridens,* could be female individuals of the Eurasian and North American *M. cultridens.* This issue was further clarified in a new study (Palmqvist et al. 2007), co-authored also by Alan Turner, which was intended to: (i) re-evaluate the systematics of *Megantereon* with fossil specimens not included in previous analyses; and (ii) test the hypotheses of sexual dimorphism and geographic differences with a large sample of sexed leopards and lions from several African and Asian populations. The results obtained showed that the differences in tooth measurements between *M. cultridens* and *M. whitei,* particularly in the size of the fourth lower premolar, exceeded by far those expected from sexual dimorphism and geographic variation in leopards and lions, which ruled out that these differences could result from biased sampling in *M. cultridens* and *M. whitei* (Palmqvist et al. 2007). Moreover, this study showed also two previously unrecognized features of the dentition of *M. whitei:* (i) while both species of *Megantereon* show a protoconid (i.e., the cusp more posteriorly positioned in the lower carnassial) of similar length, the paraconid (i.e., the cusp more anteriorly positioned) is shorter in *M. whitei* than in *M. cultridens,* which parallels the reduction of the third and fourth lower premolars in *M. whitei;* and (ii) although the cheek

teeth tend to be smaller in *M. whitei* than in *M. cultridens,* the former species has on average larger upper canines (Palmqvist et al. 2007).

Although there are some researchers that do not accept this hypothesis, for example Lavrov et al. (2021), who call the species found at Taurida Cave (Crimea) as *Megantereon adroveri,* a younger synonym of *M. whitei* described in VM by Pons-Moyà (1987), the African origin of this sabre-tooth arriving in Europe at the base of the Late Villafranchian is now assumed by most of the scientific community.

As discussed before, the hypertrophy of the upper canines in sabre-tooth predators resulted in the need of increasing the mandibular gape for clearing the tips of the canines when delivering the killing bite (Fig. S5). This involved the reorganization of the jaw-adducting muscles for avoiding the over-stretching of temporalis fibres during wide gaping while retaining bite force at the carnassial. A number of changes in the craniodental anatomy of sabre-tooths compared to the pantherine felids allowed to achieve this (Emerson & Radinsky 1980; Akersten 1985; Palmqvist et al. 2007; Slater & Van Valkenburgh 2008; Figueirido et al. 2011), including: (i) a lowered glenoid fossa, which resulted in a ventral displacement of the jaw joint; (ii) a shortening of the coronoid process of the mandible, an upward rotation of the palate relative to the braincase, a shorter and narrower temporal fossa, and a more vertical occiput, which all resulted in a narrowing of the temporalis fibres and their more perpendicular orientation to the tooth row; and (iii) a laterally shifted angular process in the mandible and a less laterally projected postglenoid process in the cranium, which both helped the mandible to avoid contact with the postglenoid process when abducting the lower jaw (see Fig. S5). A major consequence of this skull reorganization in sabre-tooths for maximizing jaw opening was that it posed a biomechanical constraint on the point of maximum bite force exerted at the carnassial, which was positioned more backwardly than in the extant felids. Moreover, the masseter muscle, which exerts its maximum force at smaller gapes, is also reduced. For this reason, the enlargement of the sabres in *M. whitei* resulted in a reduction of the post-canine dentition not related to the slicing function of the carnassial (Palmqvist et al. 2007). This is particularly evident in the case of the third lower premolar, which is reduced to a vestigial peg or even lost, as in the specimen from South Turkwel, Kenya (Palmqvist 2002). To a lesser degree, this is also true for the fourth lower premolar and even for the paraconid of the carnassial, which are both shortened in relation to their dimensions in the less advanced *M. cultridens* (Palmqvist et al. 2007). Therefore, although the greater development of the upper canines in *M. white* resulted in a greater killing efficiency compared to *M. cultridens,* it further limited the ability of this predator to process the prey carcass, which in turn resulted in a greater availability of scavengeable resources for scavengers like the hominins and hyaenas. This provides the ecological connection between the dispersal of *M. whitei* out of Africa and the first human arrival in Europe, recorded in the Orce sites of BL and FN-3 (Espigares et al. 2013, 2019), a continent where the survival of hominin populations during the cold season with lowered plant resources probably depended on the regular scavenging of ungulate carcasses (Turner 1992; Martínez-Navarro & Palmqvist 1995, 1996; Arribas & Palmqvist 1999; Palmqvist et al. 2007).

References

Agustí, J., de Marfá, R. & Santos Cubedo, A. Roedores y lagomorfos (Mammalia) del Pleistoceno inferior de Barranco León y Fuente Nueva 3 (Orce, Granada) in: *Ocupaciones Humanas en el Pleistoceno inferior y medio de la cuenca de Guadix-Baza, Memoria Científica* (eds. Toro, I., Martínez-Navarro, B. & Agustí, J.) 121–140 (Consejería de Cultura Junta de Andalucía, Arqueología Monográfico, 2010).

Agustí, J. & Lordkipanidze, D. An alternative scenario for the first human dispersal out of Africa. *L'Anthropologie* **123**, 682–687 (2019).

Agustí, J., Oms, O., Garces, M., Pares, J.M. Calibration of the late Pliocene early Pleistocene transition in the continental beds of the Guadix Baza Basin (South Eastern Spain). *Quat. Int.* **40**, 93–100 (1997).

Akersten, W.A. Canine function in *Smilodon* (Mammalia: Felidae: Machairodontidae). *Los Angeles County Mus. Contr. Sci.* **356**, 1–22 (1985).

Albesa, J. & Robles F. Contribución al conocimiento de los moluscos del Pleistoceno Inferior del yacimiento de Barranco León (Cuenca de Guadix-Baza). *Span. J. Paleontol.* **35**, 125–146 (2020).

Anadón, P., Julià, R., De Deckker, P., Rosso, J.-C. & Söuié-Marsche, I. Contribución a la Paleolimnología del Pleistoceno inferior de la cuenca de Baza (sector Orce-Venta Micena). *Paleontologia i Evolució, Mem. Esp.* **1**, 35–72 (1987).

Anadón-Monzón, P. Sedimentación lacustre, in: *Ciclos de seminarios de sedimentología 1* (Coord. Gabaldón, V.) 93–131 (Madrid Instituto Geológico y Minero de España, 1984).

Andersson, K., Norman, D. & Werdelin, L. Sabretoothed carnivores and the killing of large prey. PLoS ONE **6(10)**, e24971. doi:10.1371/journal.pone.0024971

Antón, M. *Sabertooth (Life of the Past)*. (Bloomington Indiana University Press, 2013).

Antón, M., Galobart, A. & Turner, A. Co-existence of scimitar-toothed cats, lions and hominins in the European Pleistocene. Implications of the post-cranial anatomy of *Homotherium latidens* (Owen) for comparative palaeoecology. *Quat. Sci. Rev.* **24**, 1287–1301 (2005).

Antón, M., Salesa, M.J., Pastor, J.F., Sánchez, I.M., Fraile, S. & Morales, J. Implications of the mastoid anatomy of larger extant felids for the evolution and predatory behaviour of sabretoothed cats (Mammalia, Carnivora, Felidae). *Zool. J. Linn. Soc.* **140**, 207–221 (2004).

Anyonge, W. Body mass in large extant and extinct carnivores. *J. Zool.* **231**, 339–350 (1993).

Anyonge, W. Locomotor behaviour in Plio-Pleistocene sabre-tooth cats: a biomechanical analysis. *J. Zool.* **238**, 395–413 (1996a).

Anyonge, W. Microwear on canines and killing behavior in large carnivores: saber function in *Smilodon fatalis. J. Mammal.* **77**, 1059–1067 (1996b).

Arriaza, M. C., Domínguez-Rodrigo, M., Yravedra, J. & Baquedano, E. Lions as bone accumulators? Paleontological and ecological implications of a modern bone assemblage from Olduvai Gorge. PLoS ONE **11(5)**: e0153797. doi:10.1371/journal.pone.0153797 (2016).

Arribas, A. & Palmqvist, P. Taphonomy and paleoecology of an assemblage of large mammals: hyaenid activity in the lower Pleistocene site at Venta Micena (Orce, Guadix-Baza Basin, Granada, Spain). Geobios **31** (suppl.), 3–47 (1998).

Arribas, A. & Palmqvist, P. On the ecological connection between sabre-tooths and hominids: faunal dispersal events in the lower Pleistocene and a review of the evidence for the first human arrival in Europe. *J. Archaeol. Sci.* **26**, 571–585 (1999).

Barnett, R., Westbury, M.V., Sandoval-Velasco, M., Vieira, F.G., Jeon, S., Zazula, G., Martin, M.D., Ho, S.Y.W., Mather, N., Gopalakrishnan, S., Ramos-Madrigal, J., de Manuel, M., Zepeda-Mendoza, M.L., Antunes, A., Carmona Baez, A., De Cahsan, B.,

Larson, G., O'Brien, S.J. & Eizirik, E. Genomic adaptations and evolutionary history of the extinct scimitar-toothed cat, Homotherium latidens. *Curr. Biol.* **30**, 1–8 (2020).

Bartolini-Lucenti, S., Madurell-Malapeira, J., Martínez-Navarro, B., Palmqvist, P., Lordkinanidze, D. & Rook, L. The early hunting dog from Dmanisi with comments on the social behaviour in Canidae and hominins. *Sci. Rep.* **11**, 13501. https://doi.org/10.1038/s41598-021-92818-4 (2021).

Bell, R. H. V. The effect of soil nutrient availability on community structure in African ecosystems in *Ecology of Tropical Savannas. Ecological Studies (Analysis and Synthesis)* (eds. Huntley B. J. & Walker B. H.), Springer, Berlin, Heidelberg **42** (1981).

Bertram, B. C. B. Serengeti predators and their social systems in *Serengeti: dynamics of an ecosystem* (eds. Sinclair, A. R. E. & Norton-Griffiths, M.) (Chicago University of Chicago Press, 221–285, 1979).

Biknevicius, A.R., Van Valkenburgh, B. & Walker, J. Incisor size and shape: implications for feeding behavior in sabertoothed "cats." *J. Vert. Paleontol.* **16**, 510–521 (1996).

Blain, H.A., Bailón, S., Agustí, J., Martínez-Navarro, B. & Toro I. Paleoenvironmental and paleoclimatic proxies to the Early Pleistocene hominids of Barranco León D and Fuente Nueva 3 (Granada, Spain) by means of their amphibian and reptile assemblages. *Quat. Int.* 243, 44–53 (2011).

Blain, H. A., Lozano-Fernández, I., Agustí, J., Bailón, S., Menéndez, L., Espigares, M. P., Ros-Montoya, S., Jiménez-Arenas, J., Toro-Moyano, I., Martínez-Navarro, B. & Sala, R. Refining upon the climatic background of the Early Pleistocene hominid settlement in Western Europe: Barranco León and Fuente Nueva-3 (Guadix-Baza Basin, SE Spain). Quat. Sci. Rev. **144**, 132–144 (2016).

Bond, G., Showers, W., Cheseby, M., Lotti, R., Almasi, P., deMenocal, P., Priore, P., Cullen, H., Hajdas, I. & Bonan, G. A. Pervasive millennial-scale cycle in North Atlantic Holocene and glacial climates. *Science* **278**, 1257–1266 (1997).

Braga, J.C., Martín, J.M. & Quesada, C. Patterns and average rates of late Neogene–Recent uplift of the Betic Cordillera, SE Spain. *Geomorphology* **50**, 3–26 (2003).

Brain, C. K. Some criteria for the recognition of bone-collecting agencies in African caves in *Fossils in the Making* (eds. Behrensmeyer, A. K. & Hill, A.P.) (Chicago The University of Chicago Press, 108–130, 1980).

Brain, C. K. *The Hunters or the Hunted: An introduction to African Cave Taphonomy.* (Chicago The University of Chicago Press, 1981).

Britton, R.H. Life cycle and production of *Hydrobia acuta* Drap. (Gastropoda: Prosobranchia) in a hypersaline coastal lagoon. *Hydrobiologia* **122**, 219–230 (1985).

Carboneras, C. & Kirwan, G.M. Common Shelduck (*Tadorna tadorna*) in *Handbook of the Birds of the World Alive* (eds. del Hoyo, J, Elliott, A, Sargatal, J, Christie, DA & de Juana, E.) (Barcelona Lynx Edicions, 2018).

Christiansen, P. Evolution of skull and mandible shape in cats (Carnivora: Felidae). *PLoS One* **3(7)**, e2807 (2008). doi:10.1371/journal.pone.0002807

Christiansen, P. & Adolfssen, J. S. Osteology and ecology of *Megantereon cultridens* SE311 (Mammalia; Felidae; Machairodontinae), a sabrecat from the Late Pliocene – Early Pleistocene of Senéze, France. *Zool. J. Linn. Soc.* **151**, 833–884 (2007).

Coe, M. J., Cumming, D. H. & Phillipson, J. Biomass and production of large African herbivores in relation to rainfall and primary production. *Oecologia* **22**, 341–354 (1976).

Creel, S. Cooperative hunting and group size: assumptions and currencies. Anim. Behav. **54**, 1319–1324 (1997).

Creel, S. & Creel, N. M. *The African Wild Dog: Behavior, Ecology, and Conservation.* (Princeton Princeton University Press, 2002).

Creel, S. & Creel, N. M. *The African Wild Dog. Monographs in Behavior and Ecology* **65** (2019). https://doi.org/10.1515/9780691207001

Cregut-Bonnoire, E., Les petits Bovidae de Venta Micena (Andalousie) et de Cueva Victoria (Murcia) in *The Hominids and their environment during the Lower and Middle Pleistocene of Eurasia* (eds. Gibert, J., Sánchez, F., Gibert, L. & Ribot, F.) *Proc. Int. Conf. Human Paleont.*, Orce 1995, 191–228 (1999).

DeSantis, L.R.G., Feranec, R.S., Antón, M. & Lundelius, E.L. Dietary ecology of the scimitar-toothed cat *Homotherium serum. Curr. Biol.* **31**, 2674–2681 (2021).

Domínguez-Rodrigo, M. & Organista, E. Natural background bone assemblages and their ravaging stages in Olduvai Bed I in *Deconstructing Olduvai: A Taphonomic Study of the Bed I Sites* (eds. Domínguez-Rodrigo, M., Barba, R. & Egeland, C.P.) 201–215 (2007).

Emerson, S.B. & Radinsky, L. Functional analysis of sabertooth cranial morphology. *Paleobiology* **6**, 295–312 (1980).

Espigares, M.P. *Análisis y modelización del contexto sedimentario y los atributos tafonómicos de los yacimientos pleistocénicos del borde nororiental de la cuenca de Guadix-Baza.* Ph. D. dissertation thesis, University of Granada, Spain (2010).

Espigares, M.P., Martínez-Navarro, B., Palmqvist, P., Ros-Montoya, S., Toro, I., Agustí, J. & Sala, R. *Homo* vs. *Pachycrocuta:* Earliest evidence of competition for an elephant carcass between scavengers at Fuente Nueva-3 (Orce, Spain). *Quat. Int.* **295**, 113–125 (2013).

Espigares, M.P., Palmqvist, P., Guerra-Merchán, A., Ros-Montoya, S., García-Aguilar, J.M., Rodríguez-Gómez, G., Serrano, F.J. & Martínez-Navarro, B. The earliest cut marks of Europe: a discussion on hominin subsistence patterns in the Orce sites (Baza basin, SE Spain). *Sci. Rep.* **9**, 15408 (2019). https://doi.org/10.1038/s41598-019-51957-5

Fick, S.E. & Hijmans, R.J. Worldclim 2: New 1-km spatial resolution climate surfaces for global land areas. *Inter. J. Climatol.* **37**, 4302–4315 (2017).

Figueirido, B., Lautenschlager, S., Pérez-Ramos, A. & Van Valkenburgh, B. Distinct predatory behaviors in scimitar- and dirk-toothed sabertooth cats. *Curr. Biol.* **28**, 3260–3266 (2018).

Figueirido, B., MacLeod, N., Krieger, J., de Renzi, M., Pérez-Claros, J.A. & Palmqvist, P. Constraint and adaptation in the evolution of carnivoran skull shape. *Paleobiology* **37**, 490–518 (2011).

Fortelius, M., Mazza, P. & Sala. B. *Stephanorhinus* (Mammalia: Rhinocerotidae) of the western European Pleistocene, with a revision of *S. etruscus* (Falconer, 1868). *Palaeontograph. Italica* **80,** 63–155 (1993)

Foucault, A. & Mélières, F. Palaeoclimatic cyclicity in central Mediterranean Pliocene sediments: the mineralogical signal. *Palaeogeogr. Palaeoclimatol. Palaeoecol.* **158**, 311–323 (2000).

Fourvel, J. B., Magniez, P., Moigne, A. M., Testu, A., Joris, A., Lamglait, B., Vaccaro, C. & Fosse, P. Wild dogs and their relatives: implication of experimental feedings in their taphonomical identification. *Quaternaire* **29**, 21–29 (2018).

Freytet, P. Le Danien (Dano-Montien) des petites Pyrénées et du Plantaurel: Etude pétrogràfique et palèogèographique des facies lacustres. *Geol. Mediterr.* **2**, 159–178 (1975).

Garcés, M., Agustí, J. & Parés, J.M. Late Pliocene continental magnetocronology in the Guadix-Baza basin (Betic Ranges, Spain). *Earth Planet. Sci. Lett.* **146**, 677–687 (1997).

García-Aguilar, J.M., Guerra-Merchán, A., Serrano, F., Flores-Moya, A., Delgado-Huertas, A., Espigares, M.P., Ros-Montoya, S., Martínez-Navarro, B. & Palmqvist, P. A reassessment of the evidence for hydrothermal activity in the Neogene-Quaternary lacustrine environments of the Baza basin (Betic Cordillera, SE Spain) and its paleoecological implications. *Quat. Sci. Rev.* **112**, 226–235 (2015).

García-Aguilar, J.M., Guerra-Merchán, A., Serrano, F. & Palmqvist P. Ciclicidad sedimentaria en depósitos lacustres evaporíticos tipo playa-lake del Pleistoceno inferior en la cuenca de Guadix-Baza (Cordillera Bética, España). *Bol. Geol. Min.* **124**, 239–251 (2013).

García-Aguilar, J.M. & Martín, J.M. Late Neogene to recent continental history and evolution of Guadix-Baza basin (SE Spain). *Rev. Soc. Geol.* **13**, 65–77 (2000).

García-Aguilar, J.M. & Palmqvist, P. A model of lacustrine sedimentation for the Early Pleistocene deposits of Guadix-Baza basin (southeast Spain). *Quat. Int.* **243**, 3–15.

García-Aguilar, J.M., Guerra-Merchán, A., Serrano, F., Palmqvist, P., Flores-Moya, A. & Martínez-Navarro, B. Hydrothermal activity and its paleoecological implications in the latest Miocene to Middle Pleistocene lacustrine environments of the Baza Basin (Betic Cordillera, SE Spain). *Quat. Sci. Rev.* **96**, 204–221 (2014).

Gibert, L., Ortí, F. & Rosell, L. Plio-Pleistocene lacustrine evaporites of the Baza Basin (Bétic Chain, SE Spain). *Sediment. Geol.* **200**, 89–116 (2007).

Gonyea, W.J. Behavioral implications of saber-toothed felid morphology. *Paleobiology* **2**, 332–342 (1976).

Gorman, M. L., Mills, M. G. L., Raath, J. P. & Speakman, J. R. High hunting costs make African wild dogs vulnerable to kleptoparasitism by hyaenas. *Nature* **391**, 479–481 (1998).

Guérin, C. Les rhinocéros (Mammalia, Perissodactyla) du Miocèneterminal au Pléistocène supérieur en Europe occidentale, comparaison avec les espèces actuelles. *Doc. Lab. Géol. Fac. Sci,* Lyon **79**, 3–1183 (1980).

Guerra-Merchán, A. Sobre la conexión entre la Depresión de Guadix-Baza y el Corredor del Almanzora. (Cordilleras Béticas, Andalucía oriental). *Geogaceta* **8**, 97–99 (1990).

Guerra-Merchán, A. *La cuenca neógena del corredor del Almanzora.* Ph.D. dissertation thesis, University of Grenade. (Málaga Ediciones Edinford, 1993).

Hartstone-Rose, A., Werdelin, L., De Ruiter, D. J., Berger, L. R., & Churchill, S. E. The Plio-Pleistocene ancestor of wild dogs, *Lycaon sekowei* n. sp. *J. Paleont.* **84**, 299–308 (2010).

Hatton, I.A., McCann, K.S., Fryxell, J.M., Davies, T.J., Smerlak, M., Sinclair, A.R.E. & Loreau, M. The predator-prey power law: Biomass scaling across terrestrial and aquatic biomes. *Science* **349**, 6252 (2015). Doi: 10.1126/science.aac6284

Hayward, M. W., O'Brien, J., Hofmeyr, M. & Kerley, G. I. H. Prey preferences of the African wild dog *Lycaon pictus* (Canidae: Carnivora): Ecological requirements for conservation. *J. Mammal.* **87**, 1122–1131 (2006).

Haywood, A.M., & Valdes, P.J. Modelling Pliocene warmth: contribution of atmosphere, oceans and cryosphere. *Earth Planet. Sci. Lett.* **218,** 363–377 (2004).

Hubel, T.Y., Myatt, J.P., Jordan, N.R., Dewhirst, O.P., Weldon McNutt, J. & Wilson A.M. Energy cost and return for hunting in African wild dogs and cheetahs. *Nat. Comm.* **7** 11034 (2016). https://doi.org/10.1038/ncomms11034

Hüsing S.K., Oms, O., Agustí, J., Garcés, M., Kouwenhoven, T.J., Krijgsman, W. & Zachariasse, W.J. On the late Miocene closure of the Mediterranean–Atlantic gateway through the Guadix basin (southern Spain). *Palaeogeogr. Palaeoclimatol. Palaeoecol.* **291**, 167–179 (2010).

Janis, C.M. The evolutionary strategy of the Equidae and the origins of rumen and caecal fermentation. *Evolution* **30**, 757–774 (1976).

Janis, C.M., Gordon, I.J. & Illius, A.W. Modelling equid/ruminant competition in the fossil record. *Hist. Biol.* **8**, 15–29 (1984).

Kerbis Peterhans, J. C. & Gnoske, T. P. The science of 'man-eating' among lions *Panthera leo* with a reconstruction of the Natural History of the 'man-eaters of Tsavo'. *J. East Afr. Nat. Hist.* **90**, 1–40 (2001).

Kerbis Peterhans, J. C., Kusimba, C. M., Gnoske, T. P., Andanje, S. & Patterson, B. D. Maneaters of Tsavo. *Nat. Hist.* **107**, 12–14 (1998).

Kuhn, B. F, Wiesel, I. & Skinner, J. D. Diet of brown hyaenas (*Parahyaena brunnea*) on the Namibian coast. *Trans. R. Soc. S. Afr.* **63**, 150–159 (2008).

Lansing, S. W., Cooper, S. M., Boydston, E. E. & Holekamp, K. E. Taphonomic and zooarchaeological implications of spotted hyena (*Crocuta crocuta*) bone accumulations in Kenya: a modern behavioral ecological approach. *Paleobiology* **35**, 289–309 (2009).

Lavrov, A. V., Gimranov, D. O., Madurell-Malapeira, J. & Lopatin, A. V. *Megantereon adroveri* from the Early Pleistocene of Taurida Cave, Crimea, and the European lineage of dirk-toothed cats. *J. Mamm. Evol.* https://doi.org/10.1007/s10914-021-09578-1 (2021).

Lewis, M.E. Carnivoran paleoguilds of Africa: implications for hominid food procurement strategies. *J. Hum. Evol.* **32**, 257–288 (1997).

Luzón, C., Yravedra, J., Courtenay, L.A., Saarinen, J., Blain, H.A., DeMiguel, D., Viranta, S., Azanza, B., Rodríguez-Alba, J.J., Herranz-Rodrígo, D., Serrano-Ramos, A., Solano, J.A., Oms, O., Agustí, J., Fortelius, M. & Jiménez-Arenas, J.M. Taphonomical and spatial analyses from the Early Pleistocene site of Venta Micena 4 (Orce, Guadix-Baza, Basin, southern Spain). Sci. Rep. **11**, 13977 (2021). https://doi.org/10.1038/s41598-021-93261-1

Maldonado-Garrido, E., Piñero, P. & Agustí J. A catalogue of the vertebrate fossil record from the Guadix-Baza Basin (SE Spain). *Span. J. Paleontol.* **32**, 207–236 (2017).

Marean, C.W. & Ehrhardt, C.L. Paleoanthropological and paleoecological implications of the taphonomy of a sabertooth's den. *J. Hum. Evol.* **29**, 515–547 (1995).

Martin, L.D., Babiarz, J.P., Naples, V.L. & Hearst, J. Three ways to be a saber-toothed cat. *Naturwissenschaften* **87**, 41–44 (2000).

Martín-González, J.A., Rodríguez-Gómez, G. & Palmqvist, P. Survival profiles from linear models versus Weibull models: Estimating stable and stationary population structures for Pleistocene large mammals. *J. Archaeol. Sci. Rep.* **25**, 370–386 (2019).

Martín-Serra, A., Figueirido, B. & Palmqvist P. Non-decoupled morphological evolution of the fore- and hindlimb of sabretooth predators. *J. Anat.* **231**, 532–542 (2017).

Martín-Suárez, E. Sucesiones de micromamíferos en la Depresión de Guadix-Baza (Granada, España). PhD dissertation theis (University of Granada, 1988).

Martínez-García, B., Ordiales, A., Pérez, A., Muñoz, A., Luzón, A. & Murelaga, X. Palaeoenvironmental evolution of the Holocene record of the Añavieja lake (Soria, NE Iberian Peninsula) based on the study of the ostracod assemblages. *Geogaceta* **62**, 43–46 (2017).

Martínez-García, B., Suárez-Hernando, O., Hernández, J.M., Suárez-Bilbao, A. & Murelaga, X. Palaeoenvironmental analysis based on alluvial ostracod assemblages of the Cenicero section (lower Miocene, NW Ebro Basin). *Estud. Geol.* **71**, e024 (2015). http://hdl.handle.net/10810/18207

Martínez-Navarro, B. Revisión sistemática y estudio cuantitativo de la fauna de macromamíferos del yacimiento de Venta Micena (Orce, Granada). Ph.D. thesis dissertation (Universidad Autónoma de Barcelona, 1991).

Martínez-Navarro, B. Hippos, pigs, bovids, sabertoothed tigers, monkeys and hominids dispersals during late Pliocene and Early Pleistocene times through the levantine corridor in *Human Paleoecology in the Levantine Corridor* (eds. Goren-Inbar, N. & Speth, J. D.) 37-51 (Oxbow Books, 2004).

Martínez-Navarro, B. Early Pleistocene faunas of Eurasia and hominid dispersals in *The first hominin colonization of Eurasia. Contributions from the Second Stony Brook Human Evolution Symposium and Workshop* (eds. Fleagle, J. G., Shea, J. J., Grine, F.E., Baden, A.L. & Leakey, R. E.) 207–224 (New York Springer, 2010).

Martínez-Navarro, B. Oldowan scavengers vs. Acheulian hunters: What does the faunal record say? *Global J. Archeol. Anthropol.* **6**, 555679 (2018). Doi: 10.19080/GJAA.2018.06.555679

Martínez-Navarro, B., Espigares, M.-P., Pastó, I., Ros-Montoya, S.& Palmqvist, P. Europe: Early Homo Fossil Records in *Encyclopedia of Global Archaeology* (ed, Smith, C.) 2561–2570 (New York Springer, 2014).

Martínez-Navarro, B. & Palmqvist, P. Presence of the African Machairodont *Megantereon white*i (Broom, 1937) (Felidae, Carnivora, mammalia) in the lower pleistocene site of Venta Micena (Orce, Granada, Spain), with some considerations on the origin, evolution and dispersal of the genus. *J. Archaeol. Sci.* **22**, 569–582 (1995).

Martínez-Navarro, B. & Palmqvist, P. Presence of the African saber-toothed felid *Megantereon whitei* (Broom, 1937) (Mammalia, Carnivora, Machairodontinae) in Apollonia-1 (Mygdonia basin, Macedonia, Greece). *J. Archaeol. Sci.* **23**, 869–872 (1996).

Martínez-Navarro, B., Palmqvist, P., Madurell-Malapeira, J., Ros-Montoya, S., Espigares, M.-P., Torregrosa, V. & Pérez-Claros, J. A. La fauna de grandes mamíferos de Fuente Nueva-3 y Barranco León-5: estado de la cuestión in *Ocupaciones humanas en el Pleistoceno inferior y medio de la cuenca de Guadix-Baza* (eds. Toro, I, Martínez-Navarro, B. & Agustí, J.) 197–236 (Junta de Andalucía, 2010).

Martínez-Navarro, B. & Rook, L. Gradual evolution in the African hunting dog lineage. Systematic implications. *C. R. Palevol* **2**, 695–702 (2003).

Martínez-Navarro, B., Ros-Montoya, S., Espigares, M.P., Madurell-Malapeira, J. & Palmqvist, P. Los mamíferos del Plioceno y Pleistoceno de la Península Ibérica. *Revista PH* **94**, 206–249 (2018).

Martínez-Navarro, B., Ros-Montoya, S., Espigares, M.P. & Palmqvist, P. Presence of Asian origin Bovini, *Hemibos* sp. aff. *H. gracilis* and *Bison* sp., at the Early Pleistocene site of Venta Micena (Orce, Spain). *Quat. Int.* **243**, 54–60 (2011).

McHenry, C., Wroe, S., Clausen, P.D., Moreno, K. & Cunningham, E. Supermodeled sabercat, predatory behavior in *Smilodon fatalis* revealed by high-resolution 3D computer simulation. *Proc. Natl. Acad. Sci.* USA **104**, 16010–16015 (2007).

Meachen-Samuels, J.A. Morphological convergence of the prey-killing arsenal of sabertooth predators. *Paleobiology* **38**, 715–728 (2012).

Meachen-Samuels, J.A. & Van Valkenburgh, B. Radiographs reveal exceptional forelimb strength in the sabertooth cat, *Smilodon fatalis. PLoS One* **5**, e11412 (2010). doi:10.1371/journal.pone.0011412

Medin, T., Martínez-Navarro, B., Madurell-Malapeira, J. et al. The bears from Dmanisi and the first dispersal of early Homo out of Africa. *Sci. Rep.* **9**, 17752 (2019). https://doi.org/10.1038/s41598-019-54138-6

Medin, T., Martínez-Navarro, B., Rivals, F., Madurell-Malapiera, J., Ros-Montoya, S., Espigares, M., Figueirido, B., Rook, L. & Palmqvist, P. Late Villafranchian Ursus etruscus and other large carnivorans from the Orce sites (Guadix-Baza basin, Andalusia, southern Spain): Taxonomy, biochronology, paleobiology, and ecogeographical context. *Quat. Int.* **431**, 20–41 (2017).

Medina-Cascales, I., Martin-Rojas, I., García-Tortosa, F.J., Peláez, J.A. & Alfaro, P. Geometry and kinematics of the Baza Fault (central Betic Cordillera, South Spain): insights into its seismic potential. *Geol. Acta* **18.11**, 1–25 (2020).

Mendoza, M., Janis, C.M. & Palmqvist P. Ecological patterns in the trophic-size structure of large mammal communities: a 'taxon-free' characterization. *Evol. Ecol. Res.* **7**, 505–530 (2005).

Moullé, P.-E., Echassoux, A. & Martínez-Navarro, B. Ammotragus europaeus: une nouvelle espèce de Caprini (Bovidae, Mammalia) du Pléistocène inférieur à la grotte duVallonnet (France). *C. R. Palevol* **3**, 663–673 (2004).

Moyà-Solà, S. Los bóvidos (Artiodactyla, Mammalia) del yacimiento del Pleistoceno inferior de Venta Micena (Orce, Granada, España). *Paleontología i Evolució Mem. esp.* **1**, 181–236 (1987).

Moyà-Solà, S., Agustí, J., Gibert, J. & Pons-Moyà, J. El yacimiento cuaternario de Venta Micena (España) y su importancia dentro de las asociaciones faunísticas del Pleistoceno inferior europeo. *Paleontologia i Evolució* **16**, 39–53 (1981).

Murphy, D. M. & Wilkinson, B. M. Carbonate deposition and facies distribution in a central Michigan marl lake. *Sedimentology* **27**, 123–135 (1980).

Palmqvist, P. On the presence of *Megantereon whitei* at the South Turkwel hominid site, northern Kenya. *J. Paleont.* **76**, 928–930 (2002).

Palmqvist P. & Arribas A. Taphonomic decoding of the paleobiological information locked in a lower Pleistocene assemblage of large mammals. *Paleobiology* **27**, 512–530 (2001).

Palmqvist, P., Arribas, A. & Martínez-Navarro, B. Ecomorphological study of large canids from the lower Pleistocene of southeastern Spain. *Lethaia* **32**, 75–88 (1999).

Palmqvist, P., Duval, M., Diéguez, A., Ros-Montoya, S. & Espigares, M.P. On the fallacy of using orthogenetic models of rectilinear change in arvicolid teeth for estimating the age of the first human settlements in Western Europe. *Hist. Biol.* **28**, 734–752 (2016).

Palmqvist, P., Gröcke, D.R., Arribas, A., Fariña, R. Paleoecological reconstruction of a lower Pleistocene large mammals community using biogeochemical ($\delta^{13}C$, $\delta^{15}N$, $\delta^{18}O$, Sr: Zn) and ecomorphological approaches. *Paleobiology* **29**, 205–229 (2003).

Palmqvist, P., Martínez-Navarro, B. & Arribas, A. Prey selection by terrestrial carnivores in a lower Pleistocene paleocommunity. *Paleobiology* **22**, 514–534 (1996).

Palmqvist, P., Martínez-Navarro, B., Pérez-Claros, J.A., Torregrosa, V., Figueirido, B., Jiménez-Arenas, J.M., Espigares, M.P., Ros-Montoya, S. & de Renzi, M. The giant hyena Pachycrocuta brevirostris: modelling the bone-cracking behavior of an extinct carnivore. *Quat. Int.* **243**, 61–79 (2011).

Palmqvist, P., Martínez-Navarro, B., Toro, I., Espigares, M.P., Ros-Montoya, S., Torregrosa, V. & Pérez-Claros, J.A. Réévaluation de la présence humaine au Pléistocène inférieur dans le Sud de l'Espagne. *L'Anthropologie* **109**, 411–450 (2005).

Palmqvist, P., Pérez-Claros, J.A, Gröcke, D.R. & Janis, C.M. Tracing the ecophysiology of ungulates and predator-prey relationships in an early Pleistocene large mammal community. *Palaeogeogr., Palaeoclimatol., Palaeoecol.* **266**, 95–111 (2008a).

Palmqvist, P., Pérez-Claros, J.A., Janis, C.M., Figueirido, B., Torregrosa, V. & Gröcke, D.R. Biogeochemical and ecomorphological inferences on prey selection and resource partitioning among mammalian carnivores in an early Pleistocene community. *Palaios* **23**, 724–737 (2008b).

Palmqvist, P., Torregrosa, V., Pérez-Claros, J.A., Martínez-Navarro, B. & Turner, A. A re-evaluation of the diversity of *Megantereon* (Mammalia, Carnivora, Machairodontinae) and the problem of species identification in extinct carnivores. *J. Vert. Paleont.* **27**, 160–175 (2007).

Pandolfi, L., Bartolini-Lucenti, S., Cirilli, O., Bukhsianidze, M., Lordkipanidze, D. & Rook, L. Paleoecology, biochronology, and paleobiogeography of Eurasian Rhinocerotidae during the Early Pleistocene: The contribution of the fossil material from Dmanisi (Georgia, Southern Caucasus). *J. Hum. Evol.* **156**, 103013 (2021). https://doi.org/10.1016/j.jhevol.2021.103013

Pandolfi, L., Cerdeño, E., Codrea, V. & Kotsakis, T. Biogeography and chronology of the Eurasian extinct rhinoceros *Stephanorhinus etruscus* (Mammalia, Rhinocerotidae). *C. R. Palevol* **16** (7), 762–773 (2017).

Patterson, J. H. The man-eating lions of Tsavo. *Field Mus. Nat. Hist. Chicago Leaflet* **7**, 89–128 (1925).

Pérez-Claros, J. A. & Coca-Ortega, C. Canines and carnassials as indicators of sociality in durophagous hyaenids: analyzing the past to understand the present. *PeerJ*, **8**, e10541 (2020). Doi: 10.7717/peerj.10541

Périquet, S., Mapendere, C., Revilla, E., Banda, J., Macdonald, D. W., Loveridge, A. J. & Fritz, H. A potential role for interference competition with lions in den selection and attendance by spotted hyaenas. *Mamm. Biol.* **81**, 227–234 (2016).

Piñero, P. & Agustí, J. Rodents from Botardo-D and the Miocene-Pliocene transition in the Guadix-Baza Basin (Granada, Spain). *Palaeodivers. Paleoenvir.* **100**, 903–920 (2020).

Pons-Moya, J. Los carnívoros (Mammalia) de Venta Micena (Granada, España). *Paleontologia i Evolució Mem. Espec.* **1**, 10–127 (1987).

Rawn-Schatzinger, V. The scimitar cat Homotherium serum Cope: osteology, functional morphology, and predatory behaviour. *Illinois State Museum Reports of Investigations* **47**, 1–80 (1992).

Ripple, W.J. & Van Valkenburgh, B. Linking top-down forces to the Pleistocene megafaunal extinctions. *BioScience* **60**, 516–526 (2010).

Rodríguez-Alba, J.J., Linares-Matás, G. & Yravedra, J. First assessments of the taphonomic behaviour of jaguar (*Panthera onca*). *Quat. Int.* **517**, 88–96 (2019).

Rodríguez-Gómez, G., Mateos, A., Martín-González, J.A., Blasco, R., Rosell, J., Rodríguez, J. Discontinuity of human presence at Atapuerca during the early Middle Pleistocene: A matter of ecological competition? *PLoS ONE* **9** (7), e101938 (2014a). doi:10.1371/journal.pone.0101938

Rodríguez-Gómez, G., Martín-González, J.A., Goikoetxea, I., Mateos, A. & Rodríguez, J. A new mathematical approach to model trophic dynamics of mammalian palaeocommunities. The case of Atapuerca-TD6 in *Mathematics of Planet Earth, 15th Annual Conference of the International Association for Mathematical Geosciences.* (eds. Pardo-Igúzquiza, E., Guardiola-Albert, C., Heredia, J., Moreno-Merino, L., Durán, J.J. & Vargas-Guzmán, J.A.) 739–745 Lectures Notes in Earth System Sciences (Madrid Springer, 2014b).

Rodríguez-Gómez, G., Palmqvist, P., Martínez-Navarro, B., Martín-González, J.A. & Bermúdez de Castro, J.M. Mean body size estimation in large mammals and the computation of biomass in past ecosystems: An application to the Pleistocene sites of Orce and Sierra de Atapuerca (Spain). *C.R. Palevol,* in press (2022).

Rodríguez-Gómez, G., Palmqvist, P., Rodríguez, J., Mateos, A., Martín-González, J.A., Espigares, M.P., Ros-Montoya, S. & Martínez-Navarro, B. On the ecological context of the earliest human settlements in Europe: Resource availability and competition intensity in the carnivore guild of Barranco León-D and Fuente Nueva-3 (Orce, Baza Basin, SE Spain). *Quat. Sci. Rev.* **134**, 69–83 (2016).

Rodríguez-Gomez, G., Palmqvist, P., Ros-Montoya, S. Espigares, M.P. & Martinez-Navarro, B. Resource availability and competition intensity in the carnivore guild of the Early Pleistocene site of Venta Micena (Orce, Baza Basin, SE Spain). *Quat. Sci. Rev.***164**,154-167 (2017).

Rodríguez-Gómez, G., Rodríguez, J., Martín-González, J.A., Goikoetxea, I. & Mateos, A. Modeling trophic resource availability for the first human settlers of Europe: The case of Atapuerca-TD6. *J. Hum. Evol.* **64**, 645–657 (2013).

Rook, L., Martínez-Navarro, B. & Howell, F.C. Occurrence of Theropithecus sp. in the Late Villafranchian of Southern Italy and implication for Early Pleistocene “out of Africa” dispersals. *J. Hum. Evol.* **47**, 267–277 (2004).

Ros-Montoya, S., Madurell-Malapeira, J., Martínez-Navarro, B., Espigares, M.-P. & Palmqvist, P. Late Villafranchian *Mammuthus meridionalis* (Nesti, 1825) from the Iberian Peninsula: Dentognathic remains from Incarcal-I (Crespià, Girona) and Venta Micena (Orce, Granada). *Quat. Int.* **276**, 17–22 (2012).

Ros-Montoya, S., Martínez-Navarro, B., Espigares, M.P., Guerra-Merchán, A., García-Aguilar, J.M., Piñero, P., Rodríguez-Rueda, A., Agustí, J., Oms, O. & Palmqvist, P. A new Ruscinian site in Europe: Baza-1 (Baza Basin, Andalusia, Spain). *C. R. Palevol* **16**, 746–761 (2017).

Saarinen, J., Oksanen, O., Žliobaitė, I., Fortelius, M., DeMiguel, D., Azanza, B., Bocherens, H., Luzón, C., Solano-García, J., Yravedra, J., Courtenay, L.A., Blain, H.-A., Sánchez-Bandera, C., Serrano-Ramos, A., Rodriguez-Alba, J. J., Viranta, S., Barsky, D., Tallavaara, M., Oms, O., Agustí, J., Ochando, J., Carrión, J. S. & Jiménez-Arenas, J. M. Pliocene to Middle Pleistocene climate history in the Guadix-Baza Basin, and the

environmental conditions of early *Homo* dispersal in Europe. *Quat. Sci. Rev.* **268**, 107132 (2021). https://doi.org/10.1016/j.quascirev.2021.107132

Salesa, M., Antón, M., Turner, A. & Morales, J. Functional anatomy of the forelimb in *Promegantereon ogygia* (Felidae, Machairodontinae, Smilodontini) from the Late Miocene of Spain and the origins of the sabre-toothed felid model. *J. Anat.* **216**, 381–396 (2010).

Santafé-Llopis, J.V. & Casanovas-Cladellas, M.L. Dicerorhinus etruscus brachycephalus (Mammalia, Perissodactyla) de los yacimientos pleistocénicos de la cuenca de Guadix-Baza (Venta Micena y Huéscar) (granada, España). *Paleontologia i Evolució Mem. esp.,* **1**, 237–254 (1987).

Sanz, M. & Daura, J. Taphonomic analysis of an ungulate-dominated accumulation at the Pleistocene Cova del Rinoceront site near Barcelona, Spain (northeastern Iberian Peninsula). *Palaeogeogr. Palaeoclimatol. Palaeoecol.* **498**, 24–38 (2018).

Schaller, G. B. Hunting behaviour of the cheetah in the Serengeti National Park, Tanzania. *Afr. J. Ecol.* **6**, 95–100 (1968).

Schaller, G. B. *The Serengeti lion*. (Chicago University of Chicago Press, 1972).

Schubert, B. W., Ungar, P. S. & DeSantis, L. R. G. Carnassial microwear and dietary behaviour in large carnivorans. *J. Zool.* **280**, 257–263 (2010).

Scott, G.R., Gibert, Ll. & Gibert, J. Magnetostratigraphy of the Orce region (Baza Basin), SE Spain: New chronologies for Early Pleistocene faunas and hominid occupation sites. *Quat. Sci. Rev.* **26**, 415–435 (2007).

Sealy, J. C., van der Merwe, N. J., Lee Thorp, J.A., Lanham, J.L. Nitrogen isotopic ecology in southern Africa: Implications for environmental and dietary tracing. *Geochim. Cosmochim. Acta*, **51, 10,** 2707–2717 (1987).

Skinner, J. D., Henschel, J. R. & Van Jaarsveld, A. S. Article Bone-collecting habits of spotted hyaenas *Crocuta crocuta* in the Kruger National Park. *Afr. Zool.* **21**, 303–308 (1986).

Skinner, J.D. & Smithers, R.H.N. *The Mammals of the Southern African Subregion*. (Cambridge University Press, 1990).

Slater, G.J. & Van Valkenburgh B. Long in the tooth: evolution of sabertooth cat cranial shape. *Paleobiology* **34**, 403–419 (2008).

Soria, F.J., López-Garrido, A.C. & Vera, J.A. Análisis estratigráfico y sedimentológico de los depósitos neógeno-cuaternarios en el sector de Orce (depresión Guadix-Baza). *Paleontologia i Evolucio* **1**, 11–34 (1987).

Soria, J.M., Fernández, J. & Viseras, C. Late Miocene stratigraphy and palaeogeographic evolution of the intramontane Guadix Basin (Central Betic Cordillera, Spain): implications for an Atlantic–Mediterranean connection. *Palaeogeogr. Palaeoclimatol. Palaeoecol.***151**, 255–266 (1999).

Spoor, C.F.,1985. Body proportions in Hyaenidae. Anatomischer Anzeiger **160**, 215–220.

Stewart, M., Andrieux, E., Clark-Wilson, R. et al. Taphonomy of an excavated striped hyena (Hyaena hyaena) den in Arabia: implications for paleoecology and prehistory. *Archaeol. Anthropol. Sci.* **13**, 139 (2021). https://doi.org/10.1007/s12520-021-01365-6.

Turner, A. *Megantereon cultridens* from Plio-Pleistocene age deposits in Africa and Eurasia, with comments on dispersal and the possibility of a New World origin (Mammalia, Felidae, Machairodontinae). *J. Paleont.*, **61**, 1256–1268 (1987).

Turner, A. Large carnivores and earliest European hominids: changing determinants of resource availability during the lower and middle Pleistocene. *J. Hum. Evol.* **22**, 109–126 (1992).

Turner, A., Antón, M., 1996. The giant hyena, *Pachycrocuta brevirostris* (Mammalia, Carnivora, Hyaenidae). *Geobios* **29**, 455–468.

van der Meer, E., Mpofu, J., Rasmussen, G. S. & Fritz, H. Characteristics of African wild dog natal dens selected under different interspecific predation pressures. *Mamm. Biol.* **78**, 336–343 (2013).

Van Valkenburgh, B. Skeletal indicators of locomotor behavior in living and extinct carnivores. *J. Vert. Paleontol.* **7**, 162–182 (1987).

Van Valkenburgh, B. Predation in sabre-tooth cats in *Paleobiology II* (eds. Briggs, D.E.G. & Crowther, P.R.) 420–423 Blackwell (2001).

Van Valkenburgh, B. Déjà vu: the evolution of feeding morphologies in the Carnivora. *Integr. Comp. Biol.* **47**,147–163 (2007).

Van Valkenburgh, B. Costs of carnivory: tooth fracture in Pleistocene and Recent carnivorans. *Biol.J. Linnean Soc.* **96**, 68–81 (2009).

Van Valkenburgh, B., Hayward, M.W., Ripple, W.J., Meloro, C. & Roth, V.L. The impact of large terrestrial carnivores on Pleistocene ecosystems. *Proc. Natl. Acad. Sci.* USA **113**, 862–867 (2016).

Van Valkenburgh, B. & Hertel, F. Tough times at La Brea: tooth breakage in large carnivores of the late Pleistocene. *Science* **261**, 456–459 (1993).

Van Valkenburgh, B. & Ruff, C.B. Canine tooth strength and killing behaviour in large carnivores. *J. Zool.* **212**, 379–397 (1987).

Van Valkenburgh, B. V., Teaford, M. F. & Walker, A. Molar microwear and diet in large carnivores: inferences concerning diet in the sabretooth cat, *Smilodon fatalis.* J. Zool. **222**, 319–340 (1990).

Vera, J.A. Estudio estratigráfico de la Depresión de Guadix-Baza. *Bol. Geol. Min.* **81**, 429–462 (1970).

Vera, J.A., Fernández, J., López-Garrido, C.A. & Rodríguez-Fernández, J. Geología y estratigrafía de los materiales plioceno-pleistocenos del sector de Orce-Venta Micena (Prov. Granada). *Paleontologia i Evolució* **18**, 3–11 (1984).

Viseras, C., Soria, J.M., Durán, J.J., Pla, S., Garrido, G., García-García, F. & Arribas, A. A large-mammal site in a meandering fluvial context (Fonelas P-1. Late Pliocene, Guadix basin, Spain): sedimentological keys for the paleoenvironmental reconstruction. *Palaeogeogr. Palaeoclimatol. Palaeoecol.* **242**,139–168 (2006).

Watson, R. M.: Results of aerial livestock surveys of Kaputei division, Samburu district and North-eastern Province. *Statistics division, Min. of Finance and Planning, Republic of Kenya*, 111 pp. (1972).

Zrzavý, J., Duda, P., Robovský, J., Okřinová, I. & Pavelková Řičánková, V. Phylogeny of the Caninae (Carnivora): Combining morphology, behaviour, genes and fossils. *Zool.Scr.* **47**, 373–389 (2018).

Element	NISP
Cranium	27
Antler	11
Horn base	1
Maxilla	28
Hemimandible	93
Total cranial elements	160
Deciduous incisor	2
Incisor	78
Canine	17
Incisor or canine	1
Deciduous premolar	35
Premolar	68
Molar	156
Premolar or molar	69
Total isolated teeth	721
Atlas	4
Axis	3
Cervical vertebra	9
Vertebra indet.	88
Total vertebrae	104
Rib	139
Scapula	20
Pelvis	16
Flat bone	48
Humerus	46
Radius	24
Ulna	32
Femur	26
Tibia	89
Fibula	3
Long bone indet.	544
Magnum	8
Scaphoid	1
Unciform	4
Semilunar	1

Calcaneum	9
Astragalus	20
Cuboid	1
Cubonavicular	1
Cuneiform	2
Sesamoid	10
Carpal/tarsal indet.	34
Total carpal/tarsal bones	118
Metacarpal	59
Metatarsal	32
Metapodial indet.	107
First phalanx	18
Second phalanx	14
Third phalanx	18
Phalanx indet.	28
Total phalanges	78
Bone indet.	1,361
Coprolite	1
Turtle plate	12
**Total**	3,961

Table S1. Fossil elements unearthed from VM4 during the excavation seasons of the years 2005 and 2013–2015 (N = 3,961), classified according to anatomical regions. NISP: number of identifiable elements.

**Species**	**NISP**	**MNE**	**MNI (total)**	**MNI (c-j-a-s)**
Homotherium latidens	3	3	1	0-0-1-0
Megantereon whitei	2	2	1	0-0-1-0
Macairodontinae indet.	2	2		
Felidae indet. (cf. *Lynx pardinus*)	30	30	1	0-0-1-0
Felidae indet. (medium to large size)	1	1		
Pachycrocuta brevirostris	27	23	4	0-1-1-2
Lycaon lycaonoides	40	36	3	1-0-2-1
Canis orcensis	18	18	2	0-0-2-0
Vulpes alopecoides	3	3	1	0-0-1-0
Canidae indet.	98	97		
Ursus etruscus	6	6	2	0-0-2-0
Carnivora indet. (large size)	4			
Carnivora indet. (medium size)	1			
Carnivora indet. (medium to small size)	10			
Carnivora indet. (small size)	9			
Carnivora indet. (size indet.)	7			
Mammuthus meridionalis	24	19	2	1-0-1-0
Stephanorhinus hundsheimensis	33	29	2	0-0-2-0
Equus altidens	331	311	20	1-4-15-1
Hippopotamus antiquus	11	11	3	1-0-2-0
Bison sp.	27	25	4	1-1-2-0
Hemibos cf. *gracilis*	2	2	1	0-0-1-0
Bovini (large size)	40	32	3	1-0-2-0
Hemitragus albus	22	22	4	0-1-2-1
Praeovibos sp.	1	1	1	0-0-1-0
Soergelia minor	12	11	1	0-0-1-0
Bovidae indet. (medium to large size)	3			
Bovidae indet. (medium size)	1			
Bovidae indet. (small size)	1			
Bovidae indet. (size indet.)	37			
Praemegaceros cf. *verticornis*	103	90	9	2-3-3-1
Metacervoceros rhenanus	52	47	5	1-1-2-1
Cervidae indet. (cf. *Capreolus* sp.)	1	1	1	0-0-1-0

Cervidae indet. (size indet.)	76			
Ruminantia indet. (large size)	28			
Ruminantia indet. (medium to large size)	18			
Ruminantia indet. (medium size)	10			
Rumiantia indet. (medium to small size)	3			
Ruminantia indet. (small size)	4			
Rumiantia indet. (size indet.)	17			
Ungulate indet. (very large size)	4			
Ungulate indet. (large size)	78			
Ungulate indet. (medium to large size)	23			
Ungulate indet. (medium size)	8			
Ungulate indet. (size indet.)	14			
Mammal indet. (large size)	119			
Mammal indet. (medium to large size)	267			
Mammal indet. (medium size)	113			
Mammal indet. (medium to small size)	37			
Mammal indet. (small size)	18			
Mammal indet. (size indet.)	2,114			
Hyxtris major	6	6	1	0-0-1-0
Lagomorpha	4	4	1	0-0-1-0
Chelonia indet.	13		1	0-0-1-0
Ave indet. (large size)	5	5	1	0-0-1-0
Ave indet. (small size)	20	20	1	0-0-1-0
**Total**	3,961	857	75	

Table S2. Number of identifiable elements (NISP), minimal number of elements (MNE) and minimal number of individuals (MNI) of vertebrate species identified in the bone assemblage unearthed from quarry VM4 of Venta Micena during the excavation seasons of 2005 and 2013–2015. MNI estimates include calves (c), juveniles (j), adults (a) and senile individuals (s). The record of Felidae indet. (cf. *Lynx pardinus*) is represented by a complete forelimb with the bones in anatomical connection. The elements of a complete hand of *Canis mosbachensis* have not been included.

Element	Tooth-marked
Mandible	1
Vertebra	1
Rib	6
Pelvis	1
Flat bone	3
Humerus	8
Ulna	2
Femur	5
Tibia	16
Fibula	1
Long bone indet.	65
Carpals/tarsals	8
Metapodial	16
Phalanx	1
Indet. bones	43

Table S3. Tooth marked elements identified in the bone assemblage from quarry VM4 of Venta Micena unearthed during the excavation seasons of 2005 and 2013–2015. A total of 177 tooth marked bones were identified in a preliminary analysis by M. P. Espigares, but a substantial part of the remains analysed need to be restored.

Tooth mark types	N
Notches	52
Scores	84
Pits	63
Crenulated edges	5
Furrowing	3
Licking	2
Digested bones	2
Rodent marks	2
Bone flakes	10

Table S4. Types of tooth marks identified in the bone assemblage from quarry VM4 of Venta Micena unearthed during the excavation seasons of 2005 and 2013–2015. The two rodent marks identified include one made by a porcupine and other that can be attributed to a small-sized rodent.

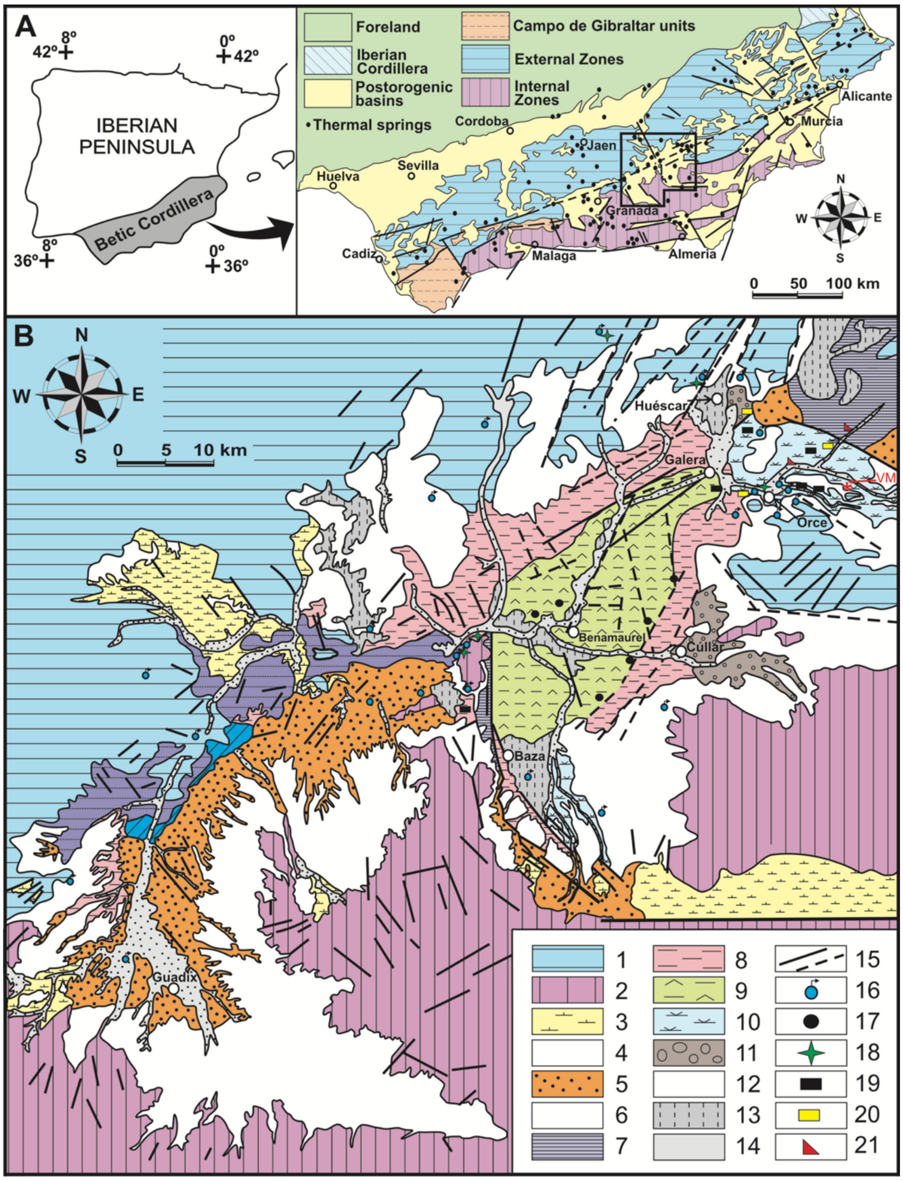

Figure S1. A: Geological context of the Guadix-Baza Depression in the Betic Cordillera, SE Spain. The box encloses the sedimentary depression. The points indicate the thermal springs (N = 122) that are nowadays active in the Betic Cordillera, with water output temperatures ranging between 18 and 60 ºC. **B:** Tectono-sedimentary map of the Guadix-Baza Depression with indication of those points that preserve geochemical, mineralogical, or lithological evidence of thermal activity during the Neogene-Quaternary. This geological cartography has been updated from García-Aguilar et al. (2014: Fig. 1) and García-Aguilar et al. (2015: Fig. 1). 1, External Zones; 2, Internal Zones; 3, Tortonian marine deposits; 4, Turolian alluvial and lacustrine deposits; 5, Plio-Pleistocene alluvial and fluvial deposits; 6, Pliocene lacustrine deposits of the Gorafe-Huélago sector; 7, Late Turolian-Ruscinian lacustrine deposits in the Baza Basin; 8, Middle Villafranchian fluvio-lacustrine deposits; 9, Middle Villafranchian lacustrine marls and evaporites; 10, Late Villafranchian lacustrine deposits; 11, Middle Pleistocene alluvial and lacustrine deposits; 12, Late Pleistocene glacis surface; 13, Holocene fluvial terrace; 14, Modern fluvial sediments; 15, Faults (solid lines; striped lines indicate those faults covered by sediments that have been inferred from aerial photographs); 16, Thermal springs nowadays active; 17, Sulphur deposits; 18, Travertine buildings; 19, Black levels; 20, Magnesium clays; 21, Silexites. The red star East of Orce indicates the position of Venta Micena. The maps depicted in this figure were generated using the software CorelDRAW Suite X4 (https://www.coreldraw.com).

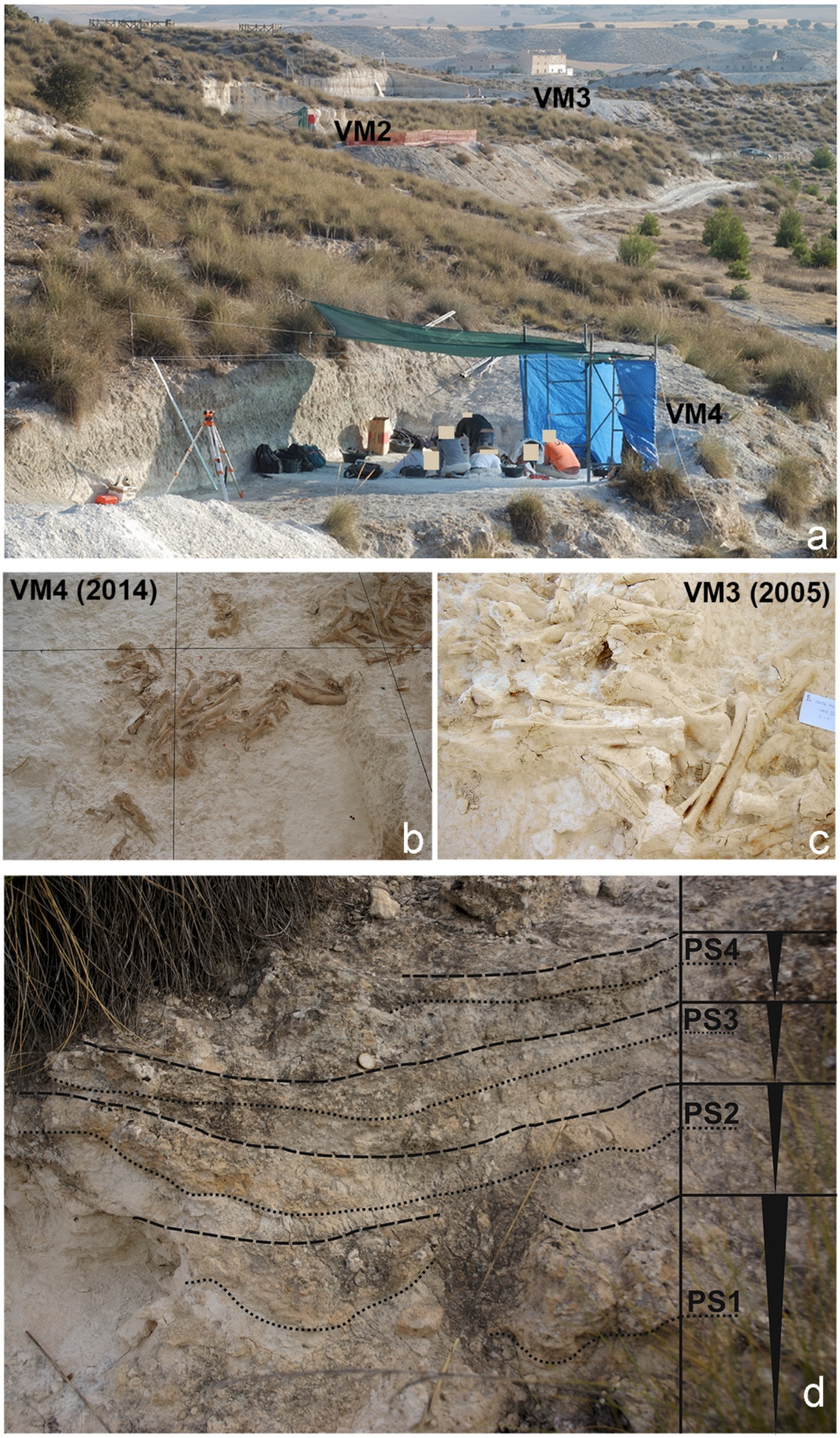

Figure S2. Panoramic view of the Venta Micena stratum (a), showing the position of three quarries discussed in the text during the excavation season of 2005, which was codirected by B. Martínez-Navarro and P. Palmqvist: VM2, VM3 (background), and VM4 (foreground). The straight-line distance between VM3 and VM4 is 350 m. Photographs of the surfaces excavated at VM4 in 2014 (b) and VM3 in 2005 (c). The photograph in (d) shows the palaeosoils (PS) identified in four highstand to lowstand cycles of somerisation in the micritic limestone level of Venta Micena (photograph taken 40 m North of VM4).

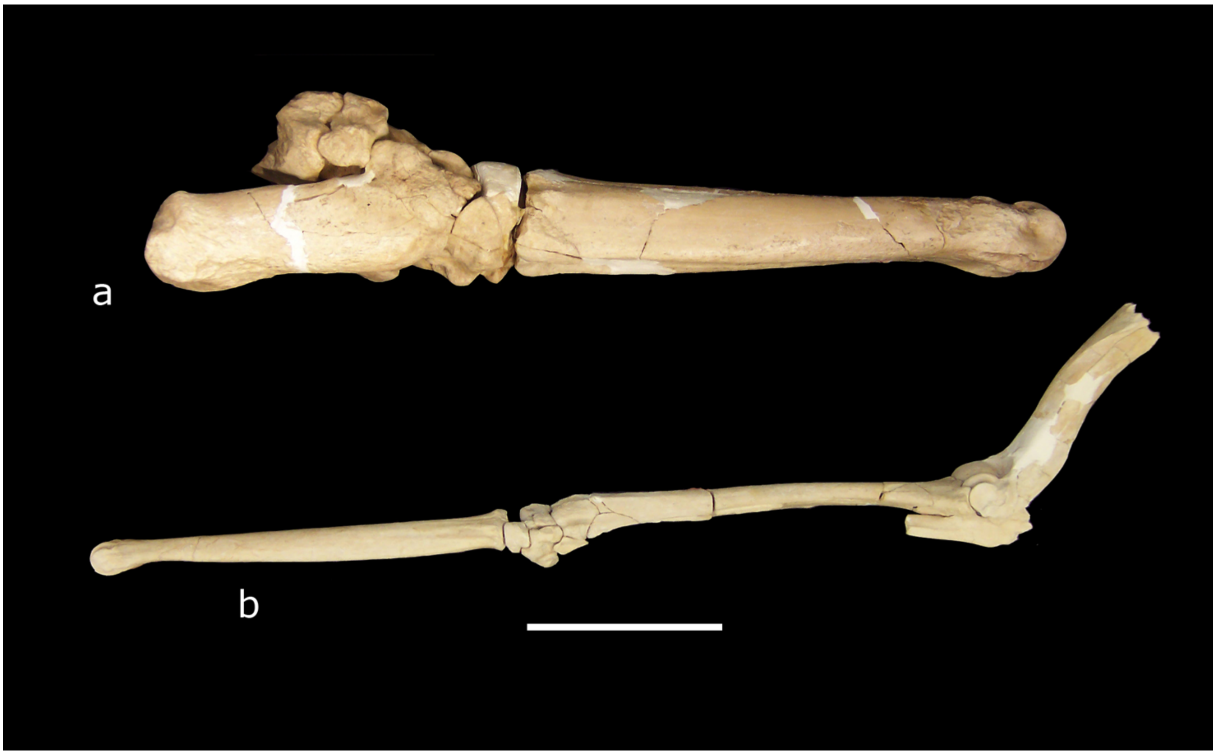

Figure S3. Bones of the same individual found associated in the excavation quarry VM3 of Venta Micena: a, elements of the tarsus y metatarsus of *Bison* sp.; b, humerus, radius, ulna, carpal, and metacarpal bones of *Metacervocerus rhenanus.* Scale bar represents 10 cm.

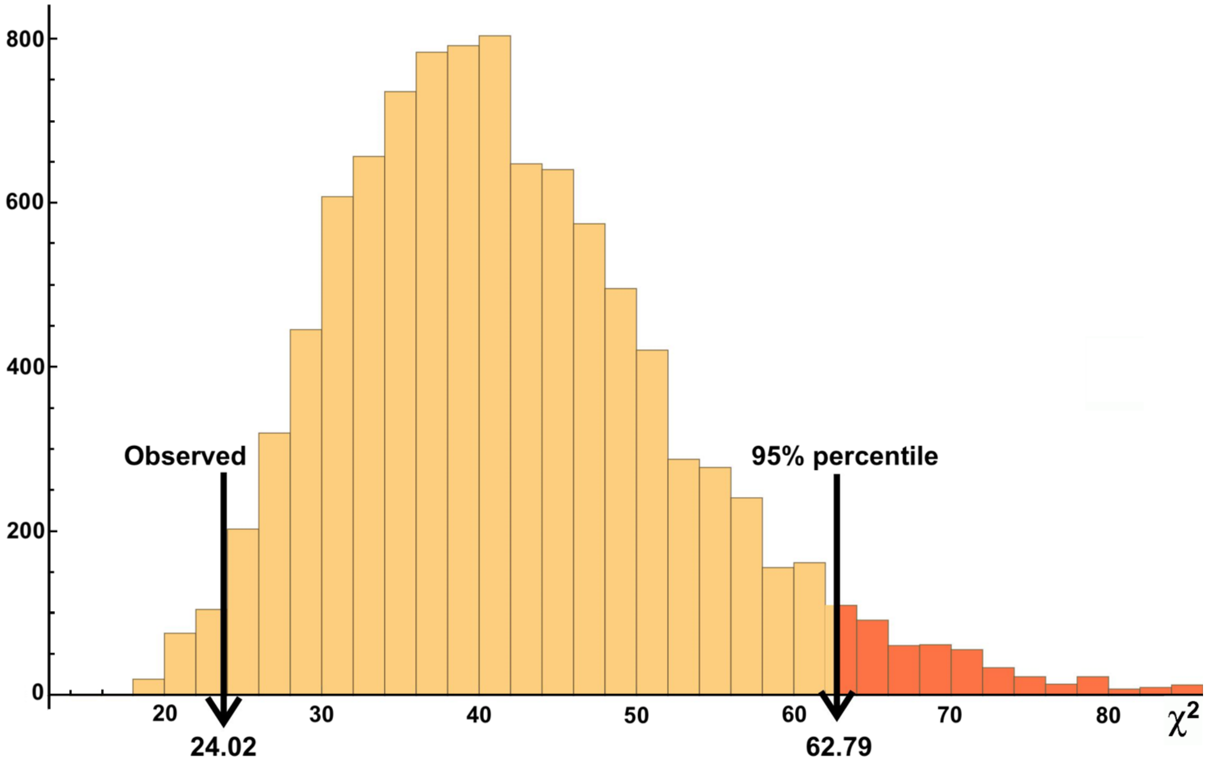

Figure S4. Results of the randomization test used to compare the abundances (MNI estimates) of the species of large mammal preserved in the assemblages of VM4 and VM3. The empirical distribution of the χ^2 statistic obtained simulating a set of random samples (n = 10^4) according to the marginal frequencies of each species is shown.

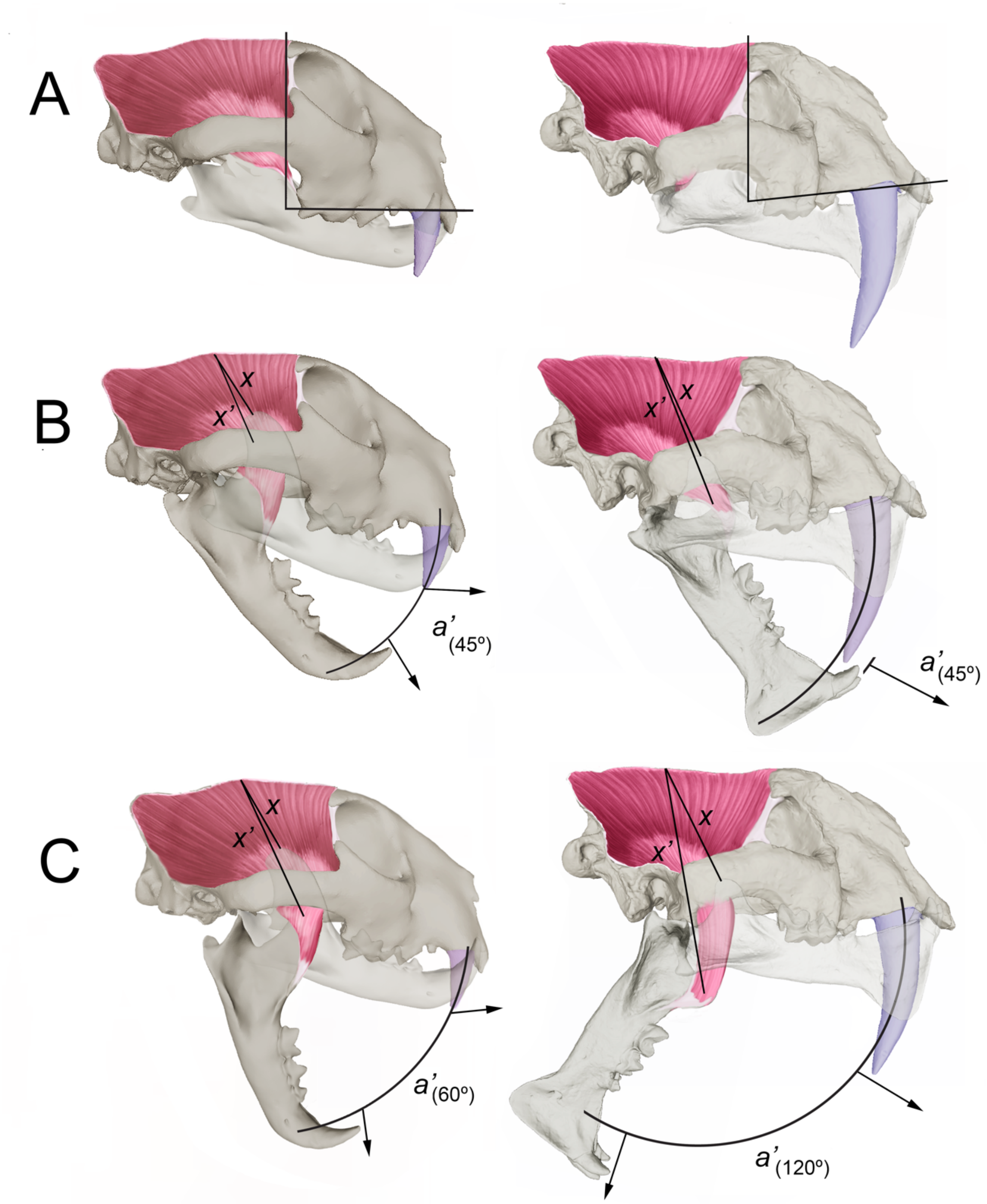

Figure S5. Comparison of the skulls of a leopard, *Panthera pardus* (left images), and *Megantereon nihowanensis* (right images) showing different angles of jaw opening. **A:** with the jaw closed and compared to the leopard skull, *Megantereon* shows a shortening of the coronoid process, an upward rotation of the palate relative to the braincase, a shorter and narrower temporal fossa, and a more vertical occiput; these changes result in a narrowing of the temporalis fibres and their more perpendicular orientation to the tooth row, which helps to avoid muscle over-stretching during wide gaping while retaining bite force at the carnassial. **B:** with a jaw gape of 45º, the stretching of the temporalis muscles (measured by the ratio between X' and X) is similar in the leopard (~30%) and *Megantereon* (~40%). **C:** with a gape of 120º, muscle stretching in the skull of *Megantereon* (~80%) is like in the leopard with a jaw gape of 60º (~85%).

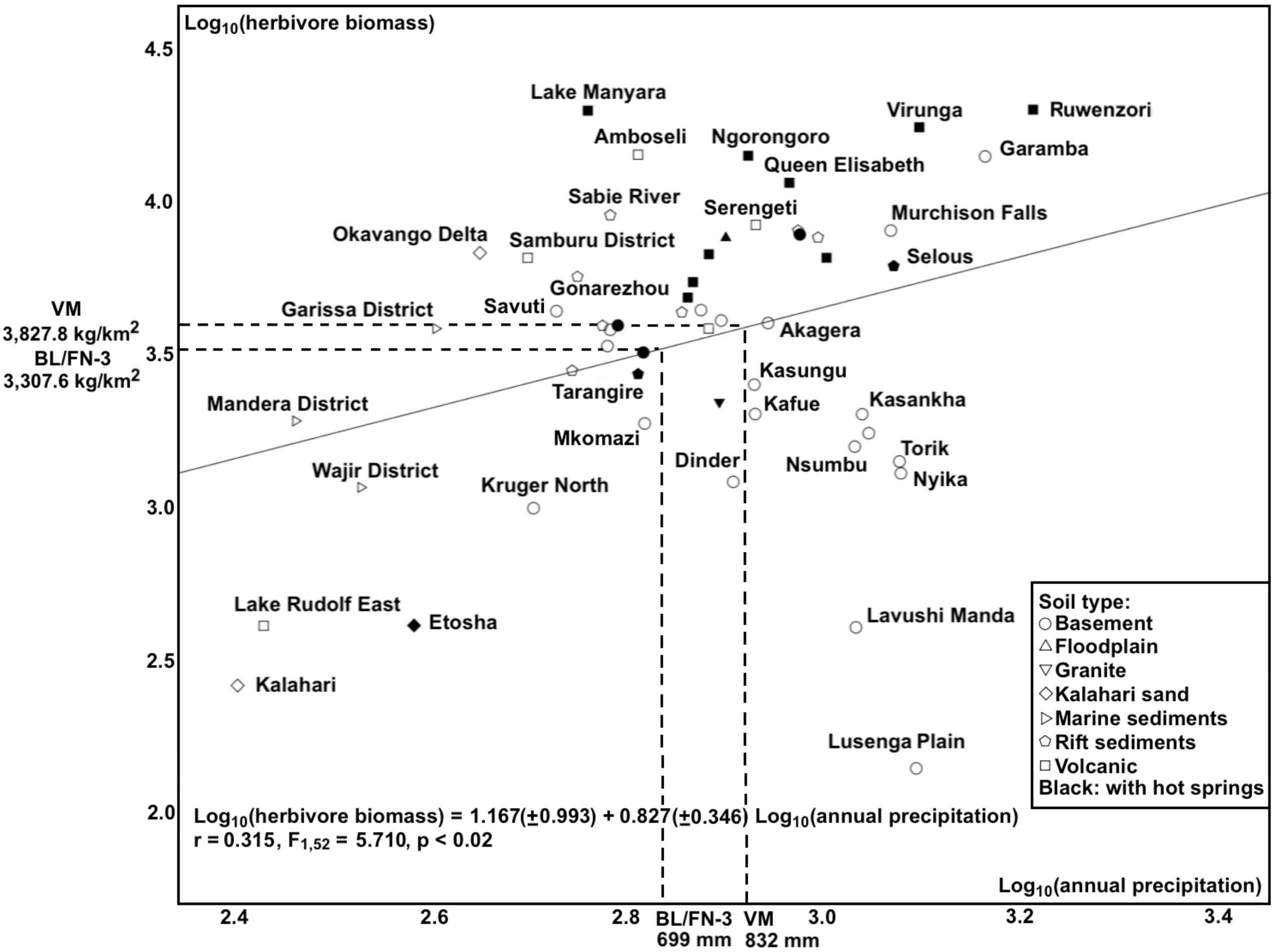

Figure S6. Bilogarithmic relationship between herbivore biomass (in kg/km2) and annual rainfall (in mm) in 54 African National Parks and Game Reserves [data for herbivore biomass for modern communities from Hatton et al. (2015) and Fick & Hijmans (2017), and for fossil sites from Rodríguez-Gómez et al. (2022); mean annual precipitations calculated by G. Rodríguez-Gómez over the surface of each park using SIG].
